# Supplementary figures and images for: Ribozyme activity modulates the physical properties of RNA–peptide coacervates
Source: eLife. 2023 Jun 16;12:e83543. doi: 10.7554/eLife.83543 (PMC10275638; doi:10.7554/eLife.83543)

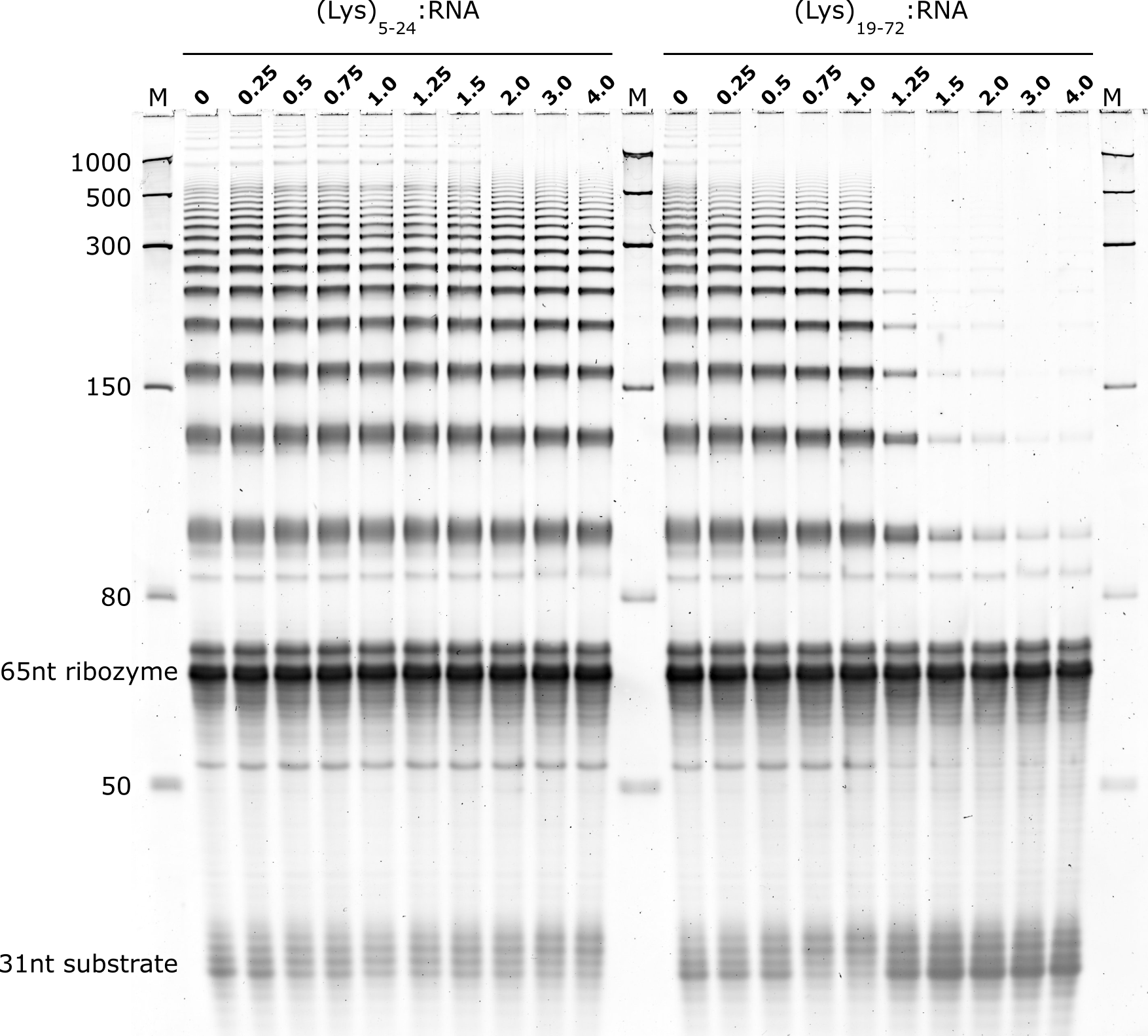

Supplement: Figure 1—source data 1. [file elife-83543-fig1-data1.zip › Figure 1 - source data 1/Figure 1 - source data 1 - labelled gel.png]

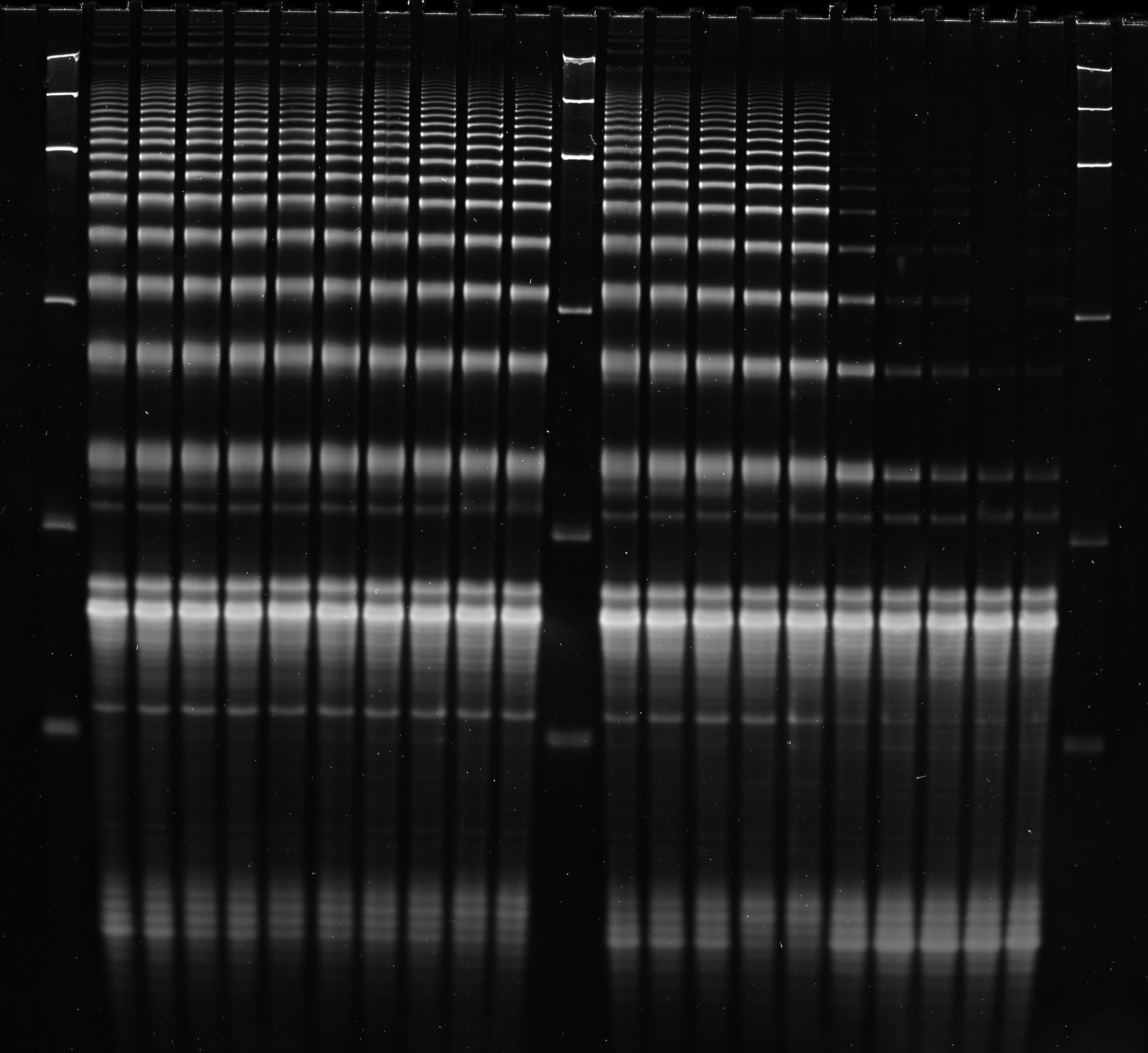

Supplement: Figure 1—source data 1. [file elife-83543-fig1-data1.zip › Figure 1 - source data 1/Figure 1 - source data 1 - raw gel.tif]

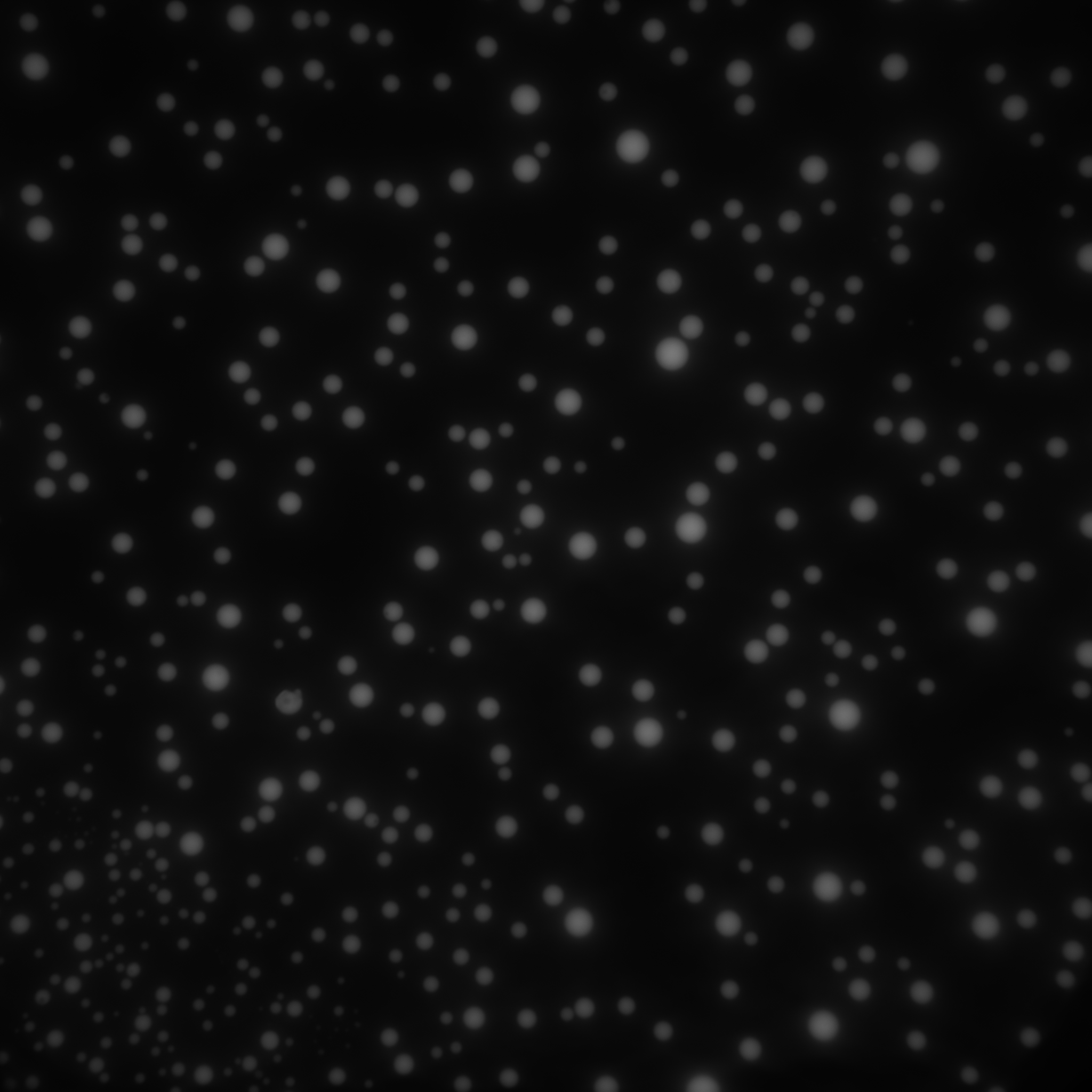

Supplement: Figure 1—source data 3. [file elife-83543-fig1-data3.zip › Figure 1 - source data 3/Figure 1 - source data 3 - active - Lys19-72.tif]

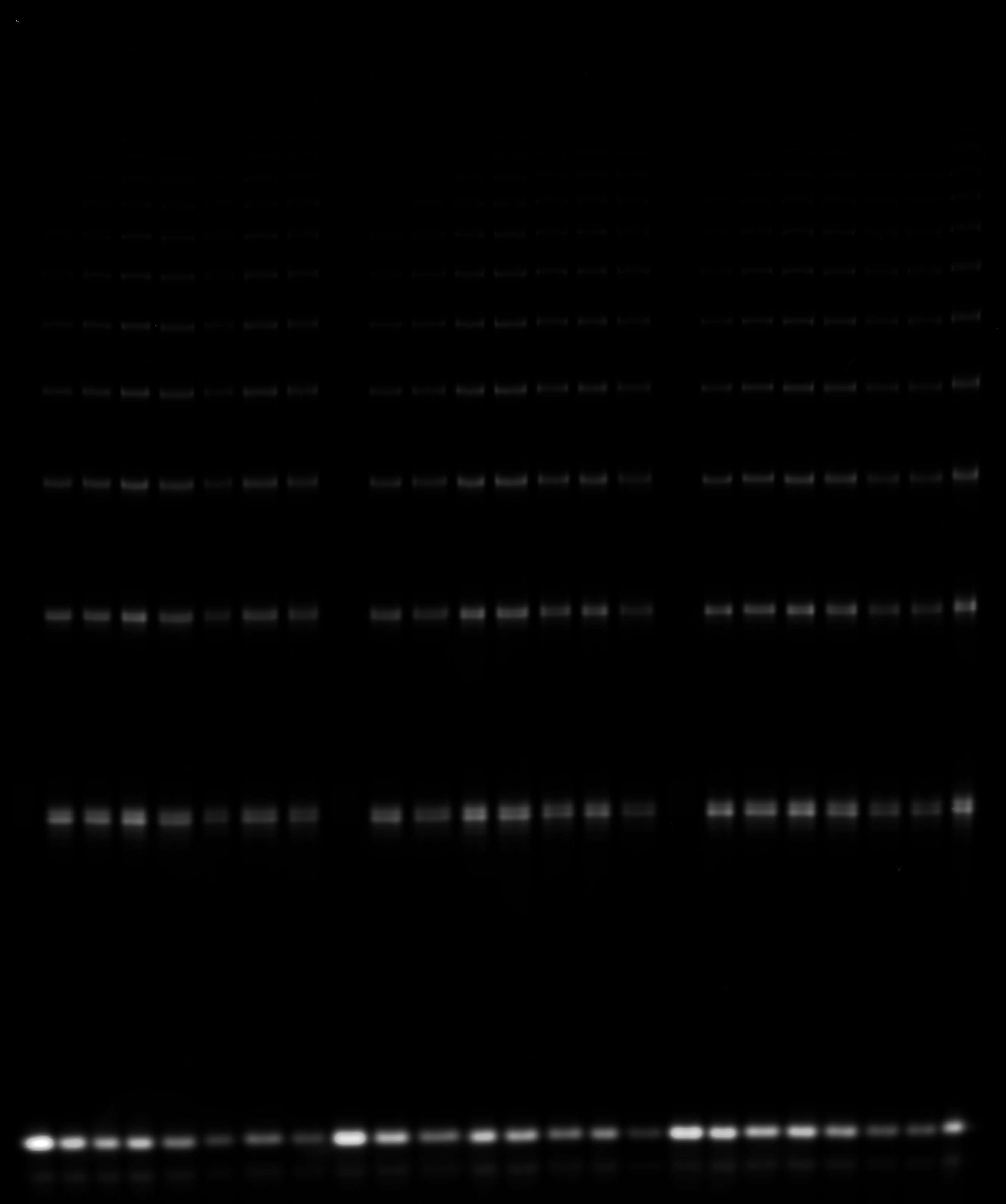

Supplement: Figure 1—source data 4. — Lane identities are listed in the accompanying spreadsheet. [file elife-83543-fig1-data4.zip › Figure 1 - source data 4/Figure 1 - source data 4 - raw kinetics gel 19-72 kDa 0-120min.tif]

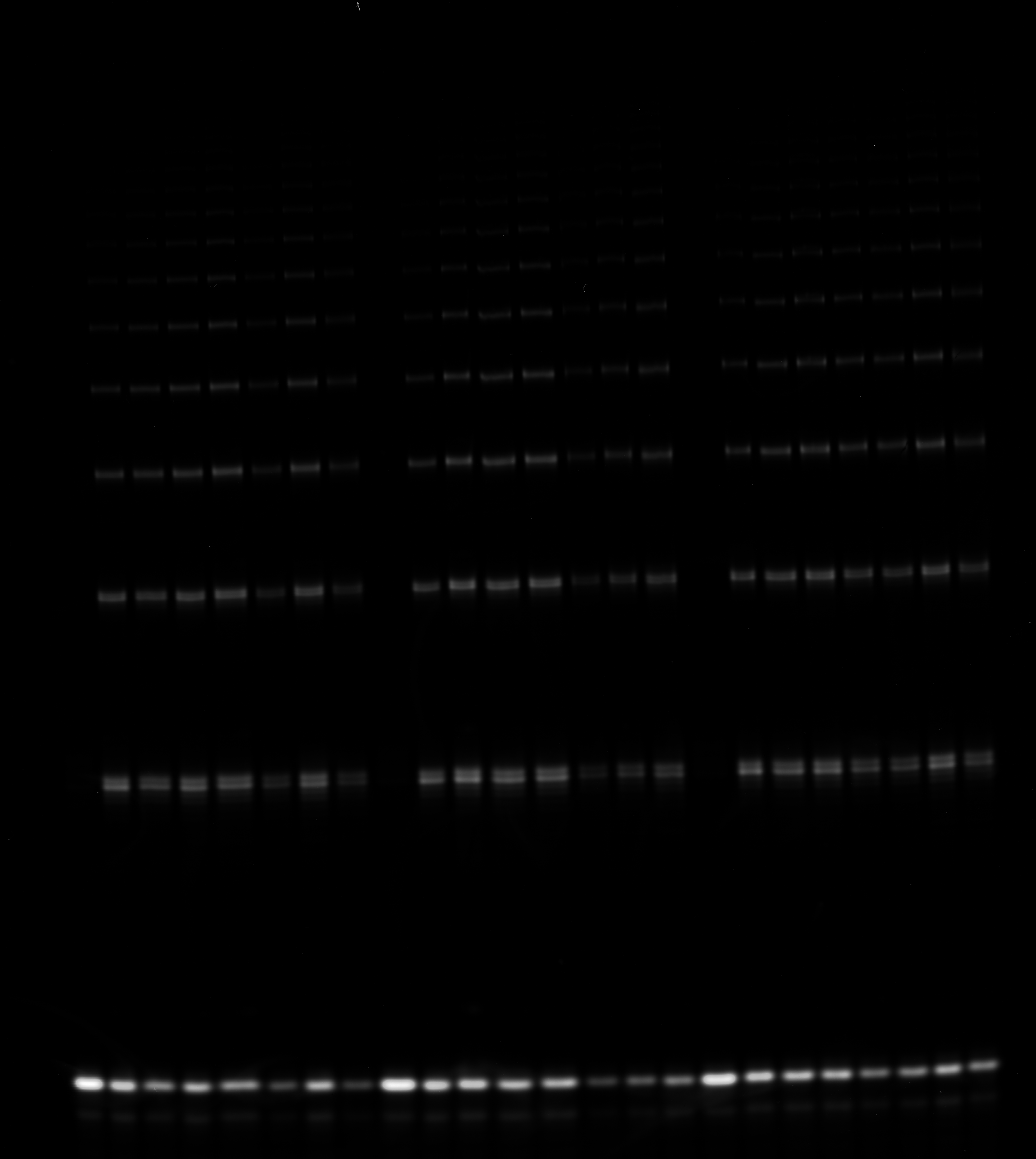

Supplement: Figure 1—source data 4. — Lane identities are listed in the accompanying spreadsheet. [file elife-83543-fig1-data4.zip › Figure 1 - source data 4/Figure 1 - source data 4 - raw kinetics gel 5-24 kDa 0-120min.tif]

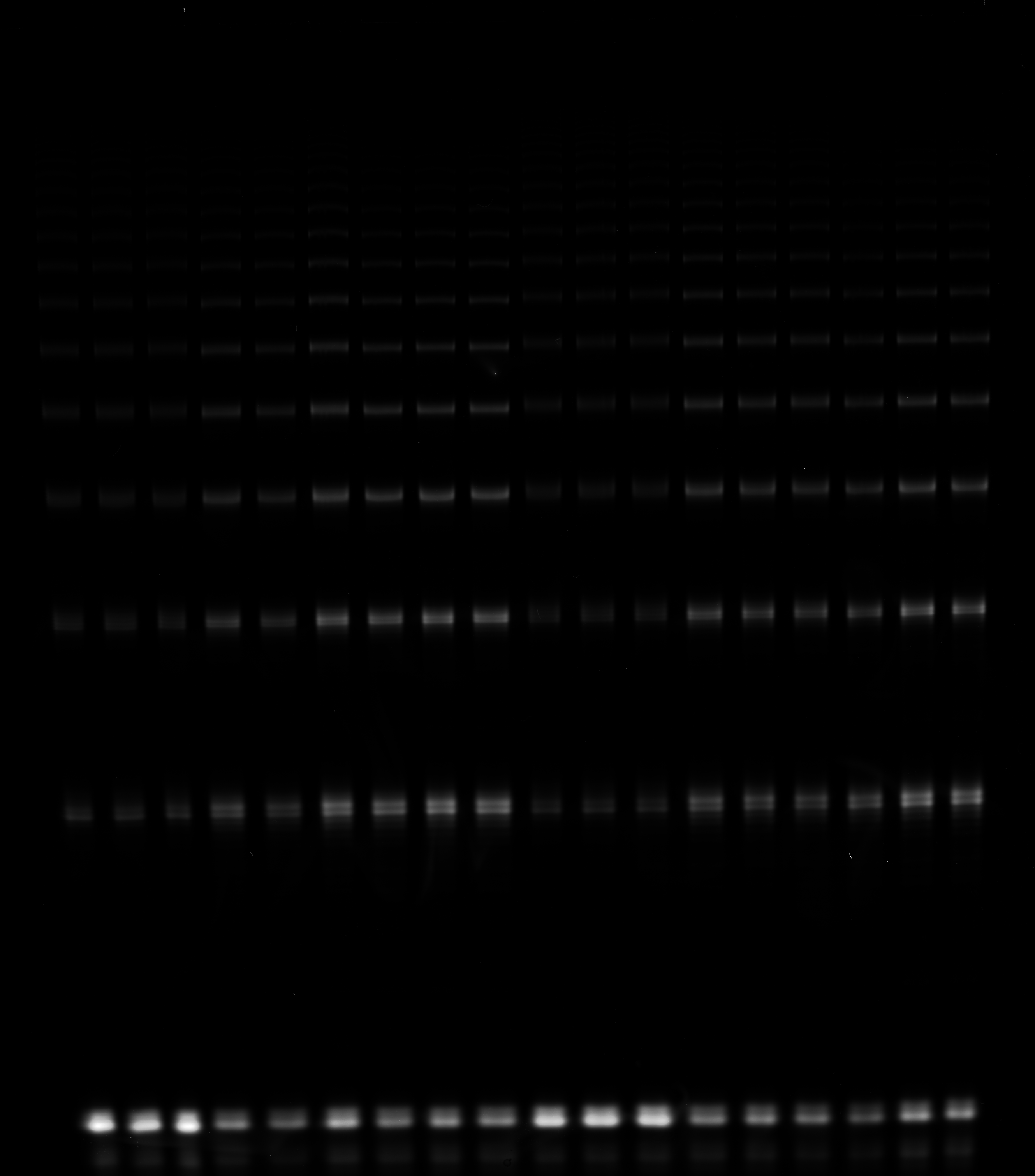

Supplement: Figure 1—source data 4. — Lane identities are listed in the accompanying spreadsheet. [file elife-83543-fig1-data4.zip › Figure 1 - source data 4/Figure 1 - source data 4 - raw kinetics gel all samples 180-240min.tif]

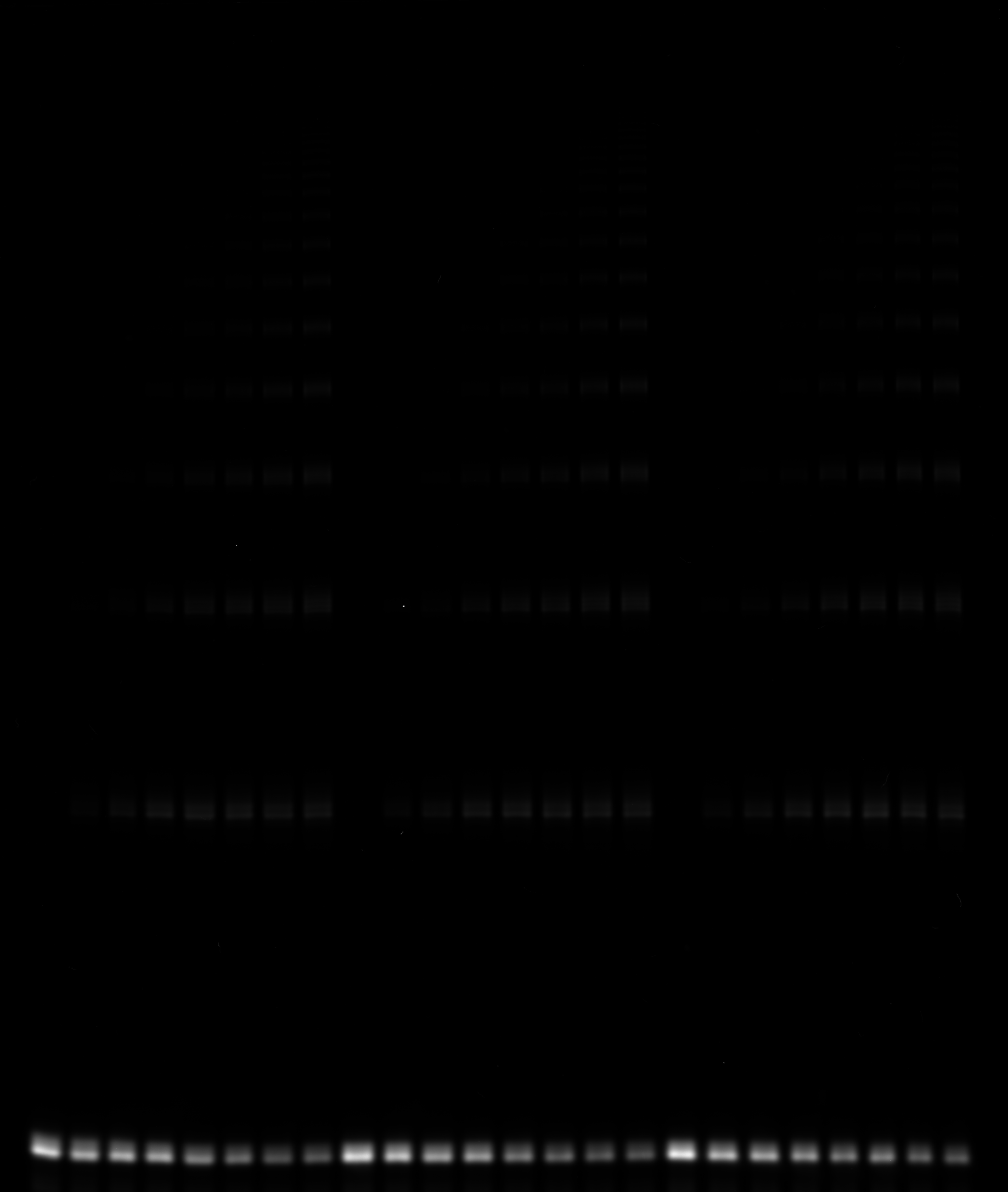

Supplement: Figure 1—source data 4. — Lane identities are listed in the accompanying spreadsheet. [file elife-83543-fig1-data4.zip › Figure 1 - source data 4/Figure 1 - source data 4 - raw kinetics gel solution 0-120min.tif]

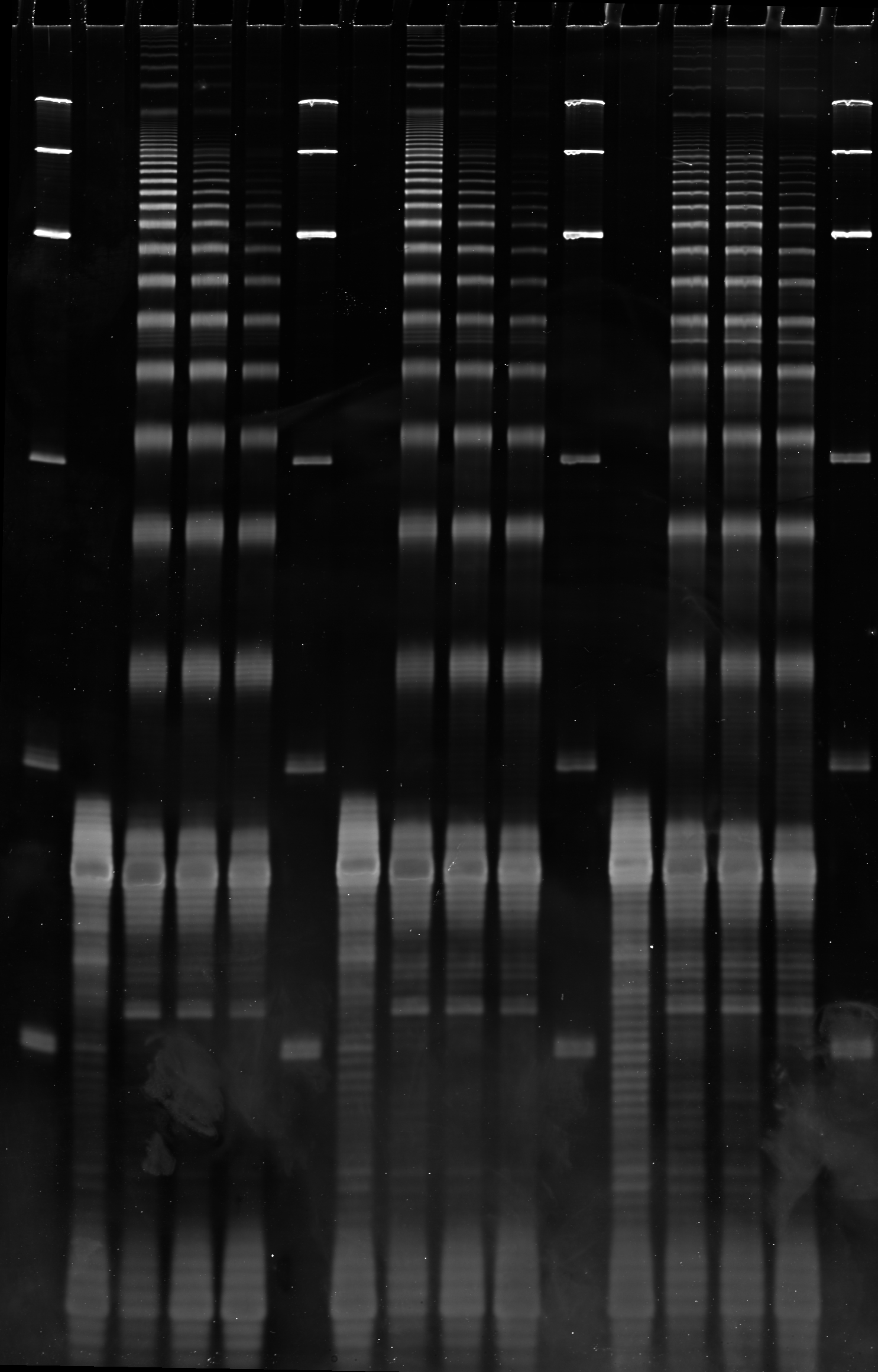

Supplement: Figure 1—figure supplement 1—source data 1. [file elife-83543-fig1-figsupp1-data1.zip › Figure 1 - supplement 1 - source data 1/Figure 1 - supplement 1 - source data 1 - raw.tif]

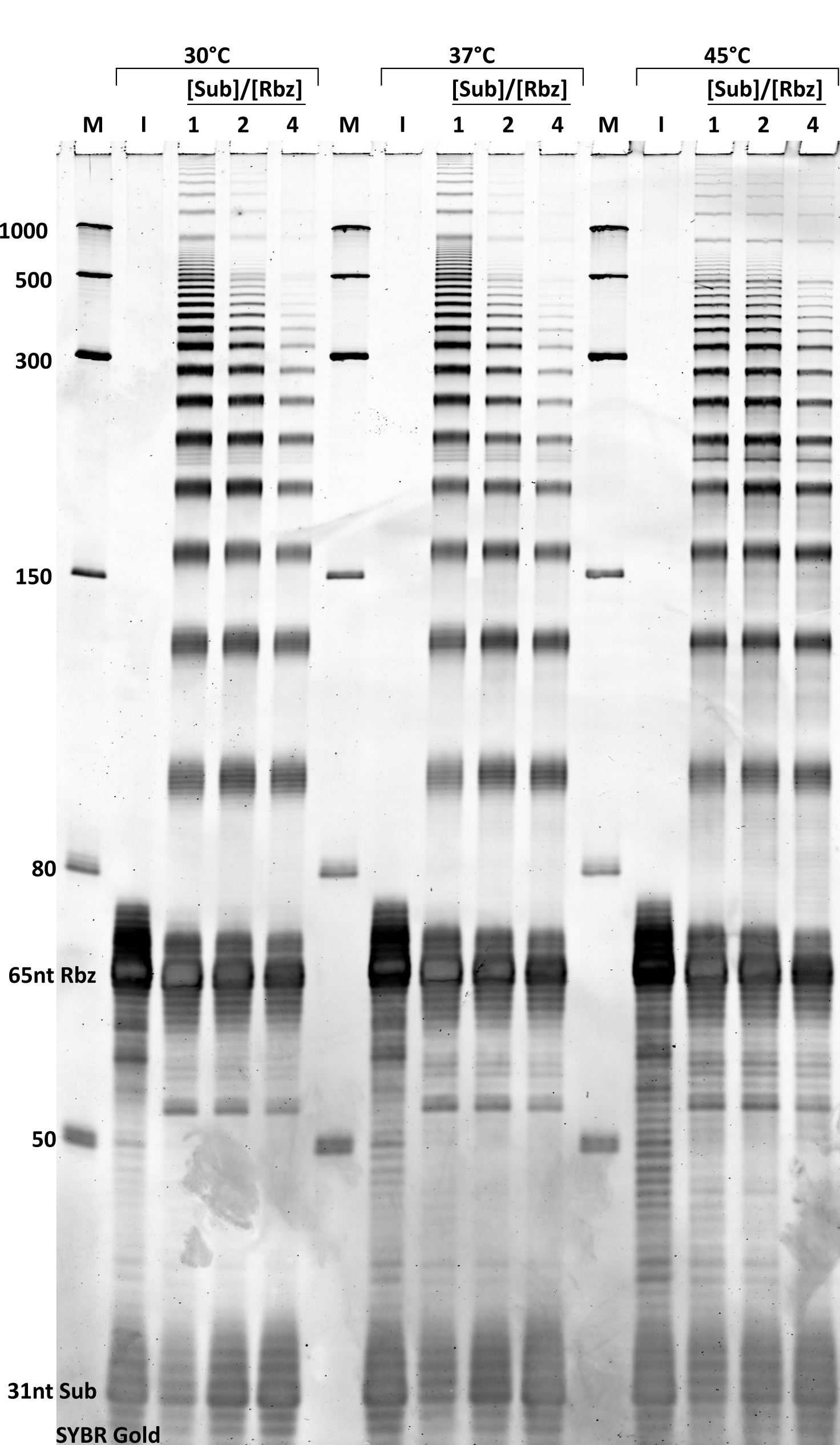

Supplement: Figure 1—figure supplement 1—source data 1. [file elife-83543-fig1-figsupp1-data1.zip › Figure 1 - supplement 1 - source data 1/Figure 1 - supplement 1 - source data 2 - labelled.png]

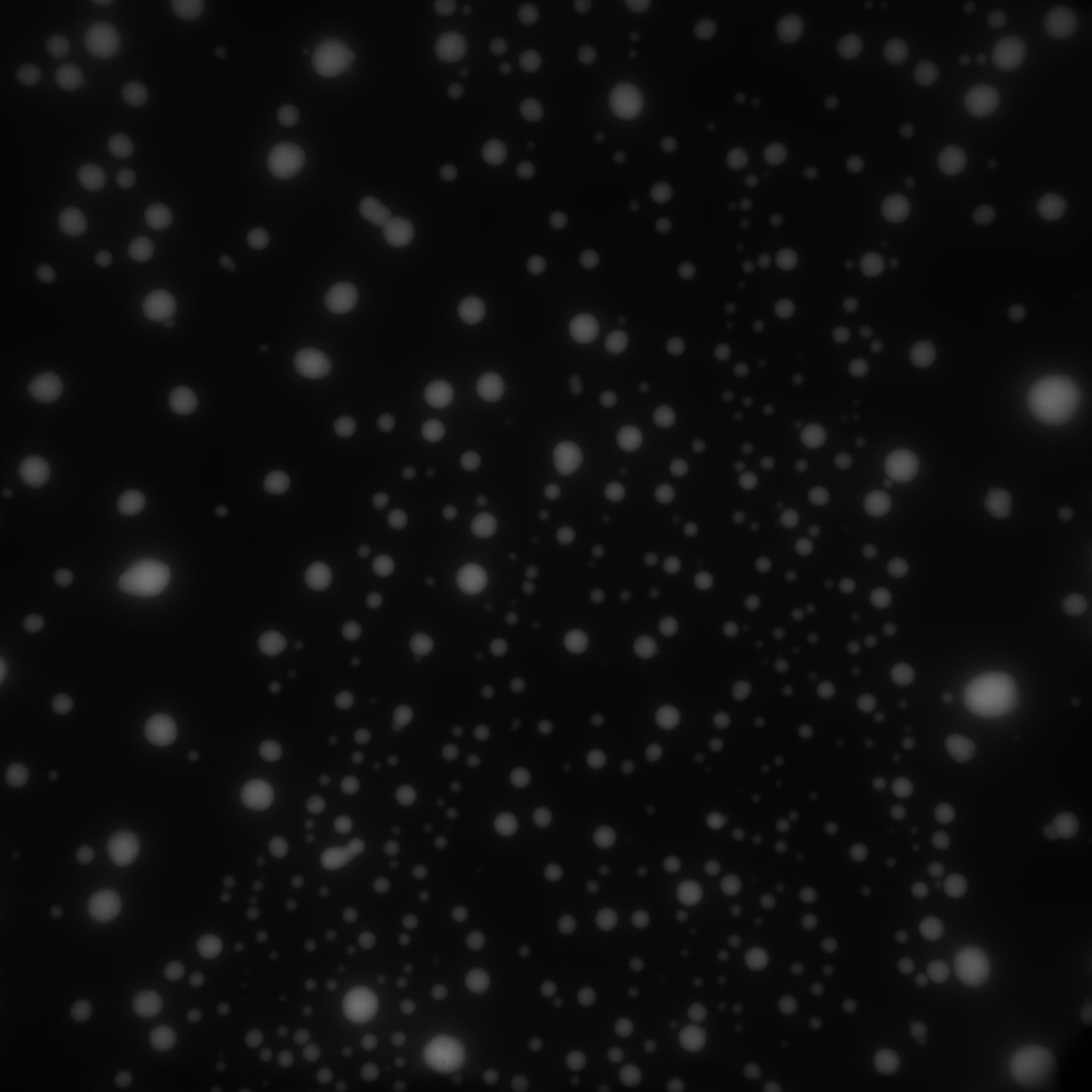

Supplement: Figure 1—figure supplement 2—source data 3. [file elife-83543-fig1-figsupp2-data3.zip › Figure 1 - supplement 2 - source data 3/Figure 1 - supplement 2 -source data 3 - active - Lys5-24.tif]

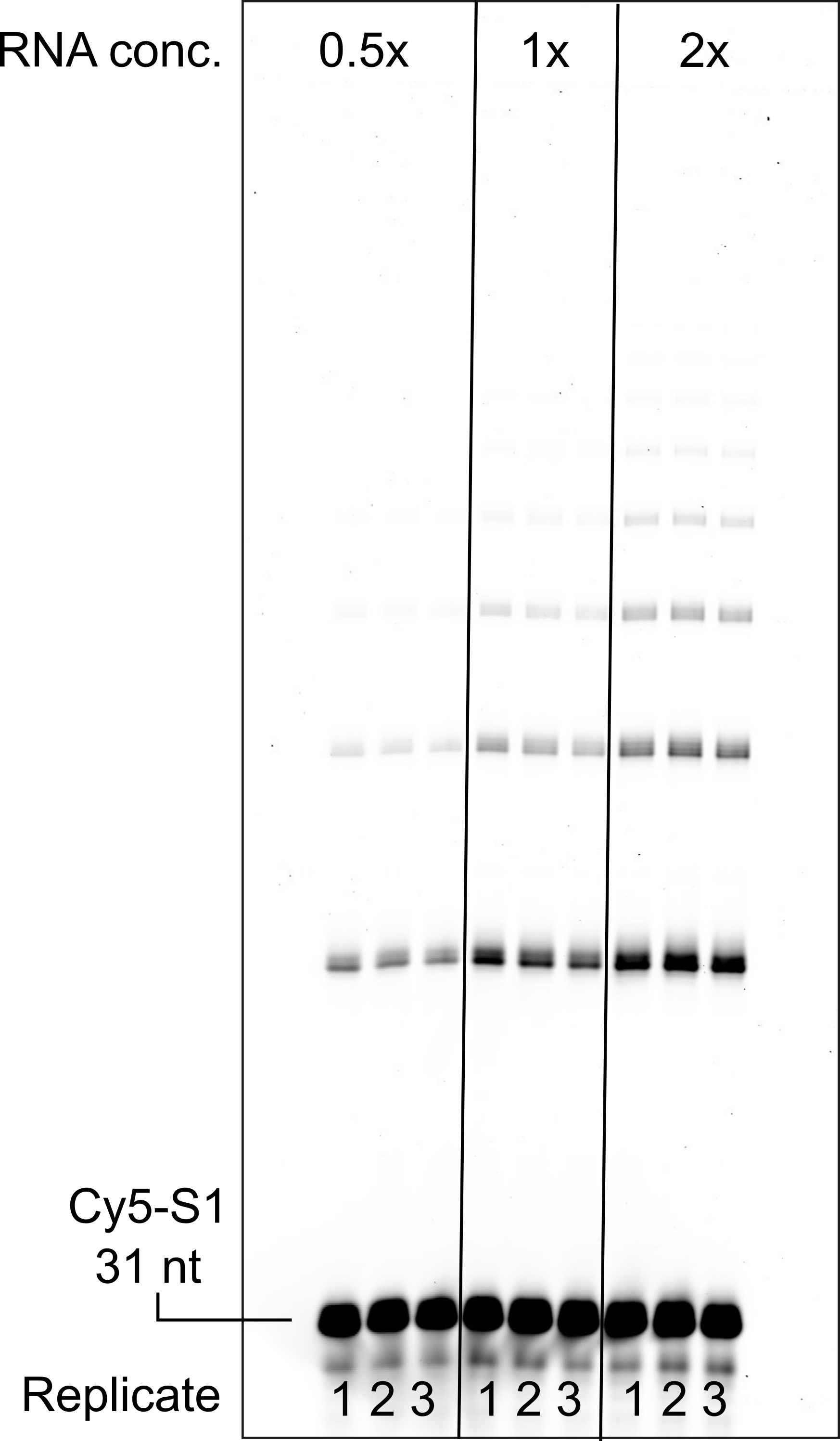

Supplement: Figure 1—figure supplement 4—source data 1. [file elife-83543-fig1-figsupp4-data1.zip › Figure 1 - supplement 4 - source data 1/Figure1_Supplement4a_Labelled.png]

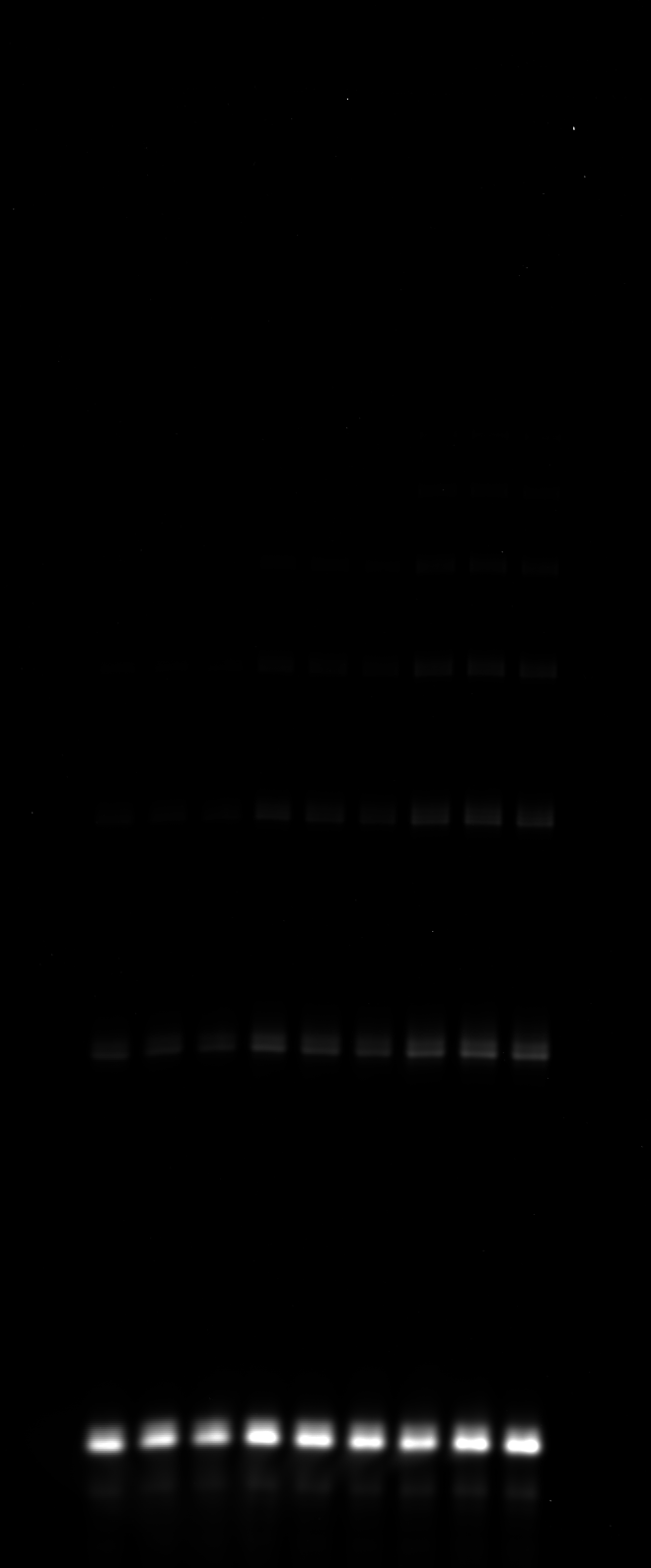

Supplement: Figure 1—figure supplement 4—source data 1. [file elife-83543-fig1-figsupp4-data1.zip › Figure 1 - supplement 4 - source data 1/Figure1_Supplement4a_Raw.tif]

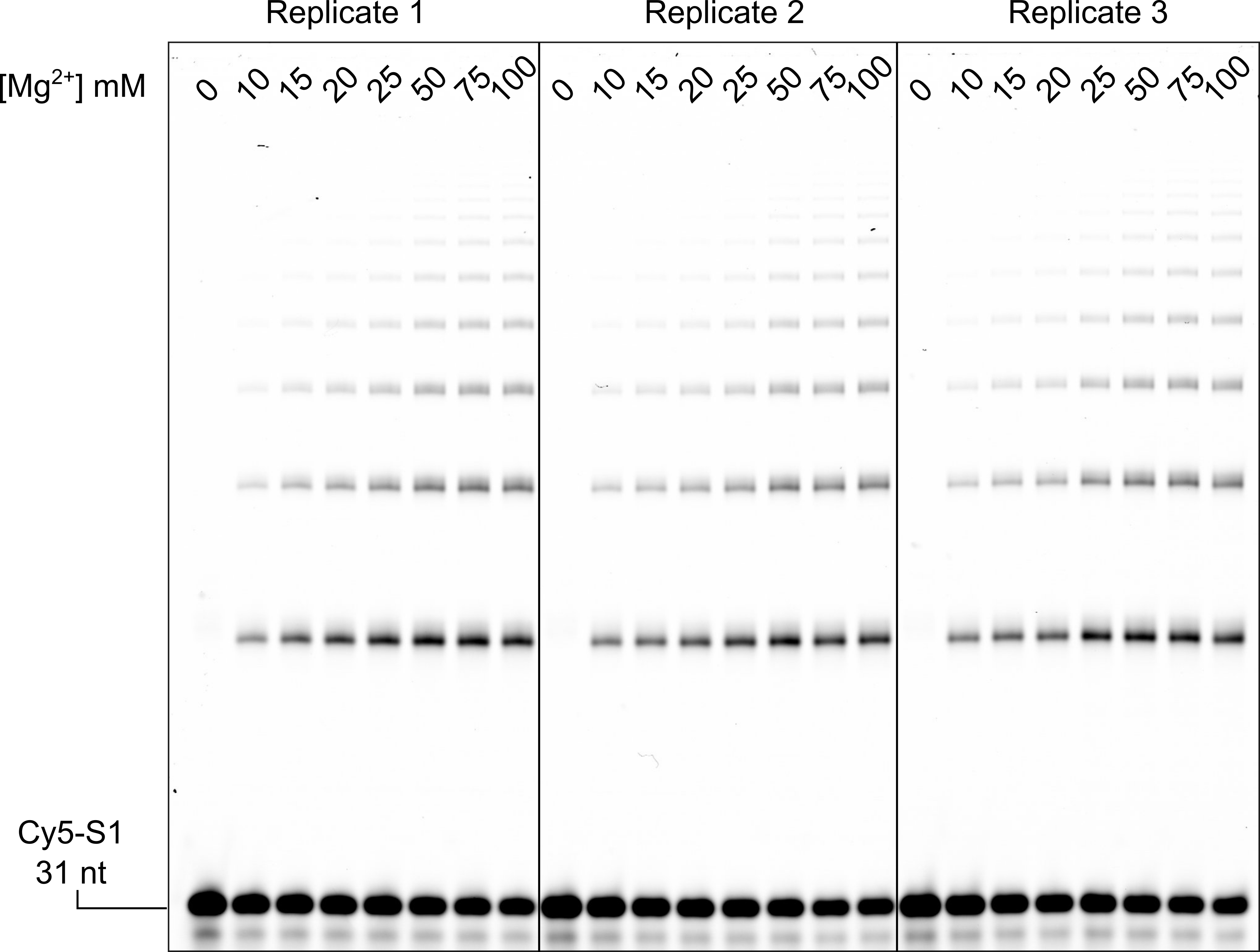

Supplement: Figure 1—figure supplement 4—source data 1. [file elife-83543-fig1-figsupp4-data1.zip › Figure 1 - supplement 4 - source data 1/Figure1_Supplement4b_Labelled.png]

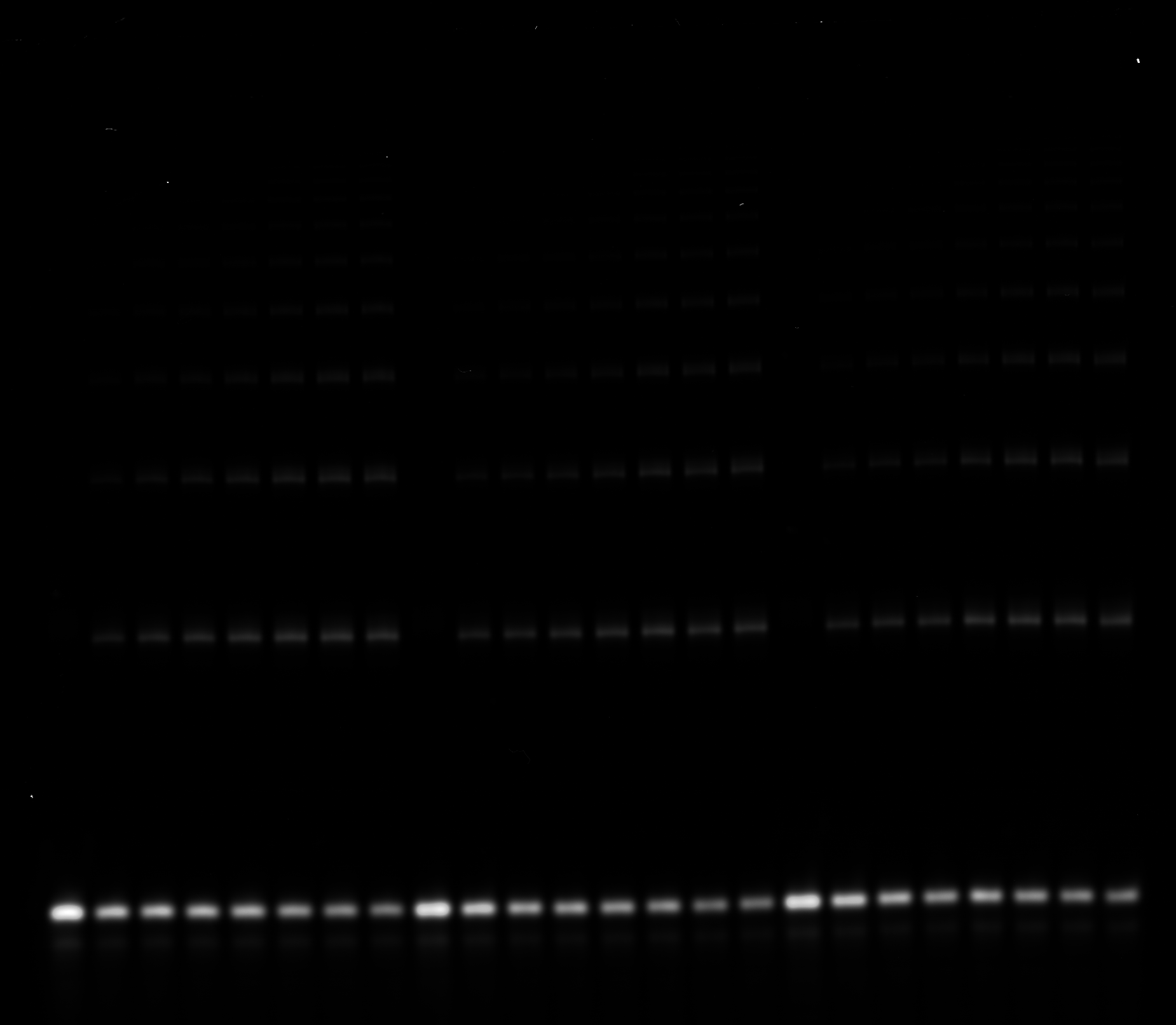

Supplement: Figure 1—figure supplement 4—source data 1. [file elife-83543-fig1-figsupp4-data1.zip › Figure 1 - supplement 4 - source data 1/Figure1_Supplement4b_Raw.tif]

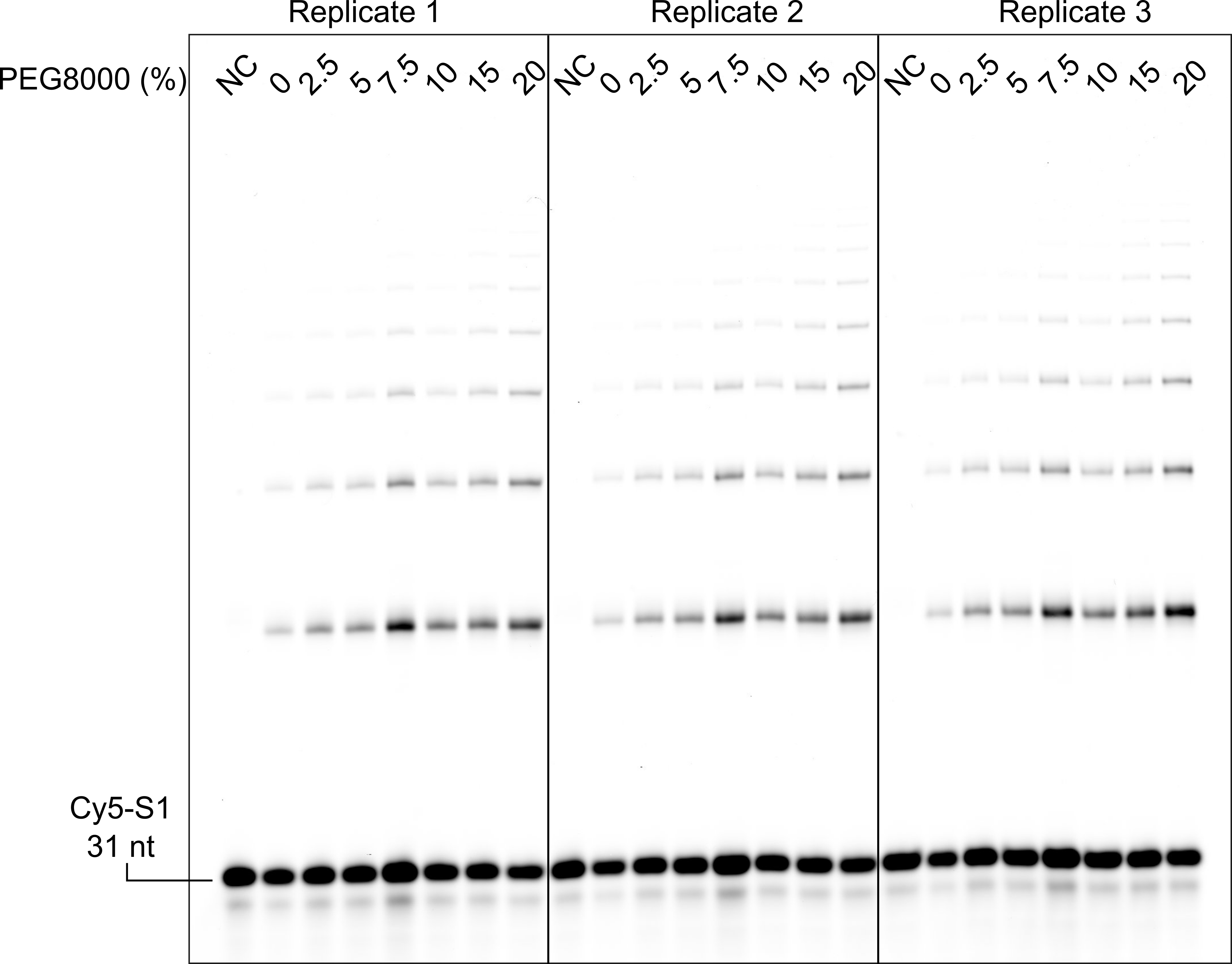

Supplement: Figure 1—figure supplement 4—source data 1. [file elife-83543-fig1-figsupp4-data1.zip › Figure 1 - supplement 4 - source data 1/Figure1_Supplement4c_Labelled.png]

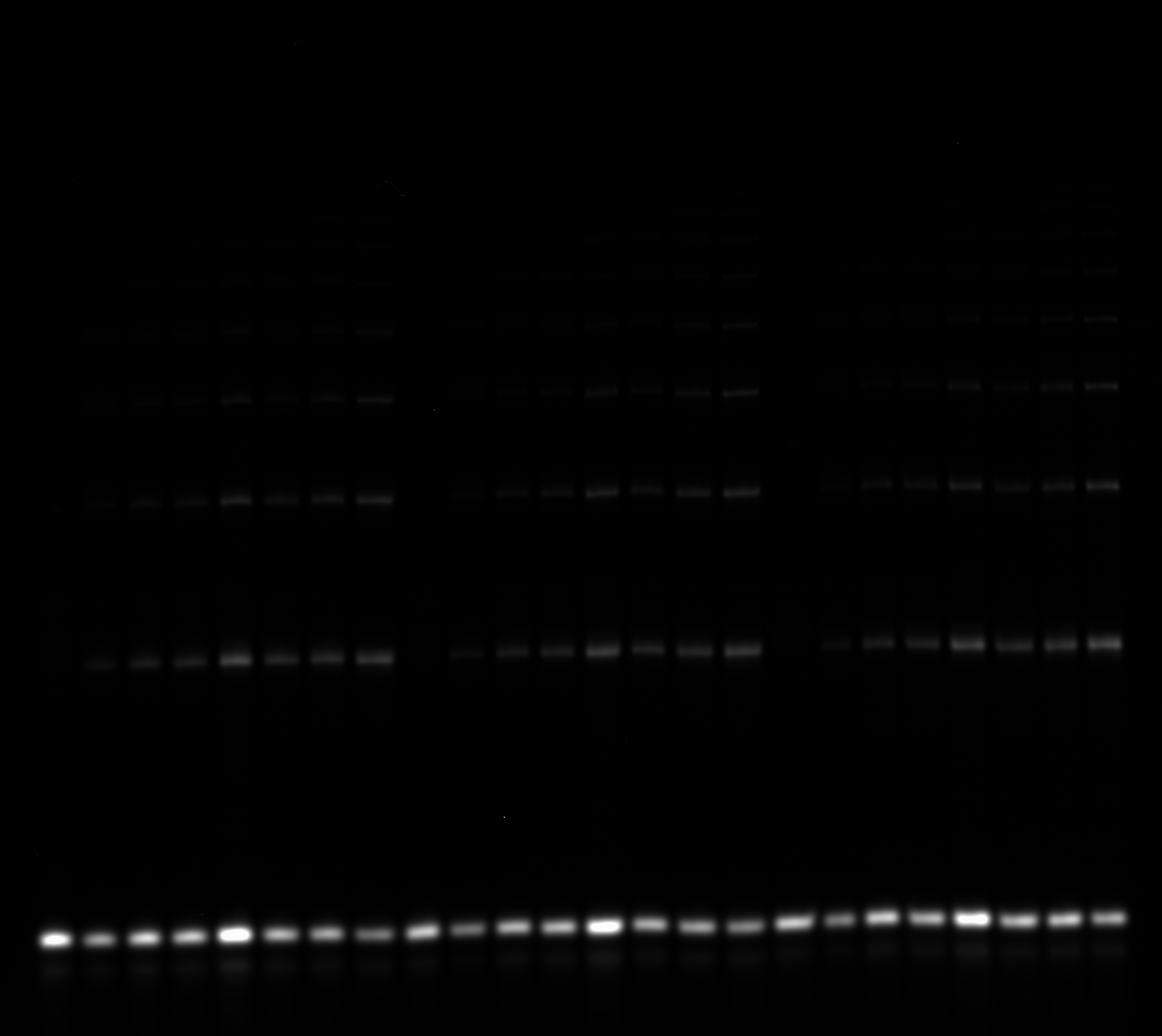

Supplement: Figure 1—figure supplement 4—source data 1. [file elife-83543-fig1-figsupp4-data1.zip › Figure 1 - supplement 4 - source data 1/Figure1_Supplement4c_Raw.tif]

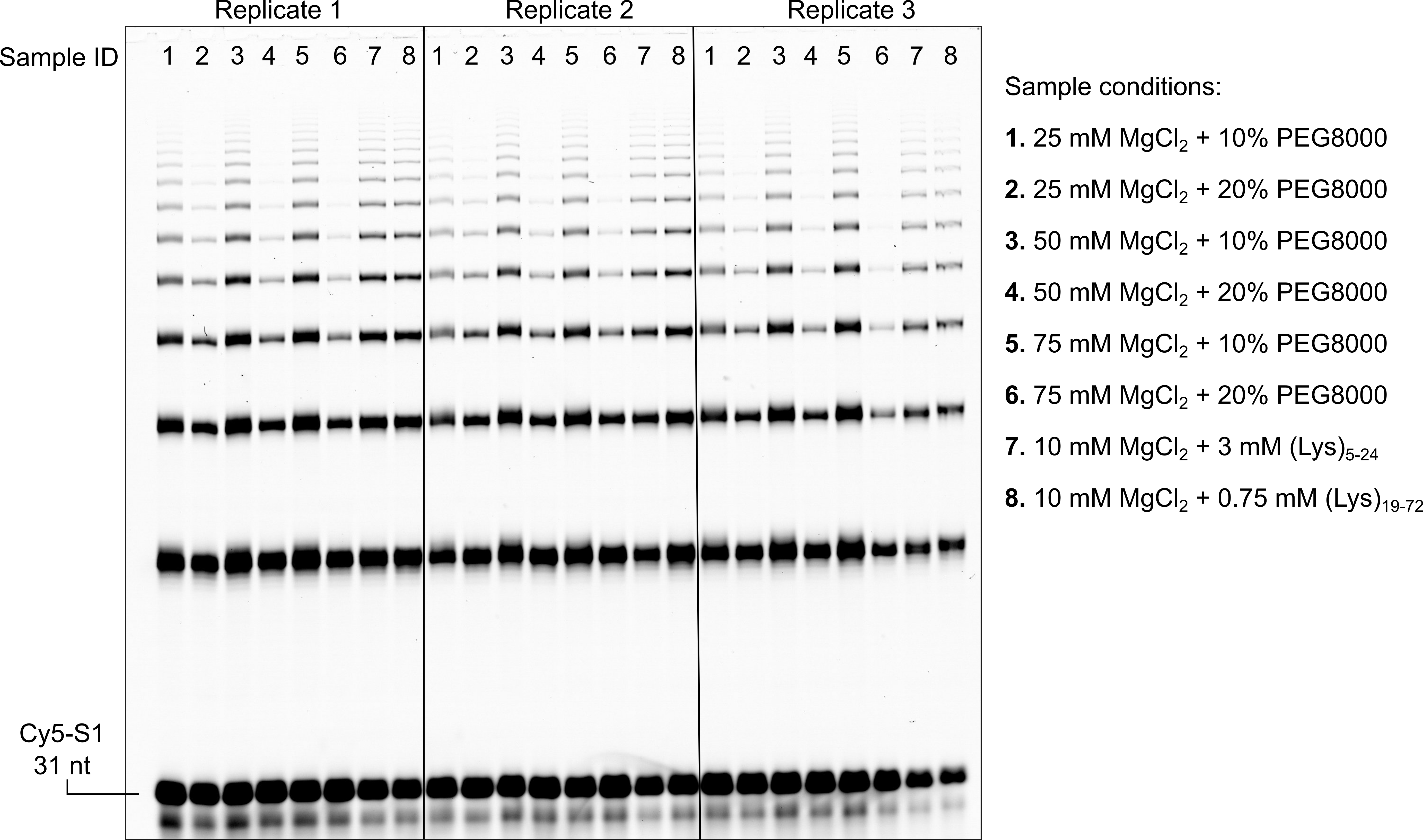

Supplement: Figure 1—figure supplement 4—source data 1. [file elife-83543-fig1-figsupp4-data1.zip › Figure 1 - supplement 4 - source data 1/Figure1_Supplement4d_Labelled.png]

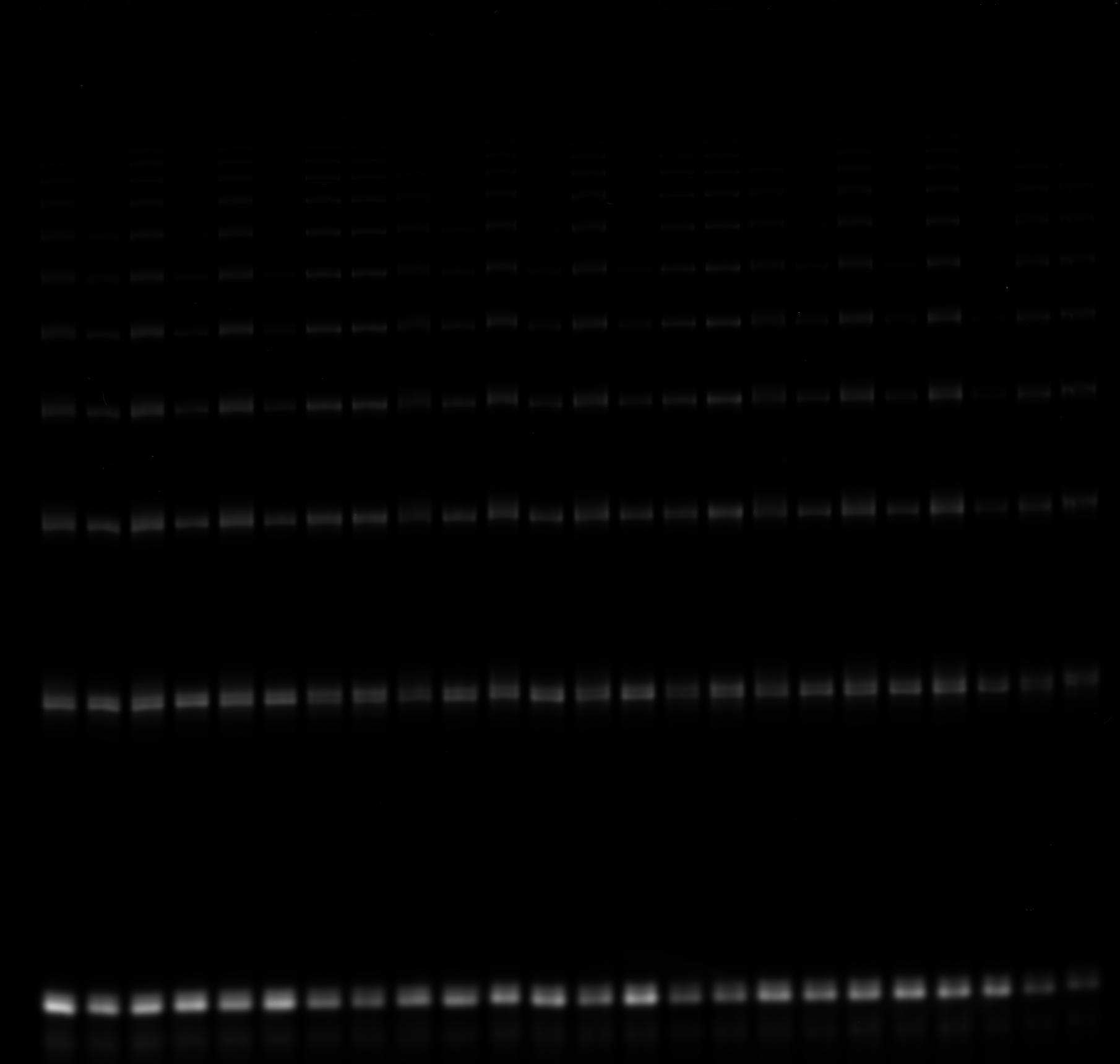

Supplement: Figure 1—figure supplement 4—source data 1. [file elife-83543-fig1-figsupp4-data1.zip › Figure 1 - supplement 4 - source data 1/Figure1_Supplement4d_Raw.tif]

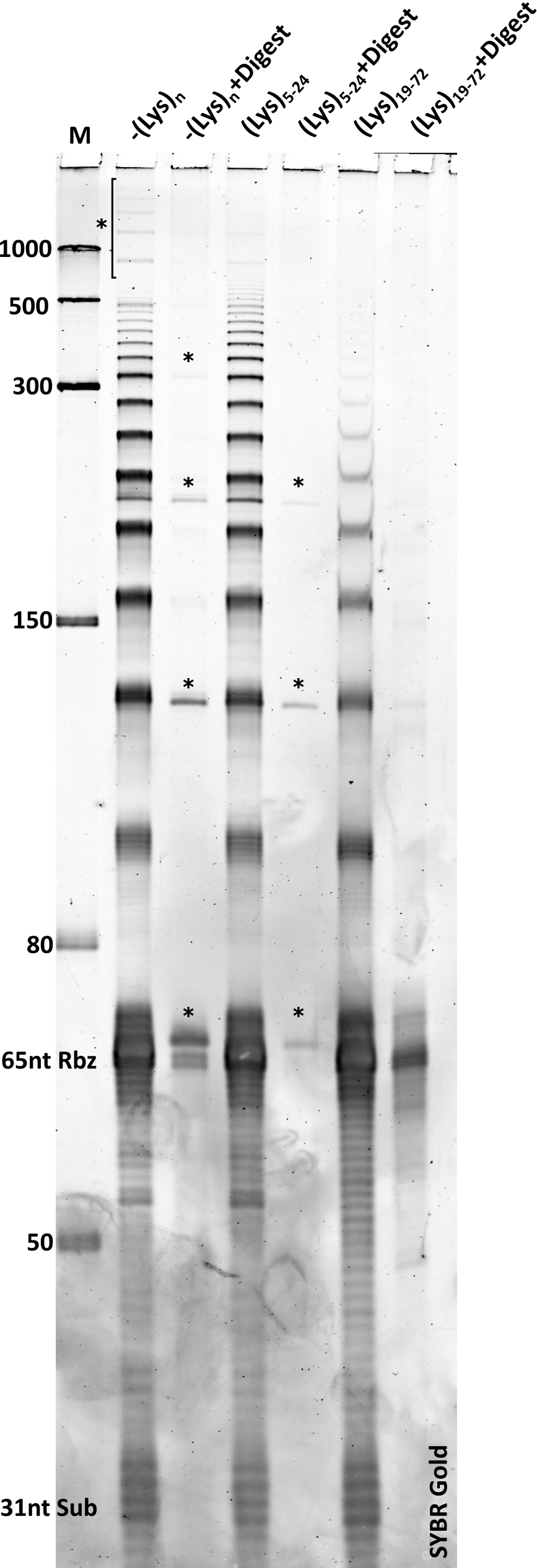

Supplement: Figure 1—figure supplement 5—source data 1. [file elife-83543-fig1-figsupp5-data1.zip › Figure 1 - supplement 5 - source data 1/Figure 1 - supplement 3 - source data 1/Figure 1 - supplement 3 - source data 1 - labelled gel.png]

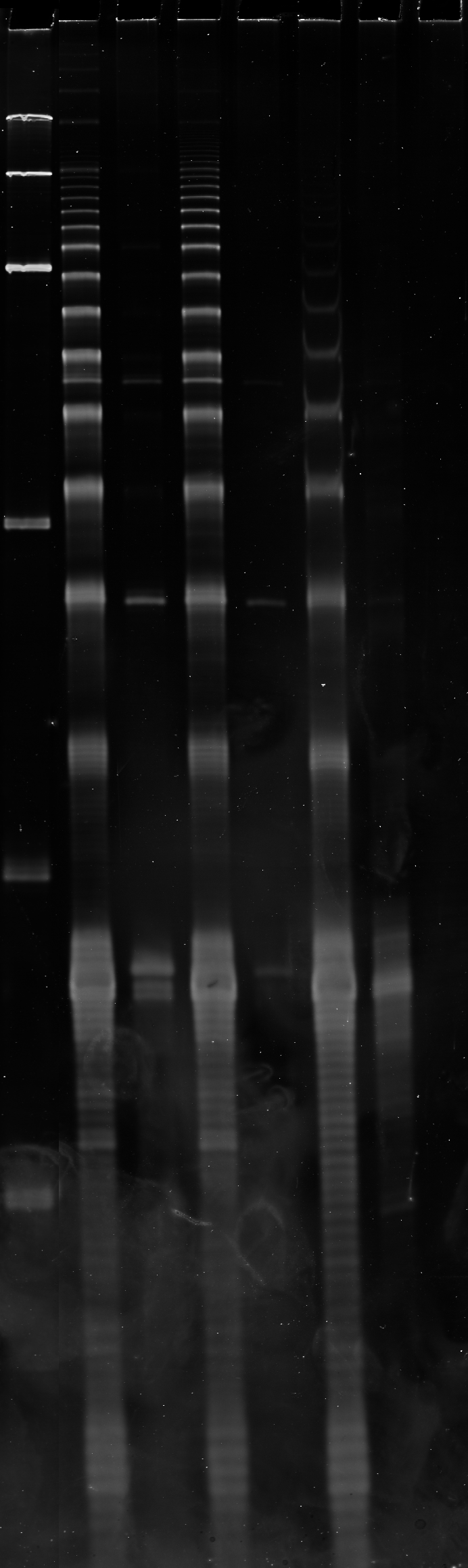

Supplement: Figure 1—figure supplement 5—source data 1. [file elife-83543-fig1-figsupp5-data1.zip › Figure 1 - supplement 5 - source data 1/Figure 1 - supplement 3 - source data 1/Figure 1 - supplement 3 - source data 1 - raw gel.tif]

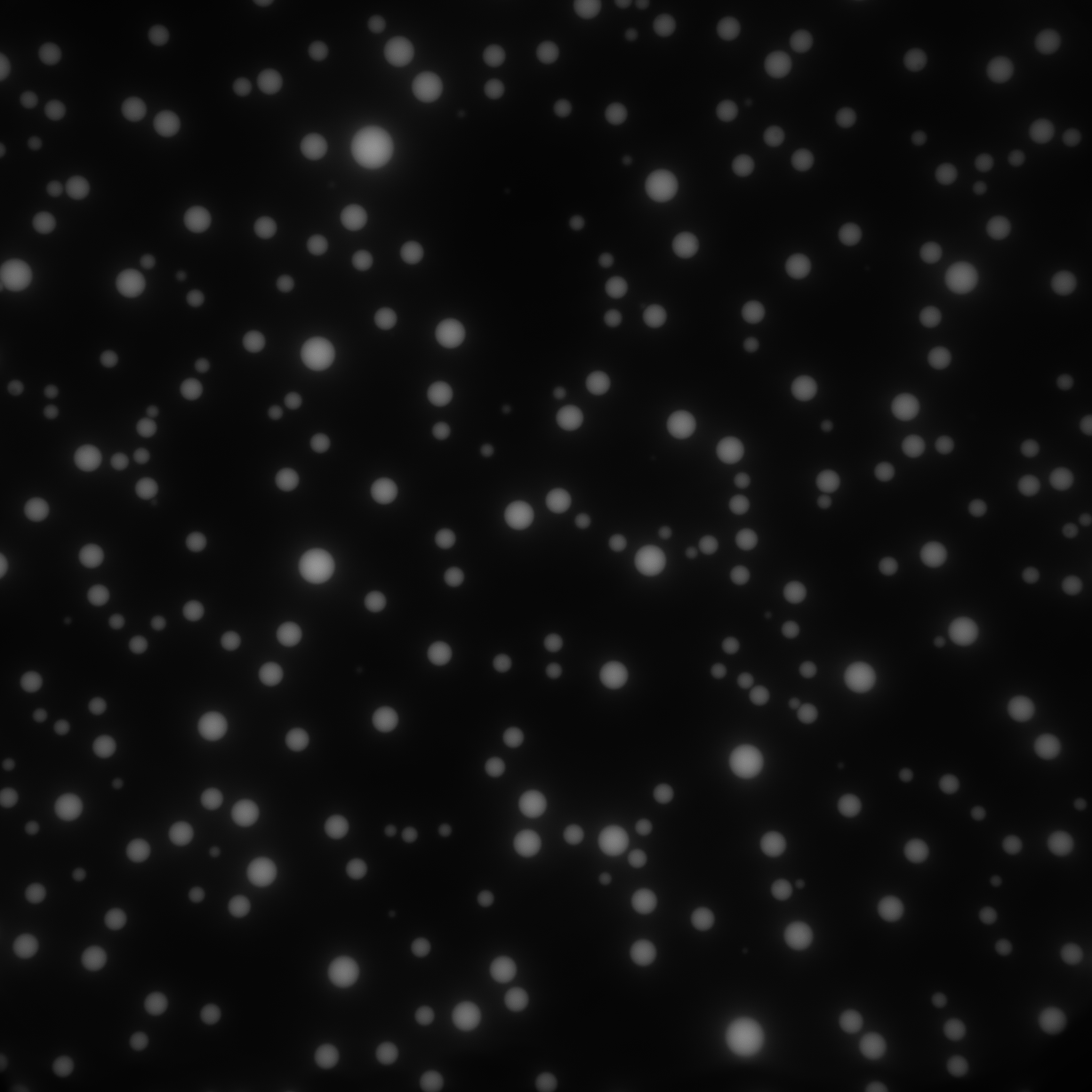

Supplement: Figure 2—source data 1. — Extracted numerical parameters are listed in the accompanying spreadsheet. [file elife-83543-fig2-data1.zip › Figure 2 - source data 1/Figure 2 - source data 1 - active ribozyme - Lys19-72 -1 h.tif]

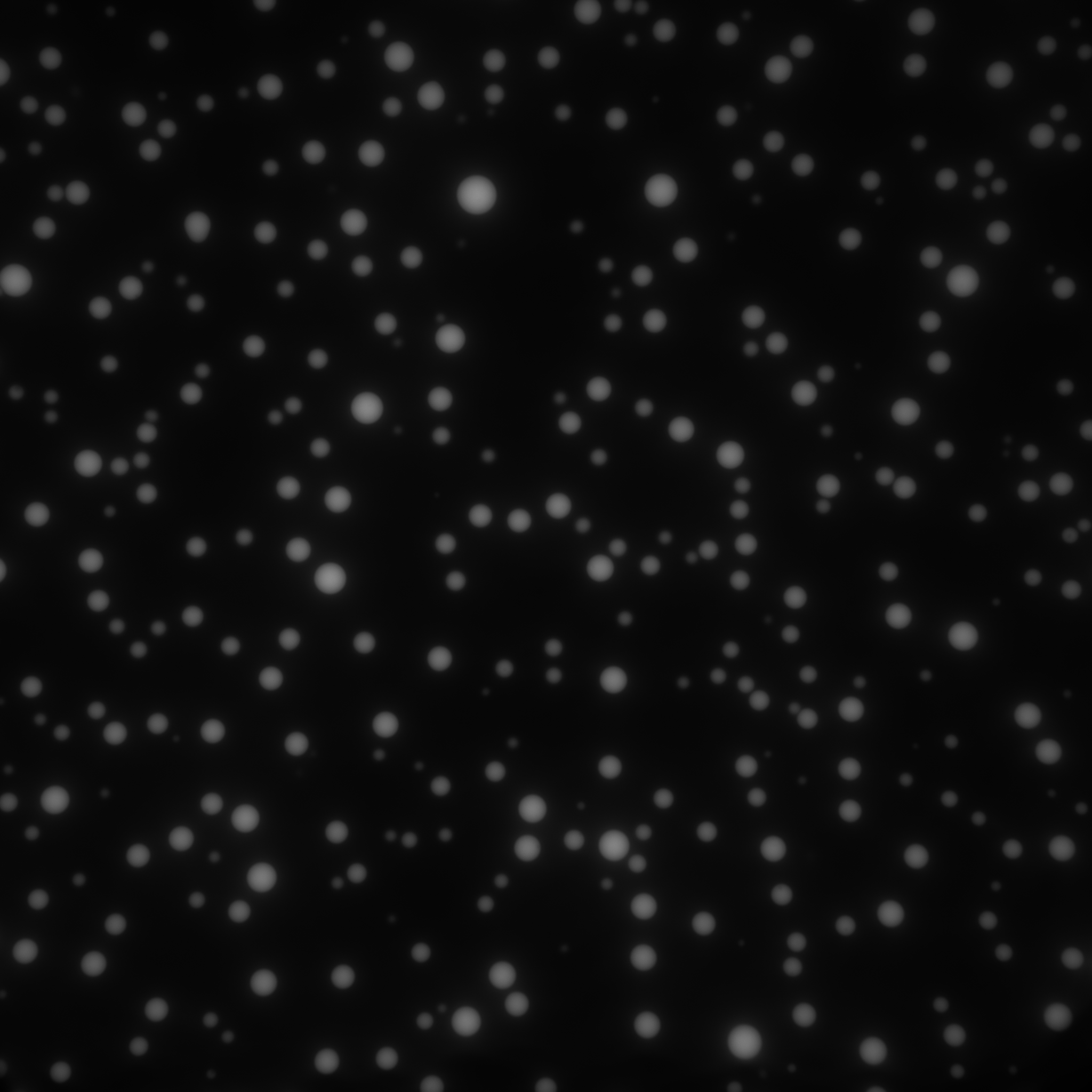

Supplement: Figure 2—source data 1. — Extracted numerical parameters are listed in the accompanying spreadsheet. [file elife-83543-fig2-data1.zip › Figure 2 - source data 1/Figure 2 - source data 1 - active ribozyme - Lys19-72 - 0.5 h.tif]

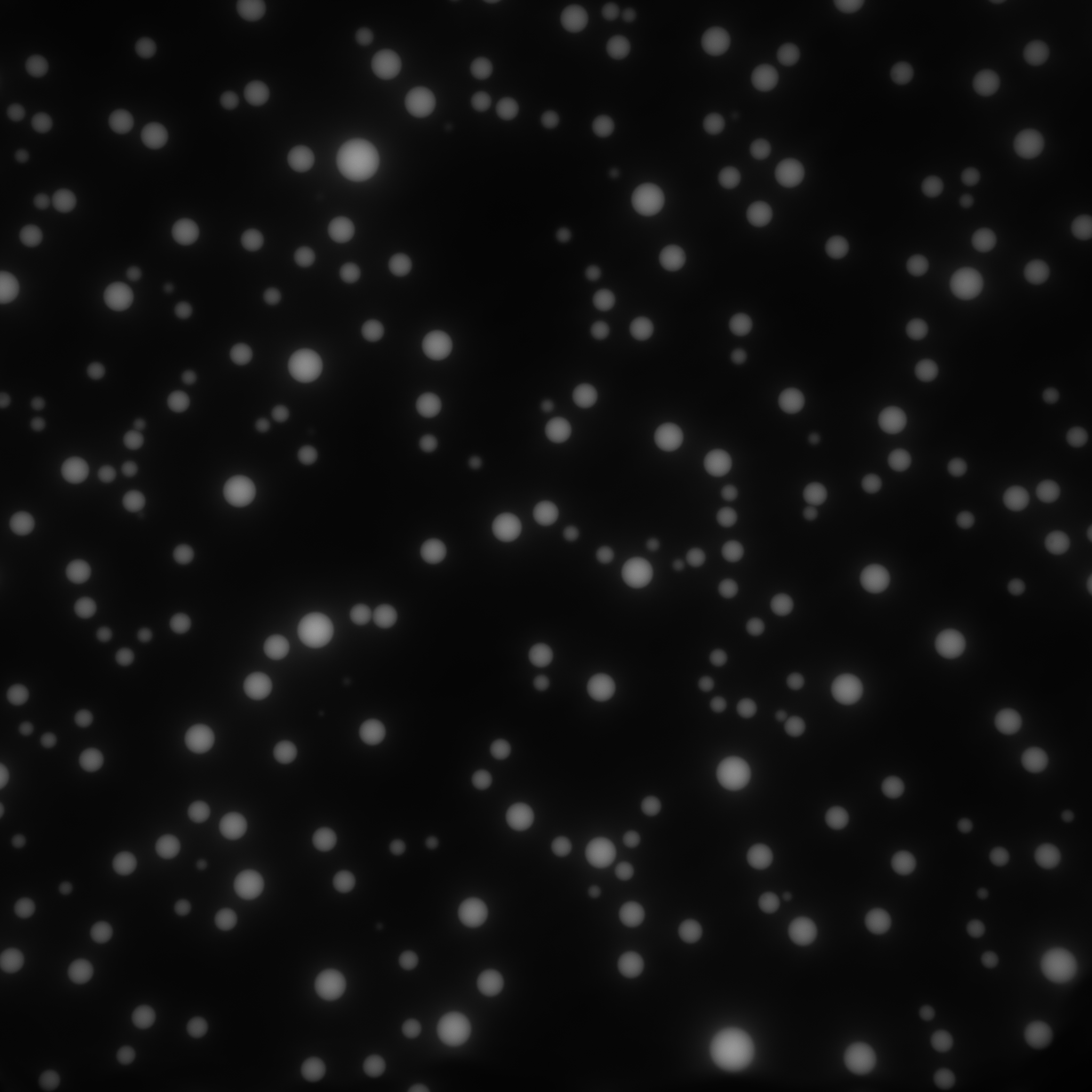

Supplement: Figure 2—source data 1. — Extracted numerical parameters are listed in the accompanying spreadsheet. [file elife-83543-fig2-data1.zip › Figure 2 - source data 1/Figure 2 - source data 1 - active ribozyme - Lys19-72 - 2 h.tif]

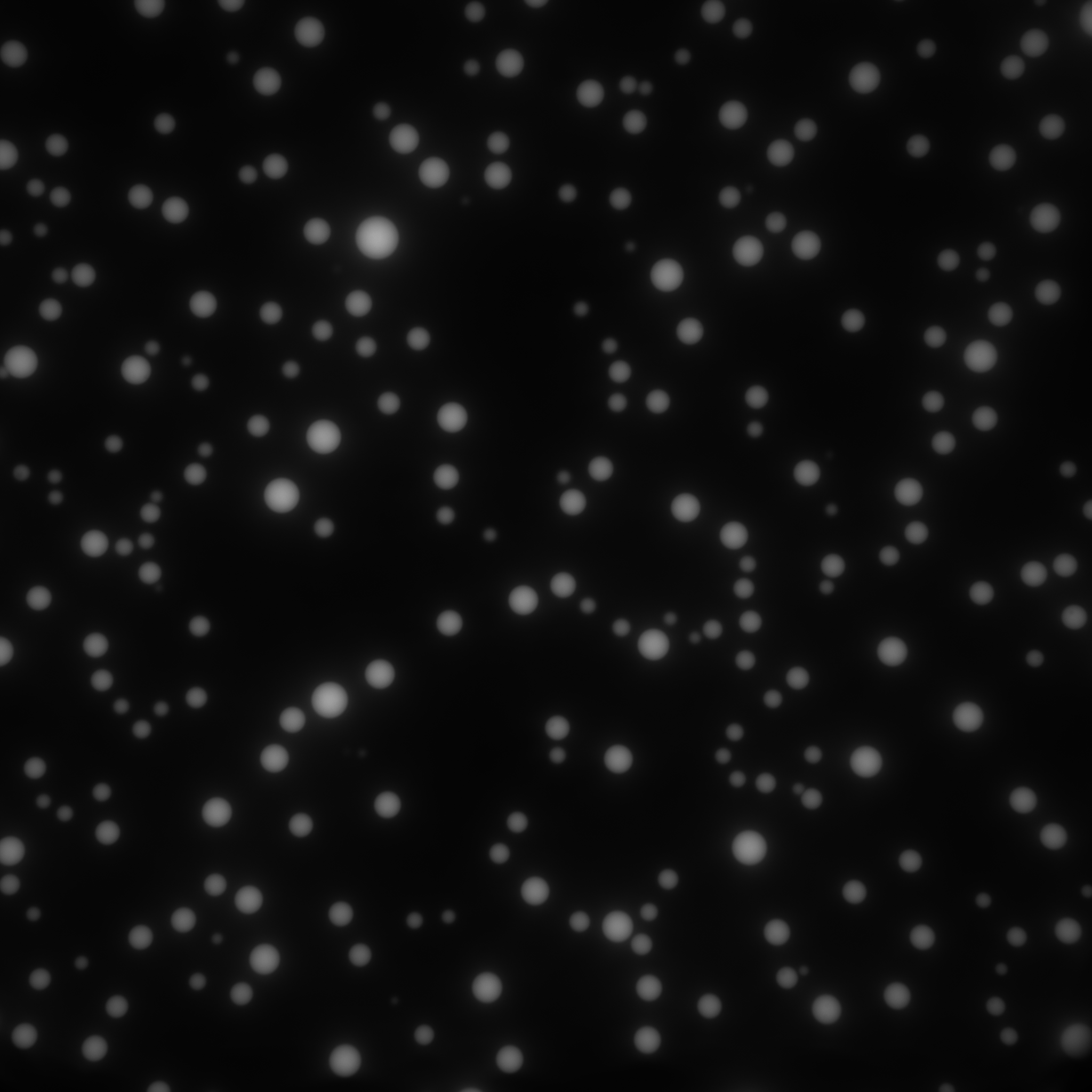

Supplement: Figure 2—source data 1. — Extracted numerical parameters are listed in the accompanying spreadsheet. [file elife-83543-fig2-data1.zip › Figure 2 - source data 1/Figure 2 - source data 1 - active ribozyme - Lys19-72 - 24 h.tif]

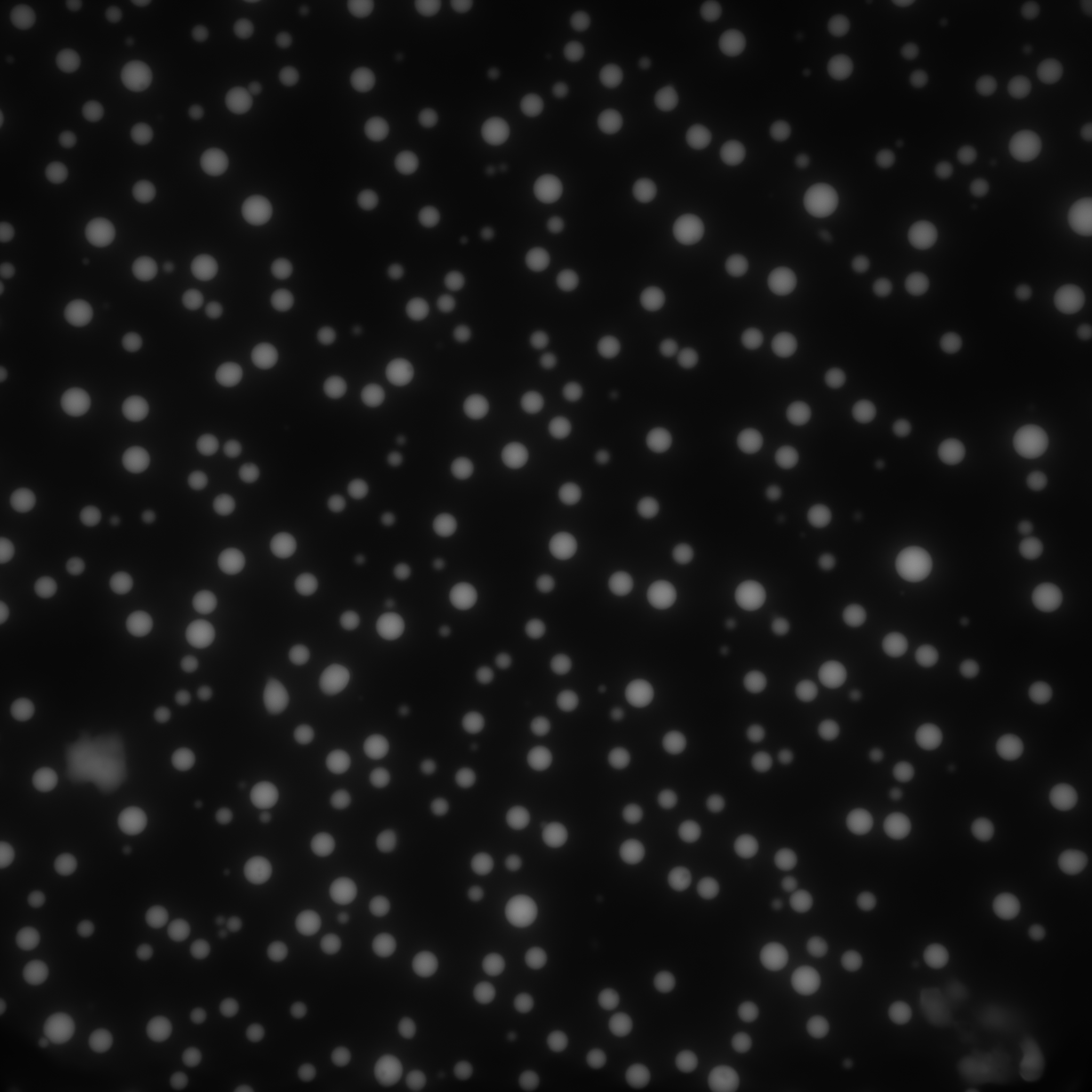

Supplement: Figure 2—source data 1. — Extracted numerical parameters are listed in the accompanying spreadsheet. [file elife-83543-fig2-data1.zip › Figure 2 - source data 1/Figure 2 - source data 1 - inactive ribozyme - Lys19-72 - 0.5 h.tif]

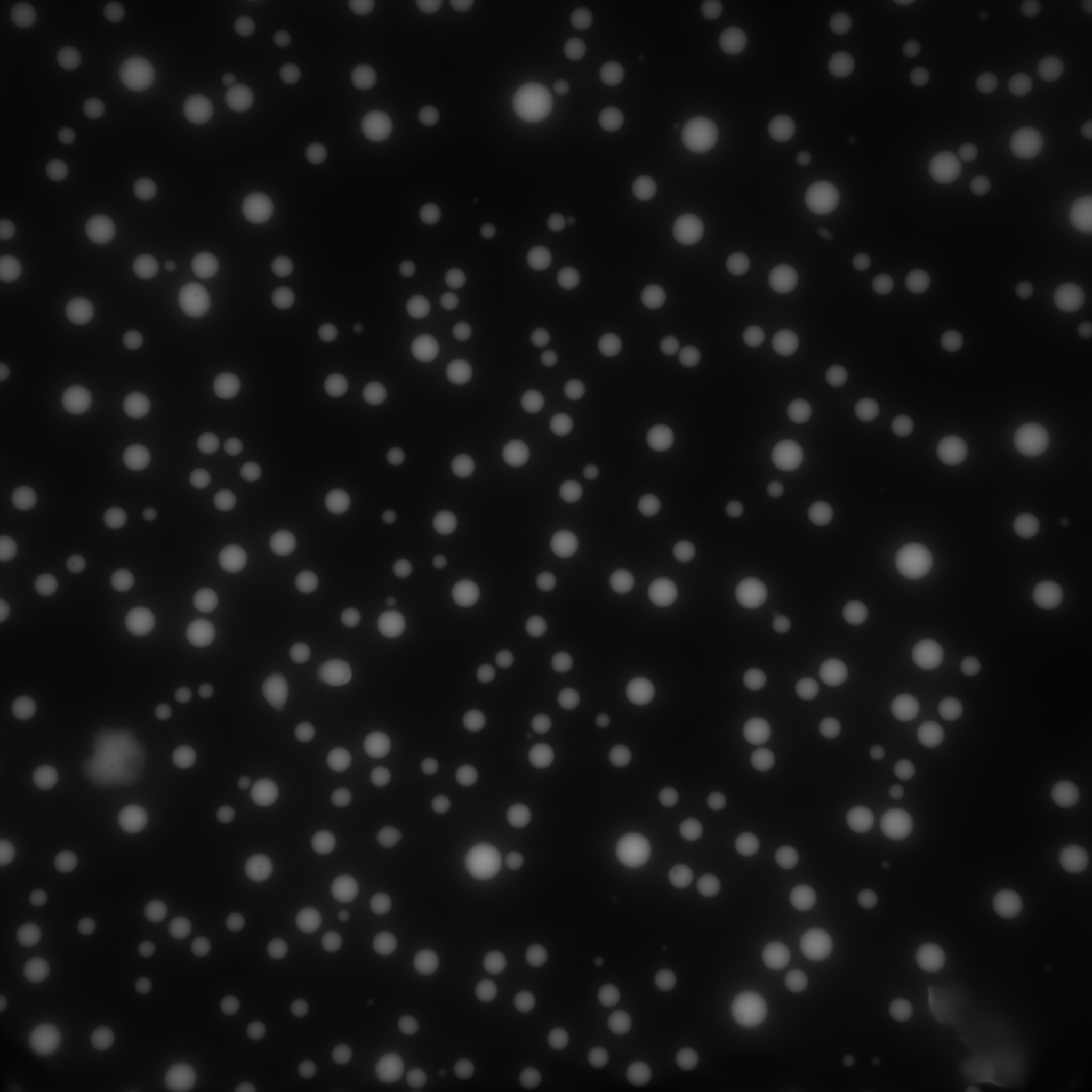

Supplement: Figure 2—source data 1. — Extracted numerical parameters are listed in the accompanying spreadsheet. [file elife-83543-fig2-data1.zip › Figure 2 - source data 1/Figure 2 - source data 1 - inactive ribozyme - Lys19-72 - 1 h.tif]

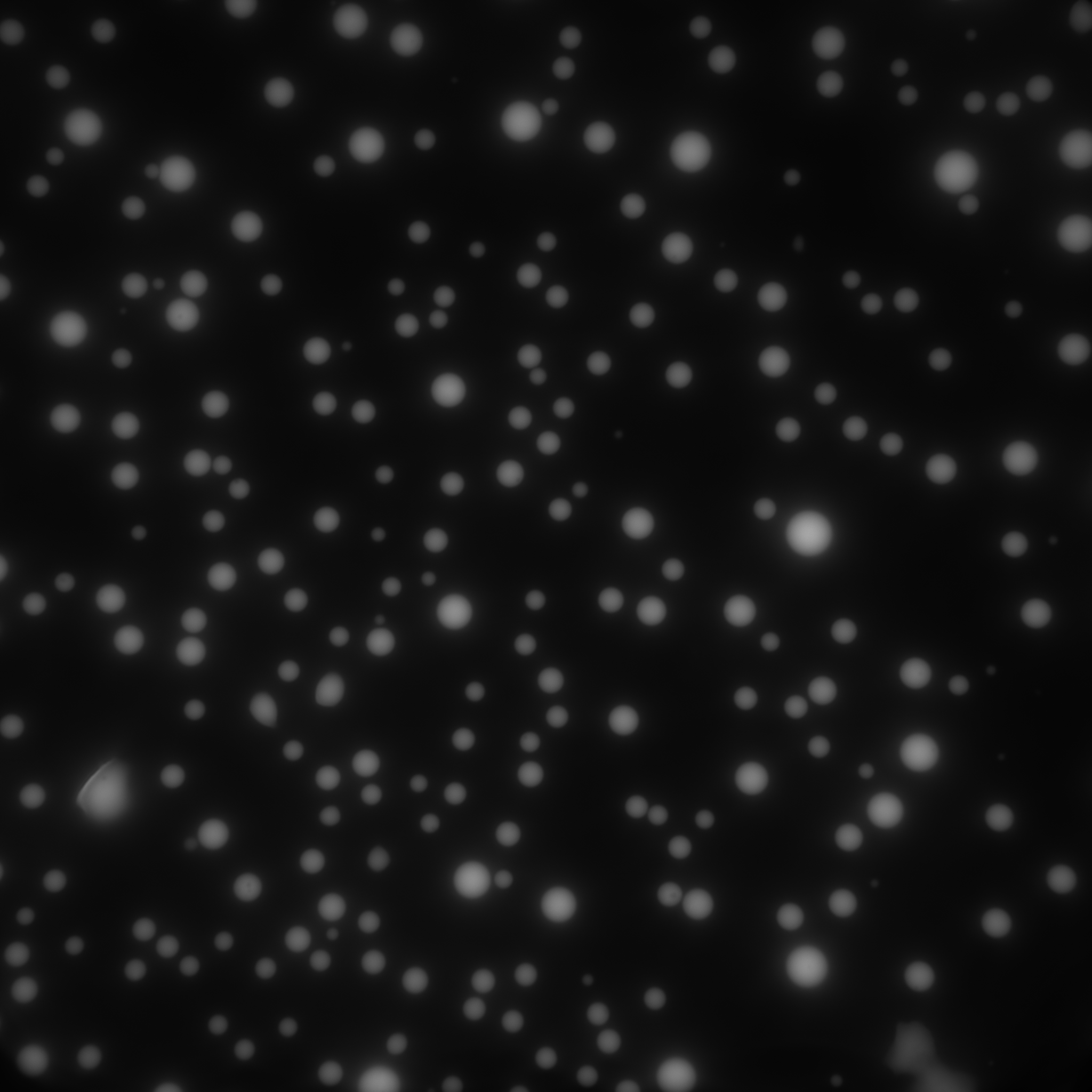

Supplement: Figure 2—source data 1. — Extracted numerical parameters are listed in the accompanying spreadsheet. [file elife-83543-fig2-data1.zip › Figure 2 - source data 1/Figure 2 - source data 1 - inactive ribozyme - Lys19-72 - 2 h.tif]

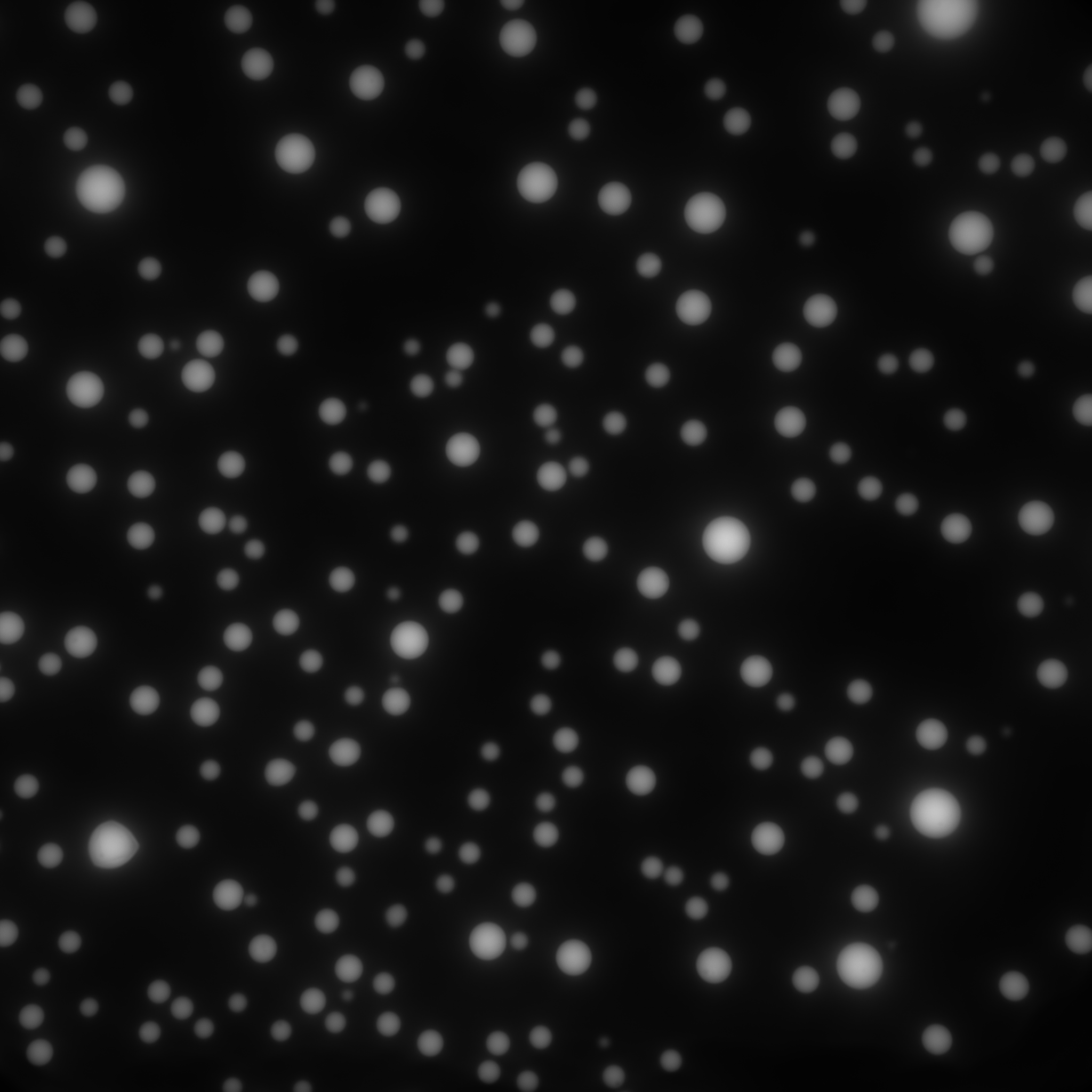

Supplement: Figure 2—source data 1. — Extracted numerical parameters are listed in the accompanying spreadsheet. [file elife-83543-fig2-data1.zip › Figure 2 - source data 1/Figure 2 - source data 1 - inactive ribozyme - Lys19-72 - 24 h.tif]

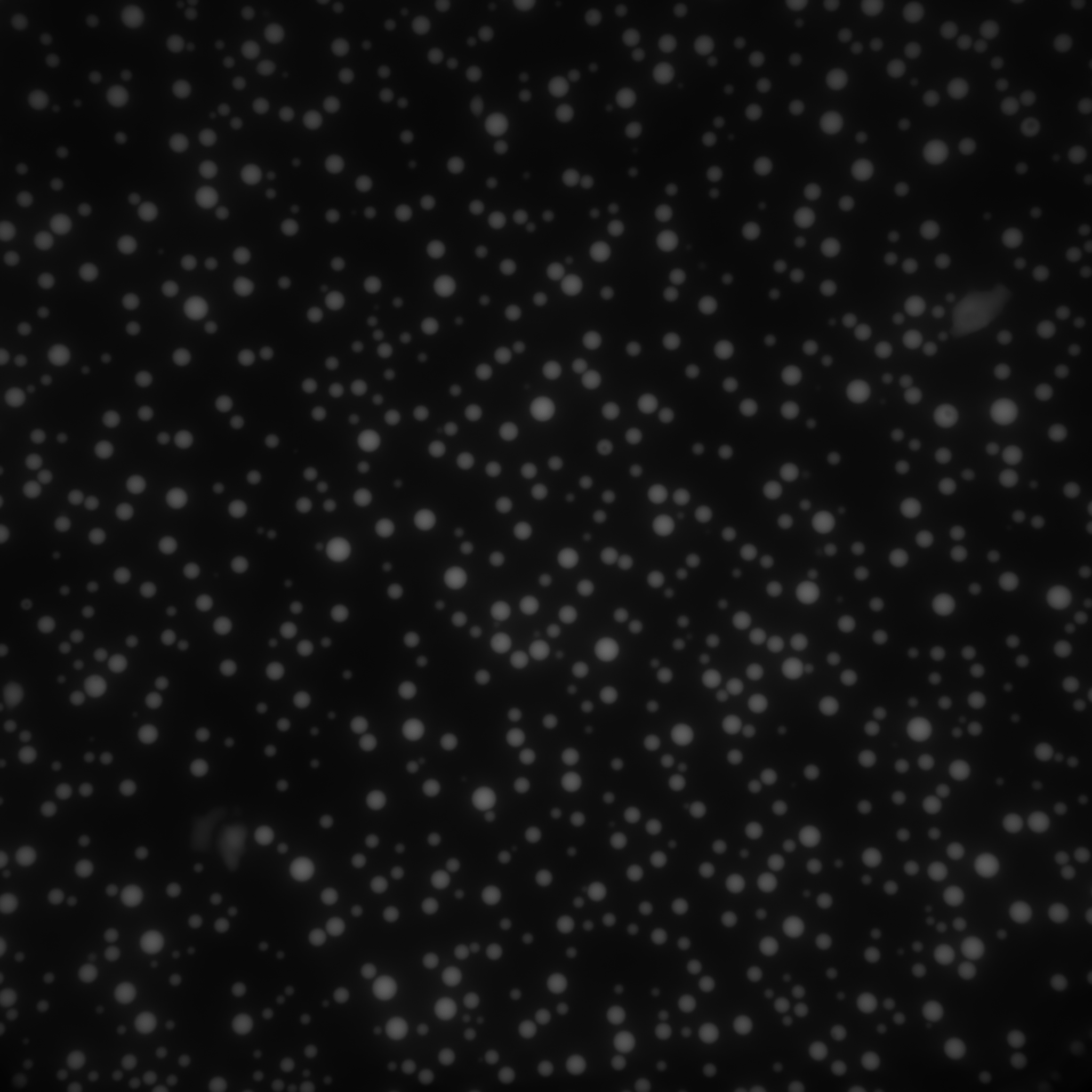

Supplement: Figure 2—source data 2. — Extracted numerical parameters are listed in the accompanying spreadsheet. [file elife-83543-fig2-data2.zip › Figure 2 - source data 2/Figure 2 - source data 2 - active ribozyme - Lys19-72 - surface - 0.5 h.tif]

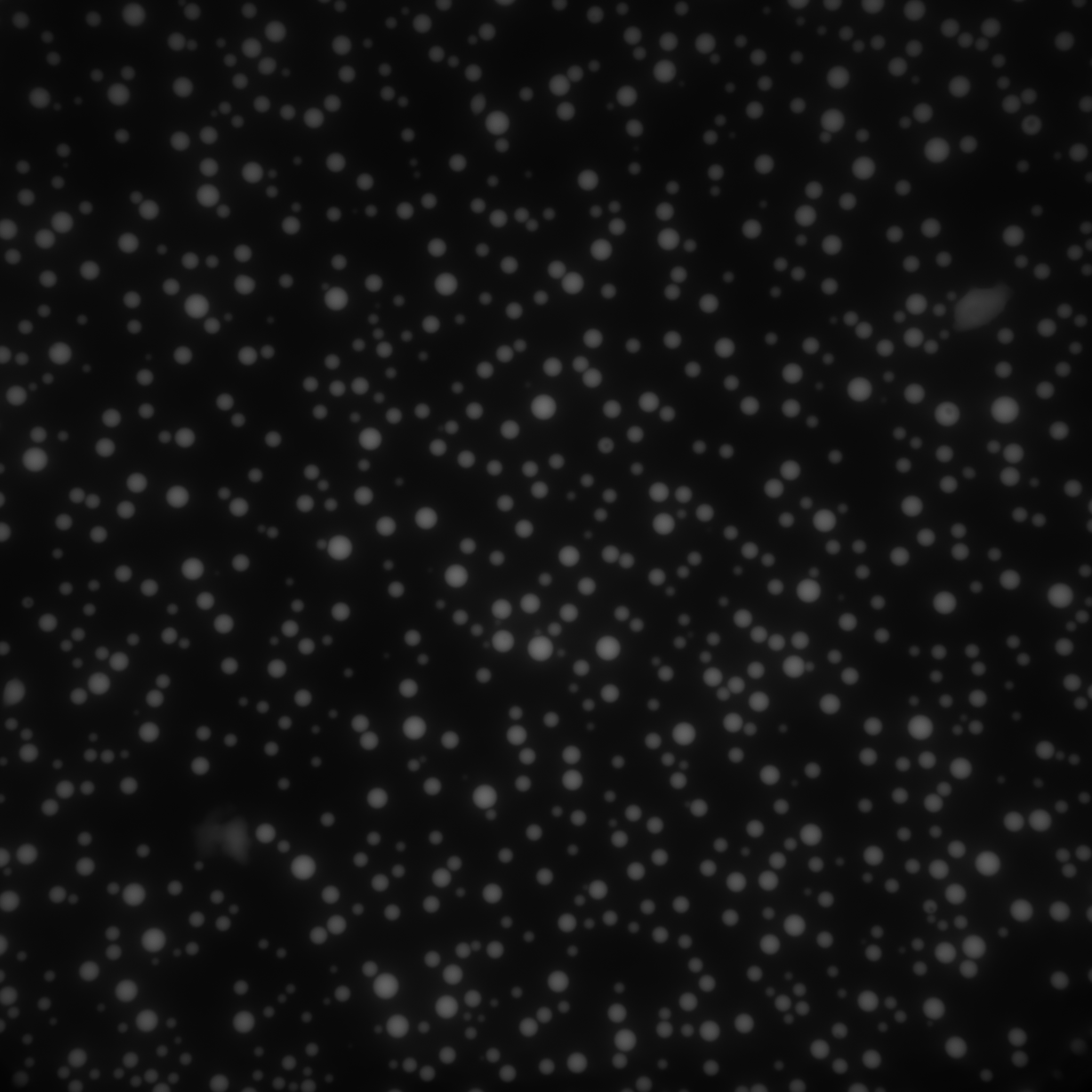

Supplement: Figure 2—source data 2. — Extracted numerical parameters are listed in the accompanying spreadsheet. [file elife-83543-fig2-data2.zip › Figure 2 - source data 2/Figure 2 - source data 2 - active ribozyme - Lys19-72 - surface - 1 h.tif]

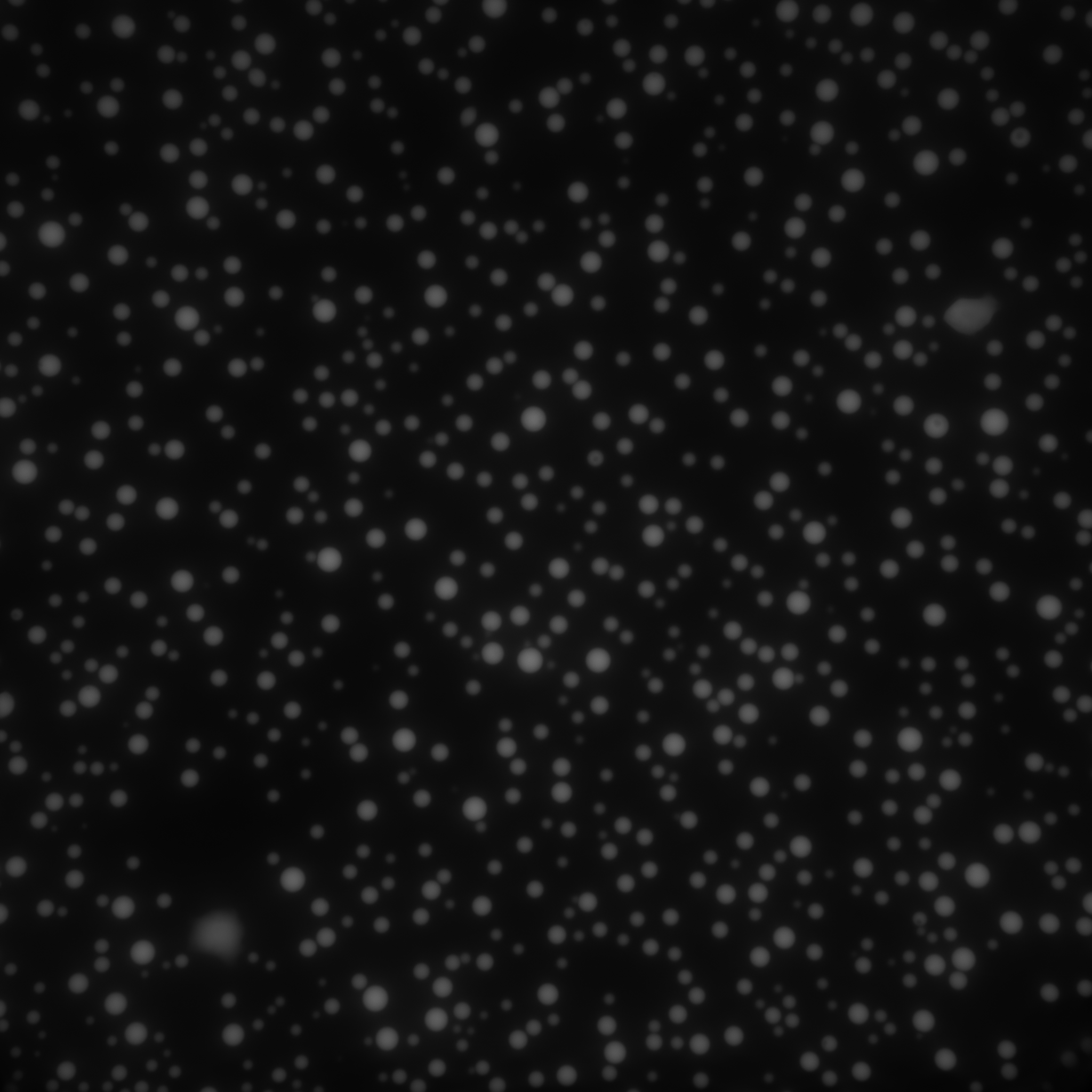

Supplement: Figure 2—source data 2. — Extracted numerical parameters are listed in the accompanying spreadsheet. [file elife-83543-fig2-data2.zip › Figure 2 - source data 2/Figure 2 - source data 2 - active ribozyme - Lys19-72 - surface - 2 h.tif]

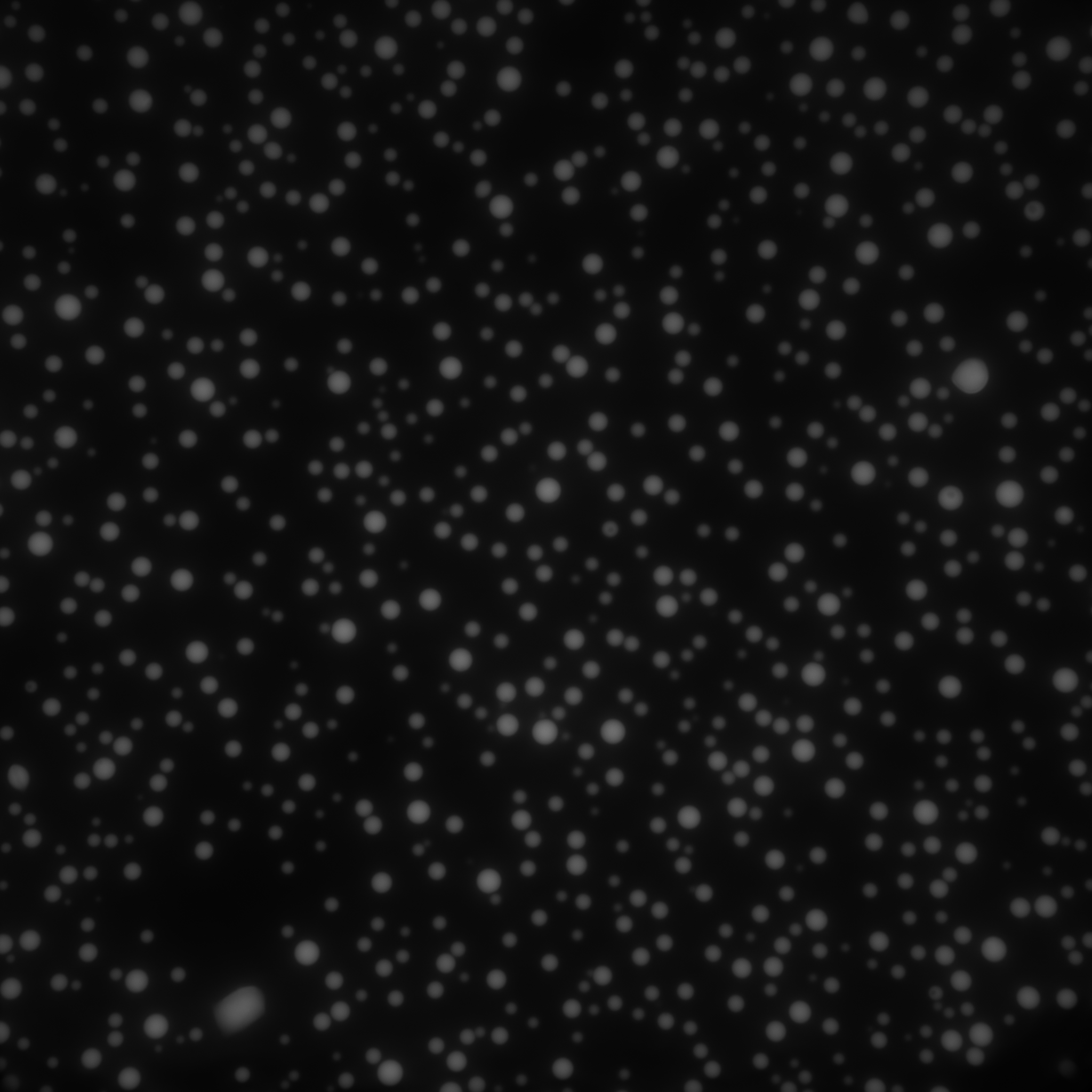

Supplement: Figure 2—source data 2. — Extracted numerical parameters are listed in the accompanying spreadsheet. [file elife-83543-fig2-data2.zip › Figure 2 - source data 2/Figure 2 - source data 2 - active ribozyme - Lys19-72 - surface - 24 h.tif]

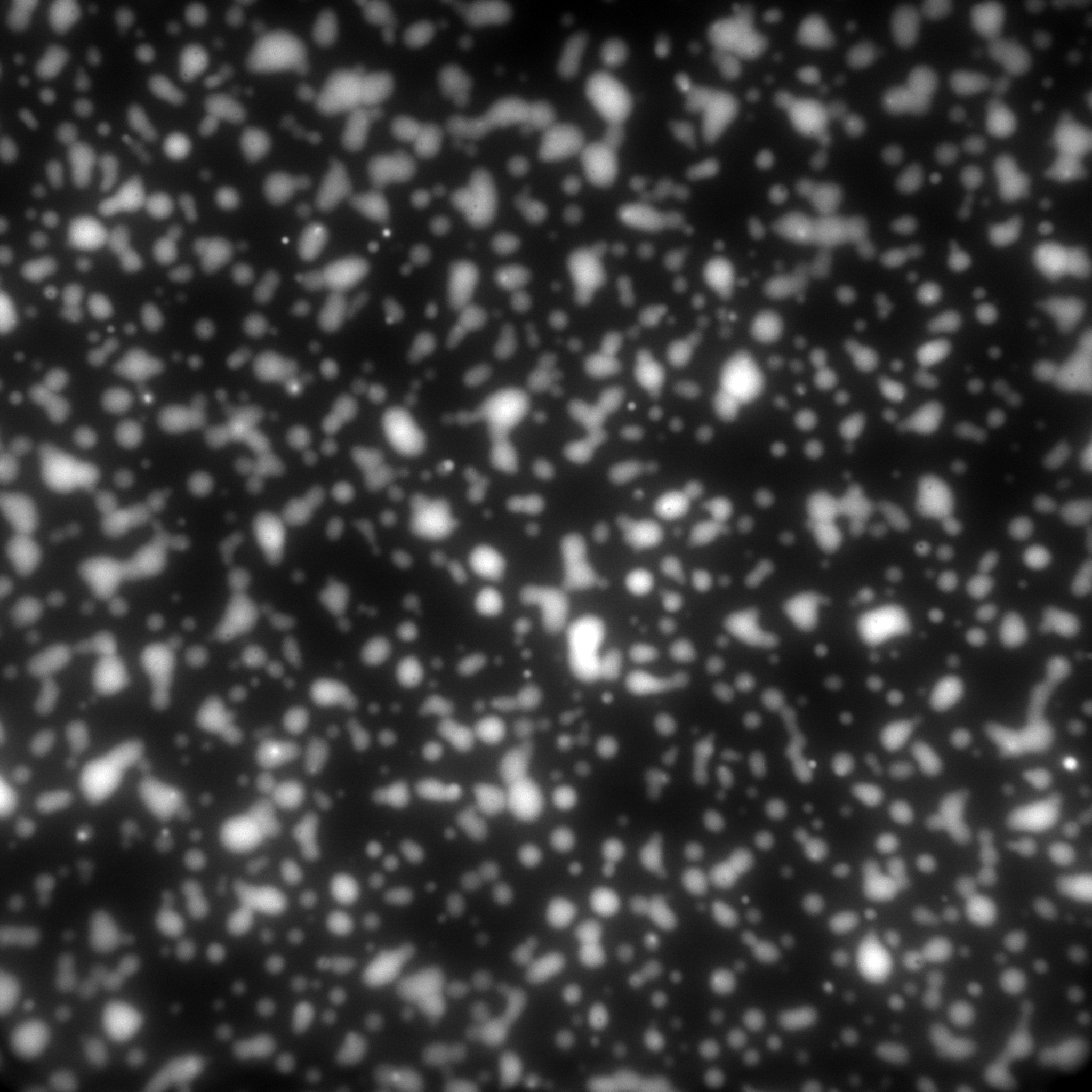

Supplement: Figure 2—source data 2. — Extracted numerical parameters are listed in the accompanying spreadsheet. [file elife-83543-fig2-data2.zip › Figure 2 - source data 2/Figure 2 - source data 2 - inactive ribozyme - Lys19-72 - surface - 0.5 h.tif]

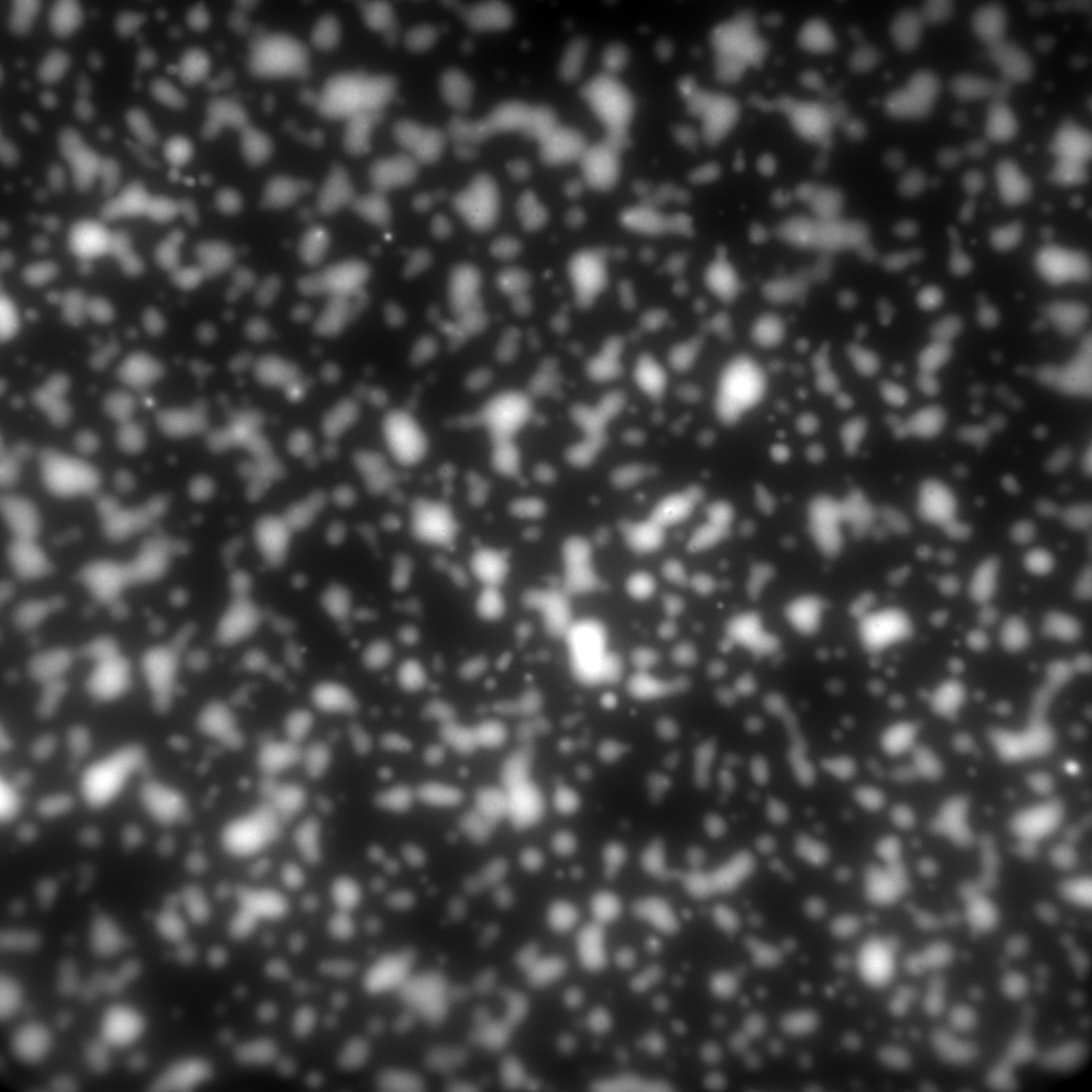

Supplement: Figure 2—source data 2. — Extracted numerical parameters are listed in the accompanying spreadsheet. [file elife-83543-fig2-data2.zip › Figure 2 - source data 2/Figure 2 - source data 2 - inactive ribozyme - Lys19-72 - surface - 1 h.tif]

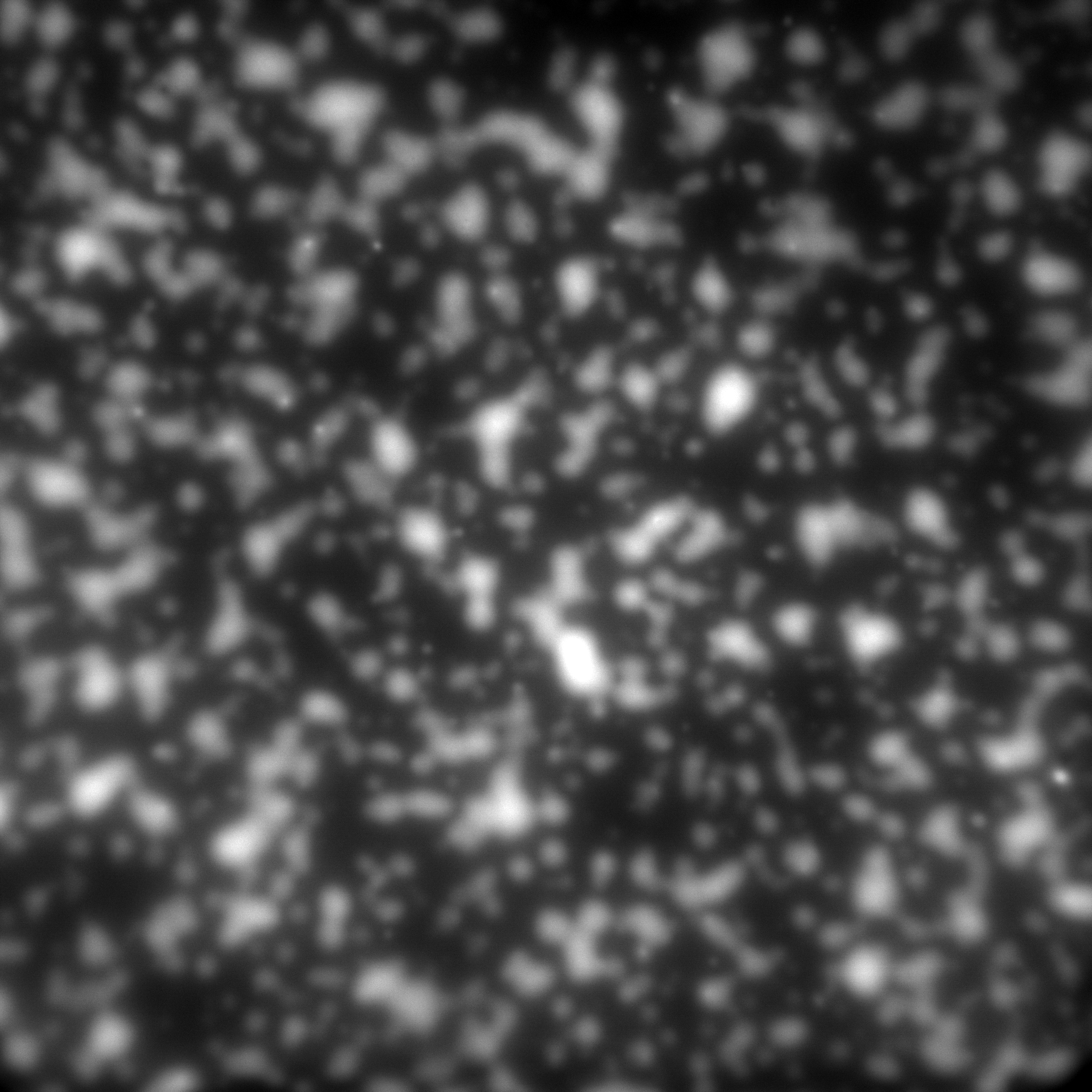

Supplement: Figure 2—source data 2. — Extracted numerical parameters are listed in the accompanying spreadsheet. [file elife-83543-fig2-data2.zip › Figure 2 - source data 2/Figure 2 - source data 2 - inactive ribozyme - Lys19-72 - surface - 2 h.tif]

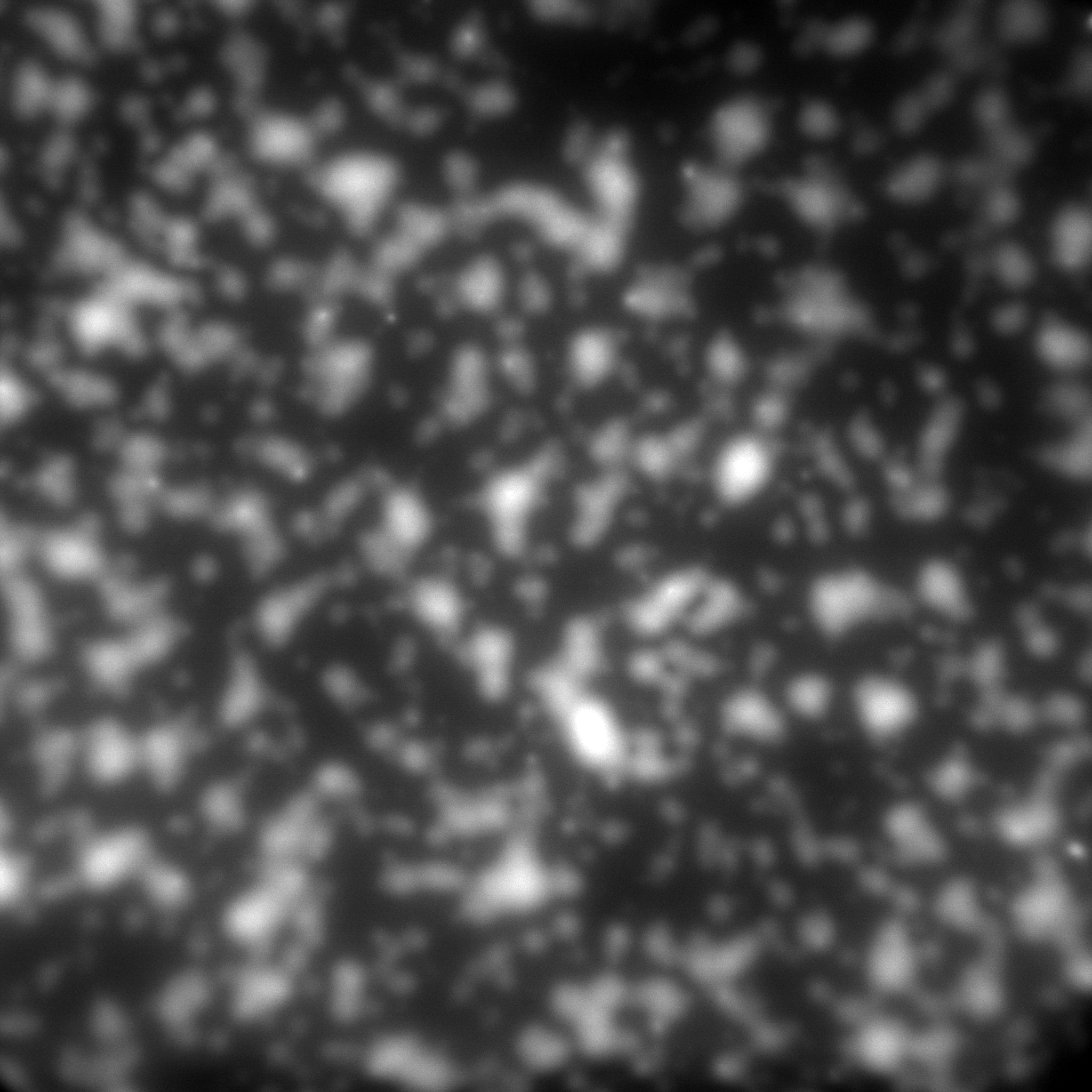

Supplement: Figure 2—source data 2. — Extracted numerical parameters are listed in the accompanying spreadsheet. [file elife-83543-fig2-data2.zip › Figure 2 - source data 2/Figure 2 - source data 2 - inactive ribozyme - Lys19-72 - surface - 24 h.tif]

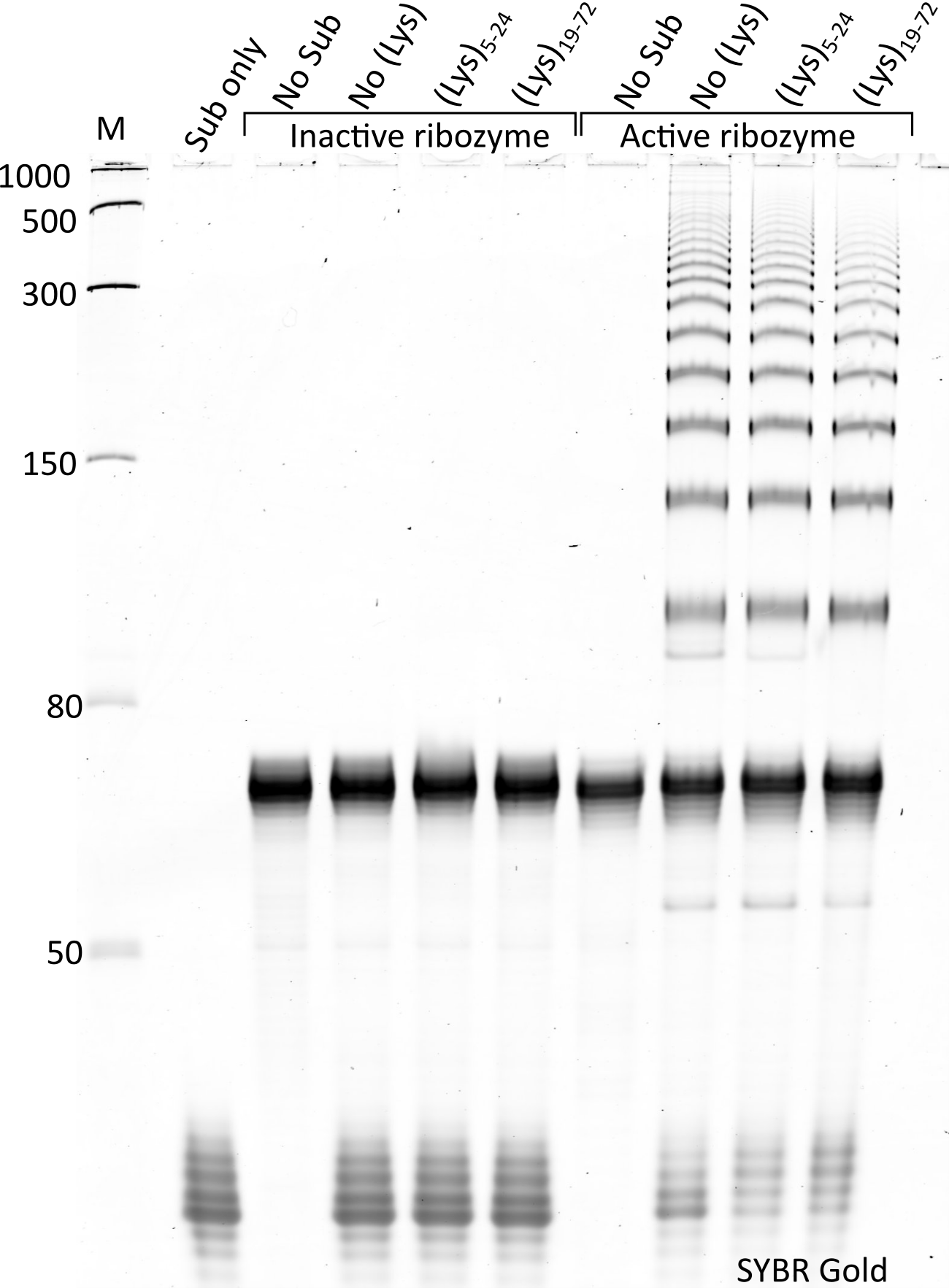

Supplement: Figure 2—figure supplement 1—source data 1. [file elife-83543-fig2-figsupp1-data1.zip › Figure 2 - supplement 1 - source data 1/Figure 2 - supplement 1 - source data 1 - labelled gel.png]

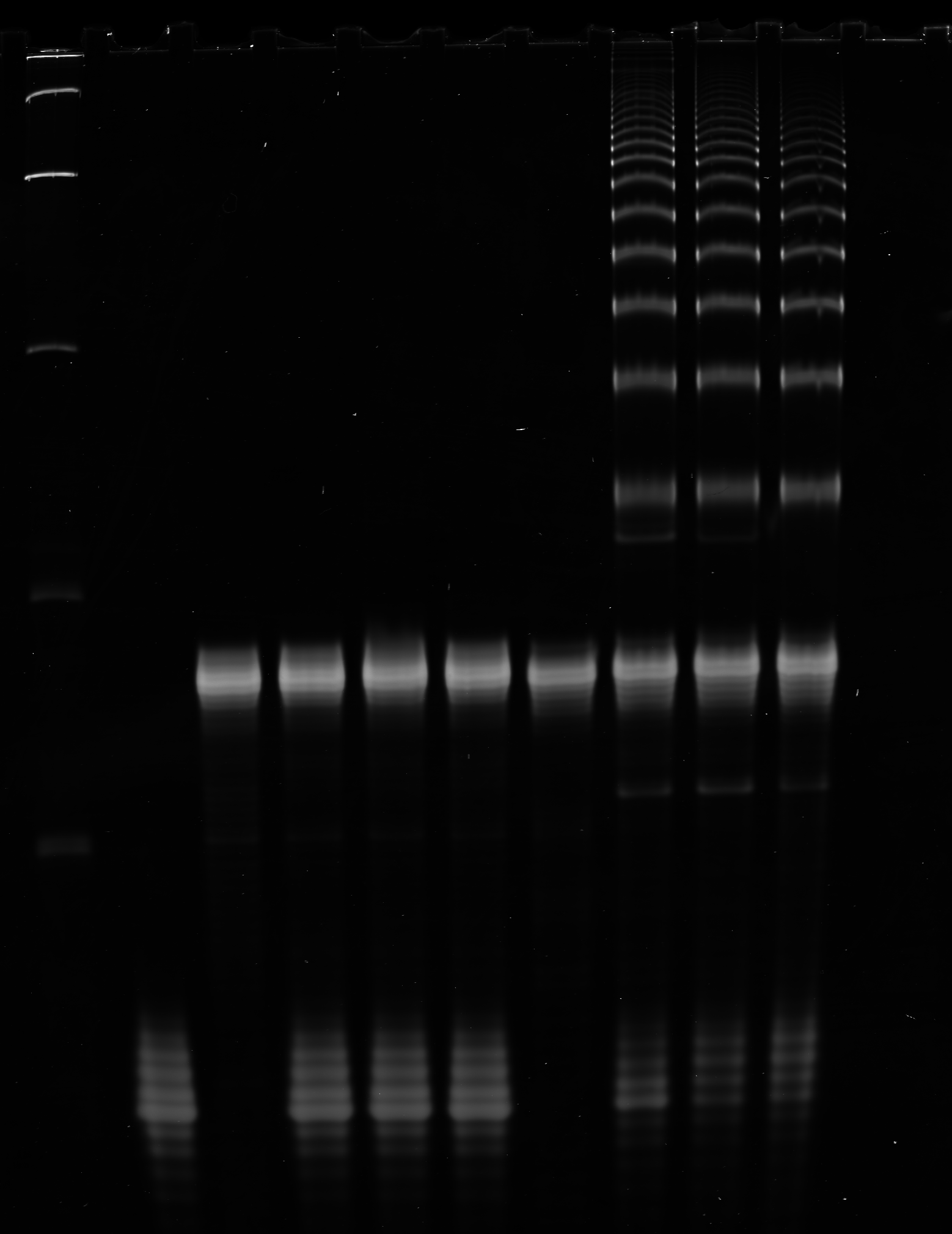

Supplement: Figure 2—figure supplement 1—source data 1. [file elife-83543-fig2-figsupp1-data1.zip › Figure 2 - supplement 1 - source data 1/Figure 2 - supplement 1 - source data 1 - raw gel.tif]

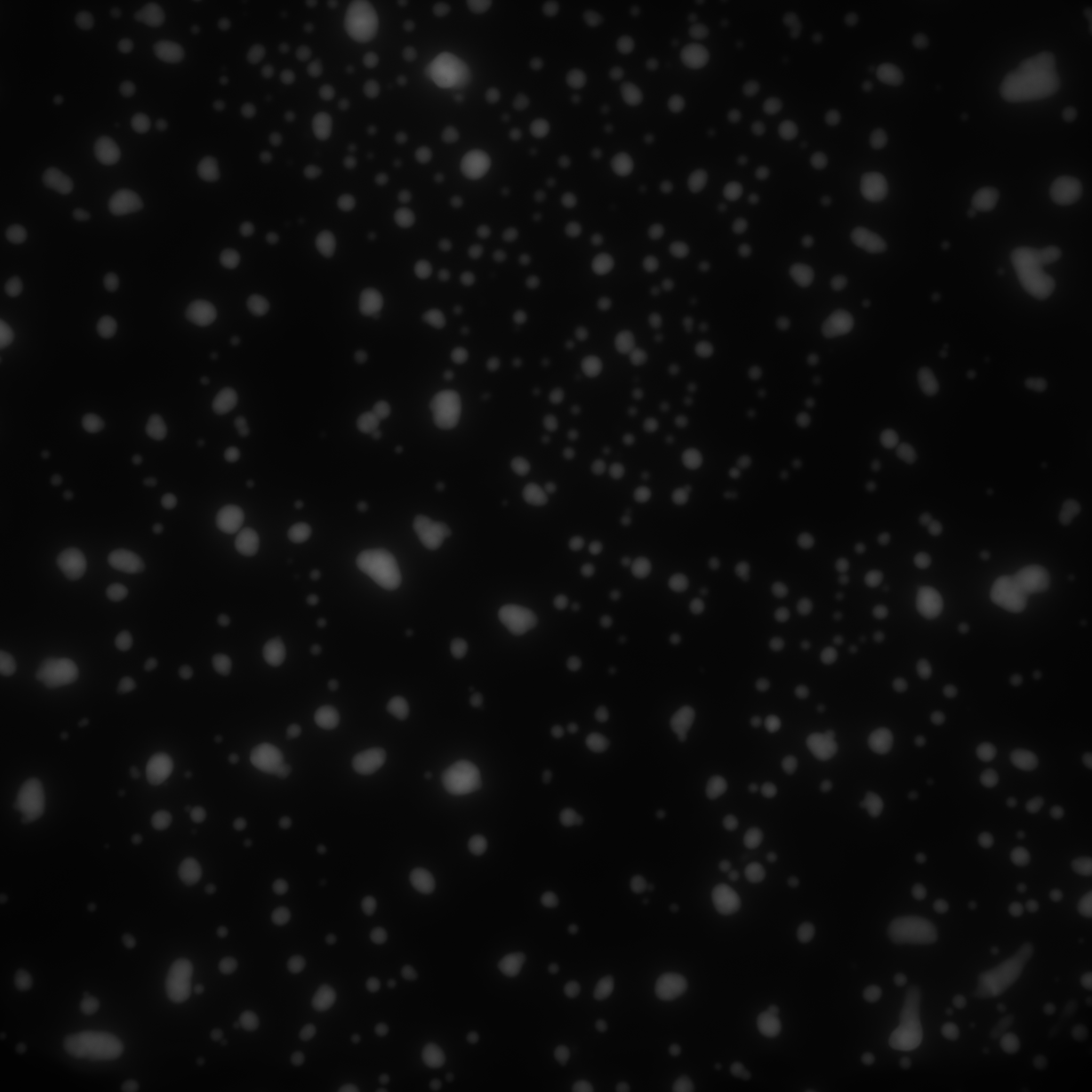

Supplement: Figure 2—figure supplement 2—source data 1. — Extracted numerical parameters are listed in the accompanying spreadsheet. [file elife-83543-fig2-figsupp2-data1.zip › Figure 2 - supplement 2-4 - source data 1/Figure 2 - supplement 2 - source data 1 - active ribozyme - Lys5-24 - 0.5 h.tif]

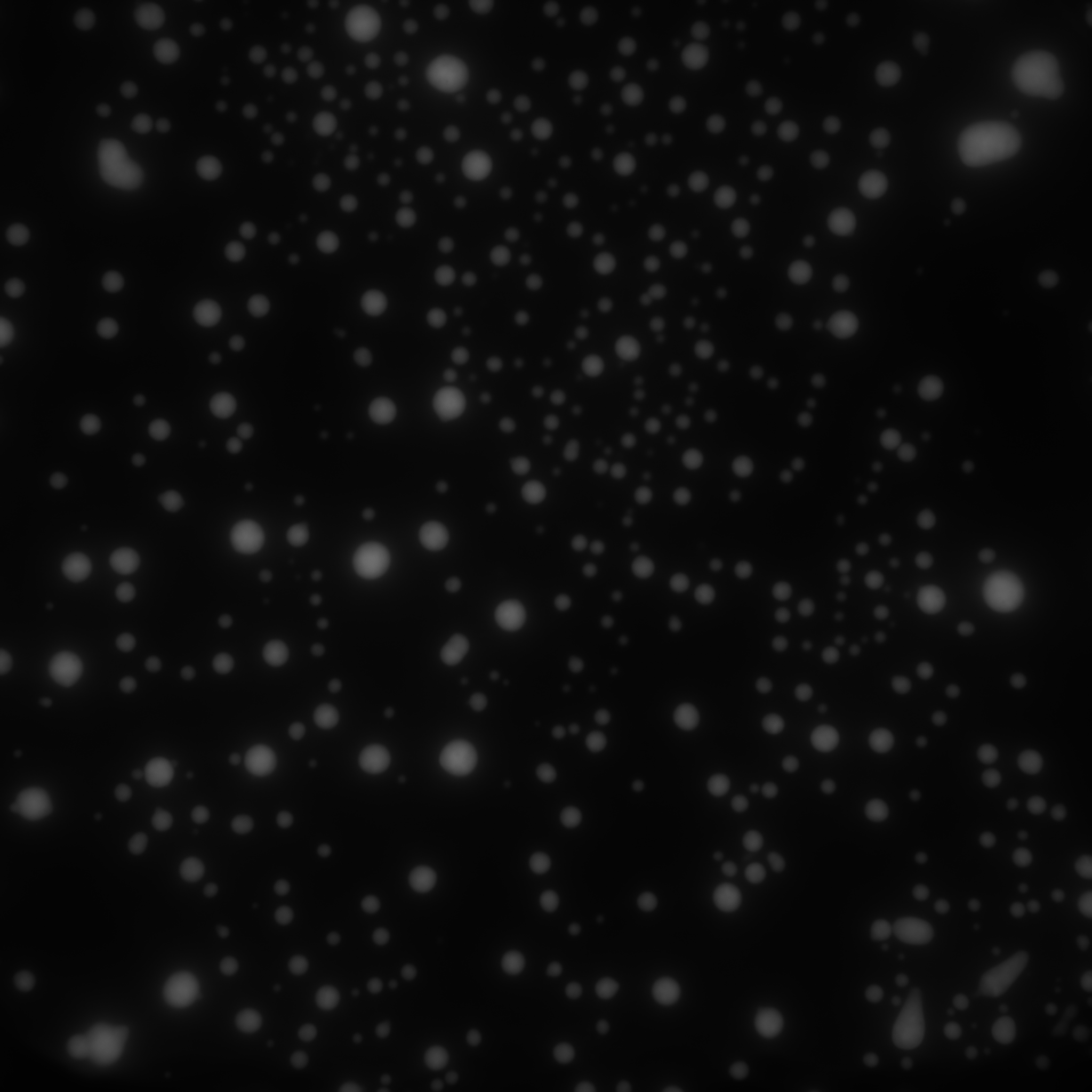

Supplement: Figure 2—figure supplement 2—source data 1. — Extracted numerical parameters are listed in the accompanying spreadsheet. [file elife-83543-fig2-figsupp2-data1.zip › Figure 2 - supplement 2-4 - source data 1/Figure 2 - supplement 2 - source data 1 - active ribozyme - Lys5-24 - 1 h.tif]

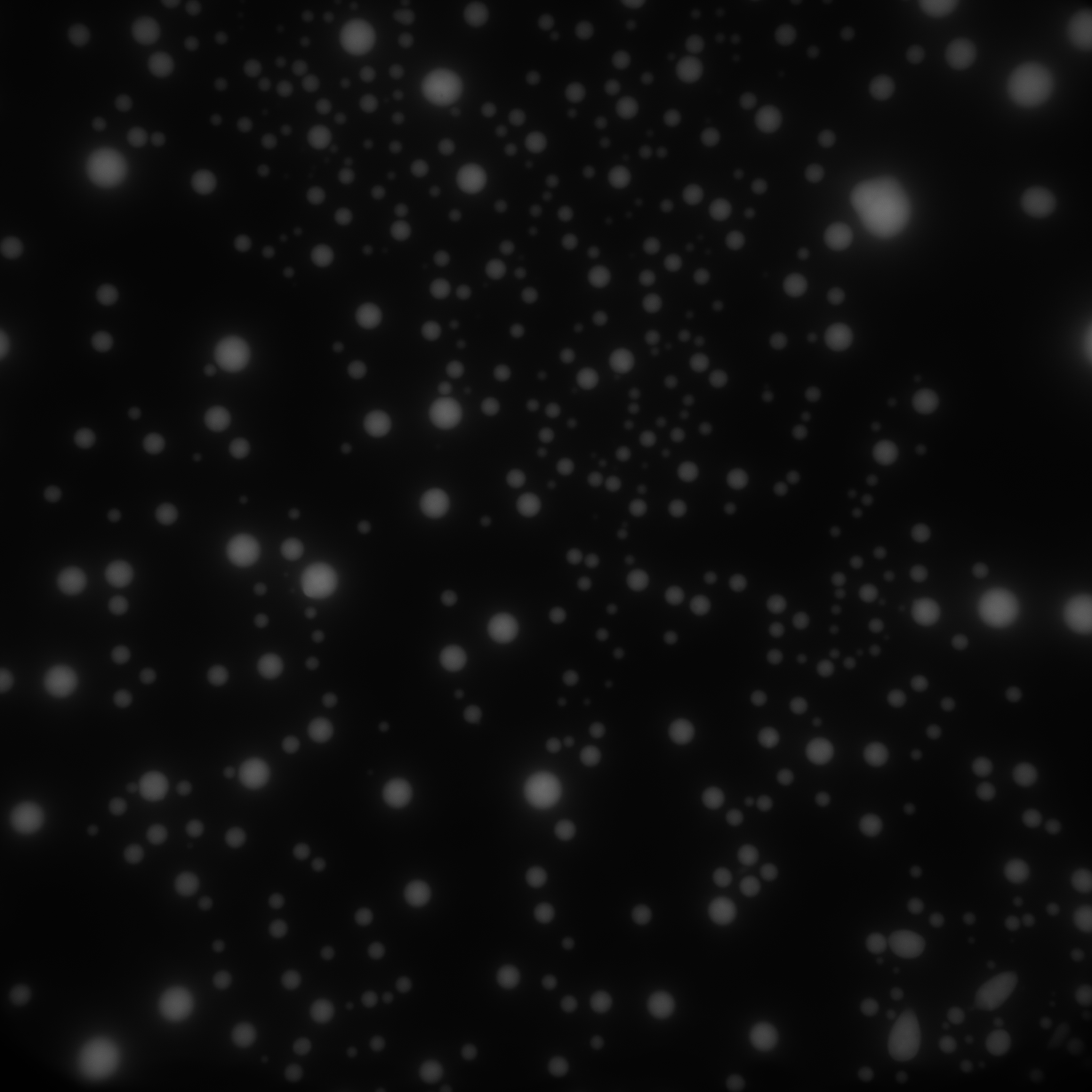

Supplement: Figure 2—figure supplement 2—source data 1. — Extracted numerical parameters are listed in the accompanying spreadsheet. [file elife-83543-fig2-figsupp2-data1.zip › Figure 2 - supplement 2-4 - source data 1/Figure 2 - supplement 2 - source data 1 - active ribozyme - Lys5-24 - 2 h.tif]

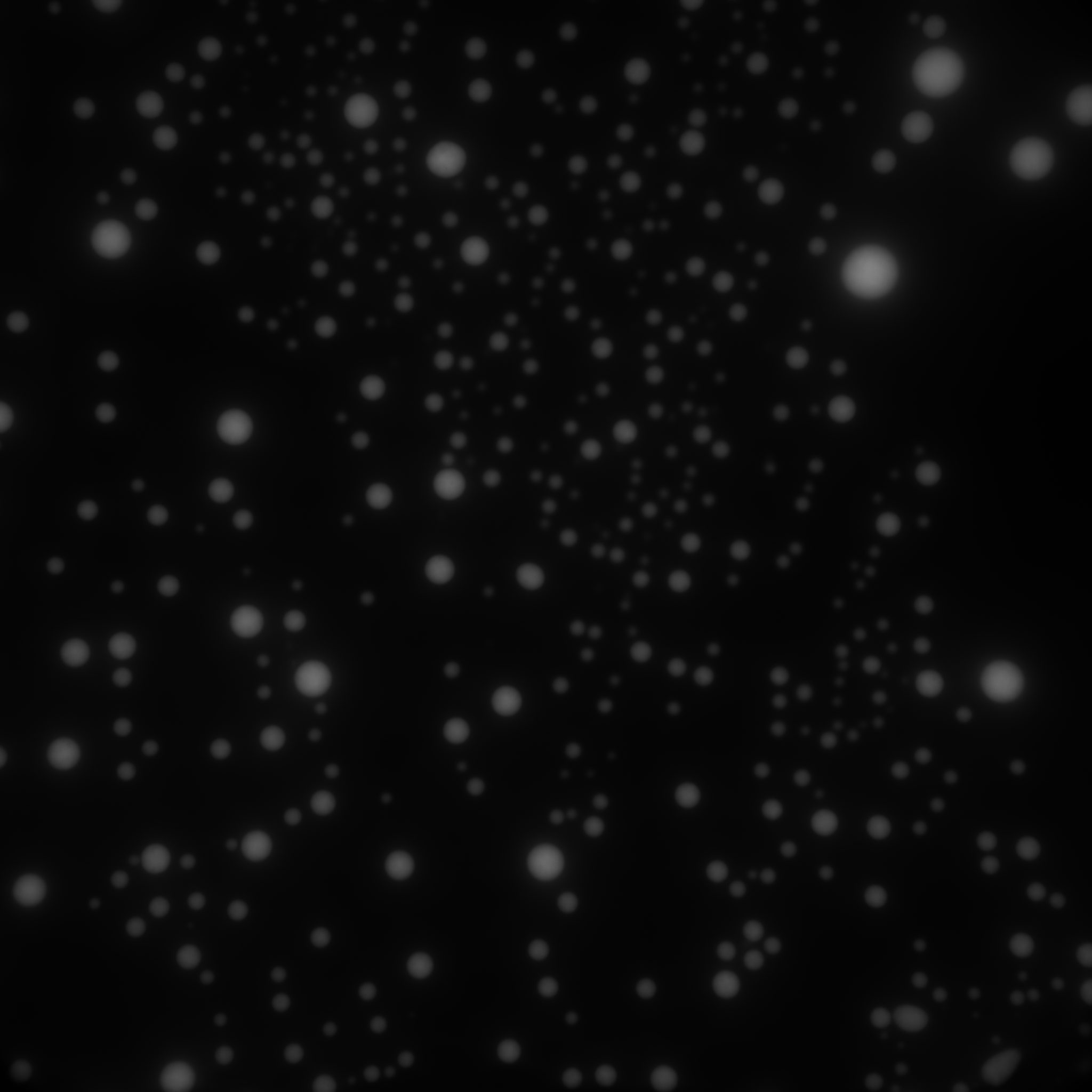

Supplement: Figure 2—figure supplement 2—source data 1. — Extracted numerical parameters are listed in the accompanying spreadsheet. [file elife-83543-fig2-figsupp2-data1.zip › Figure 2 - supplement 2-4 - source data 1/Figure 2 - supplement 2 - source data 1 - active ribozyme - Lys5-24 - 24 h.tif]

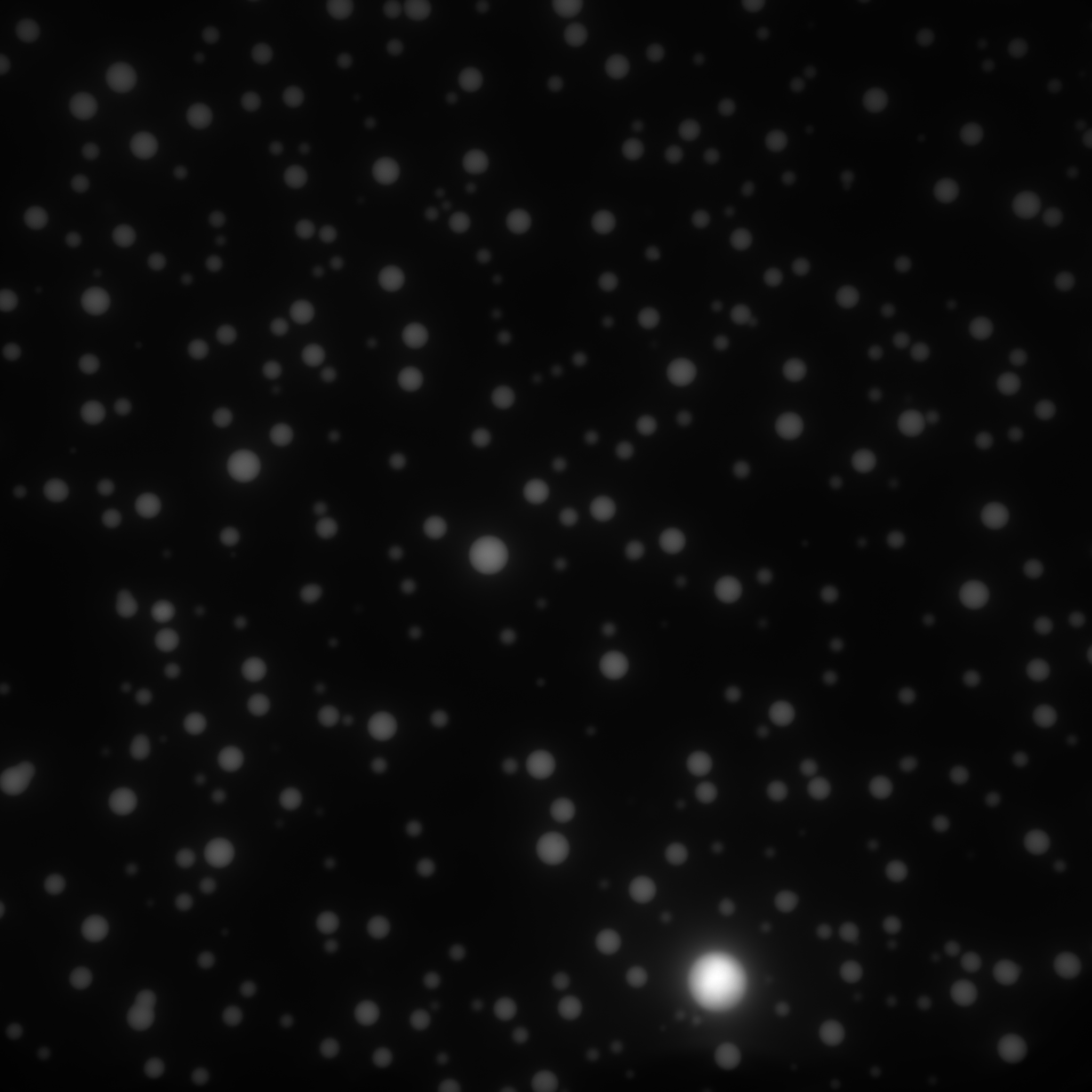

Supplement: Figure 2—figure supplement 2—source data 1. — Extracted numerical parameters are listed in the accompanying spreadsheet. [file elife-83543-fig2-figsupp2-data1.zip › Figure 2 - supplement 2-4 - source data 1/Figure 2 - supplement 2 - source data 1 - inactive ribozyme - Lys5-24 - 0.5 h.tif]

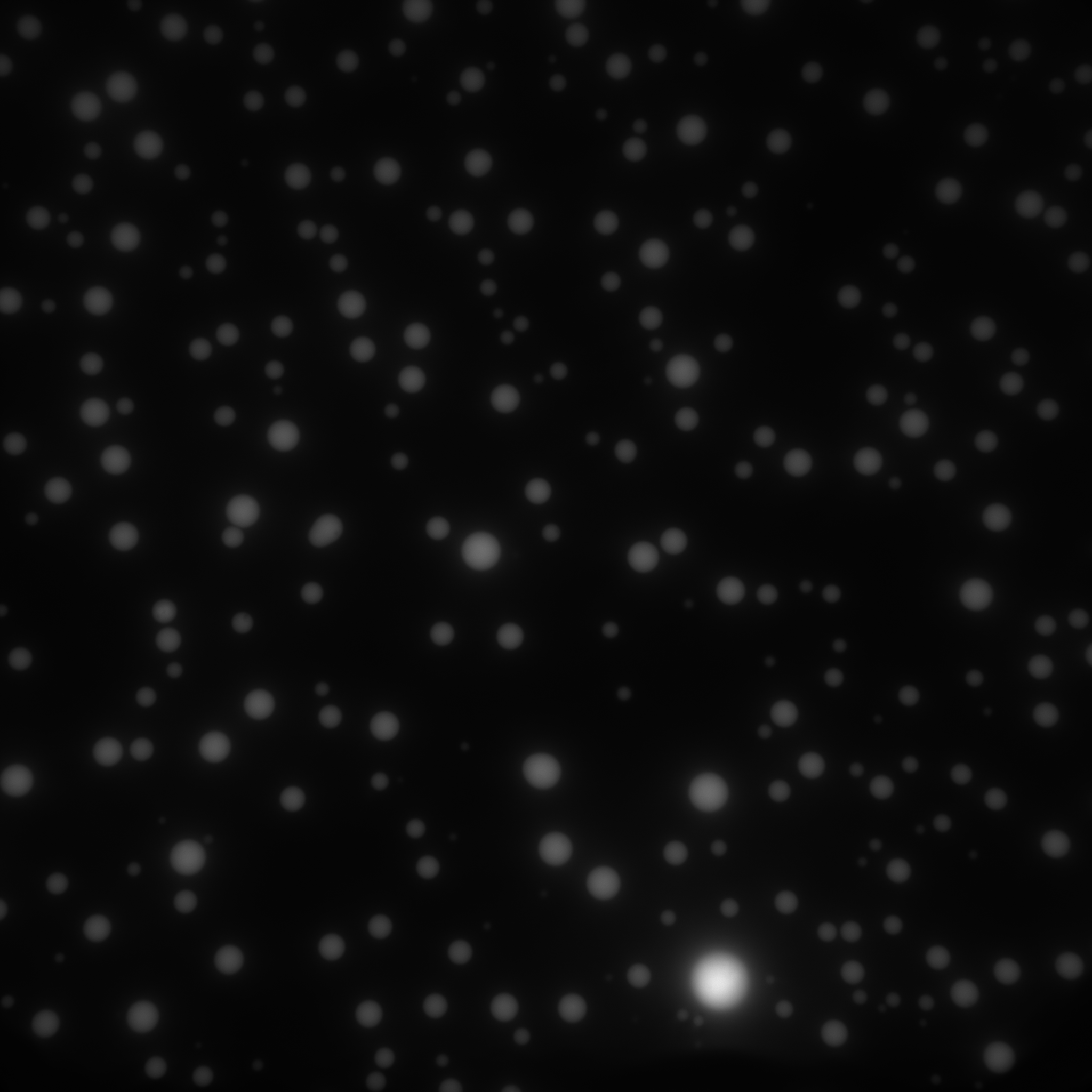

Supplement: Figure 2—figure supplement 2—source data 1. — Extracted numerical parameters are listed in the accompanying spreadsheet. [file elife-83543-fig2-figsupp2-data1.zip › Figure 2 - supplement 2-4 - source data 1/Figure 2 - supplement 2 - source data 1 - inactive ribozyme - Lys5-24 - 1 h.tif]

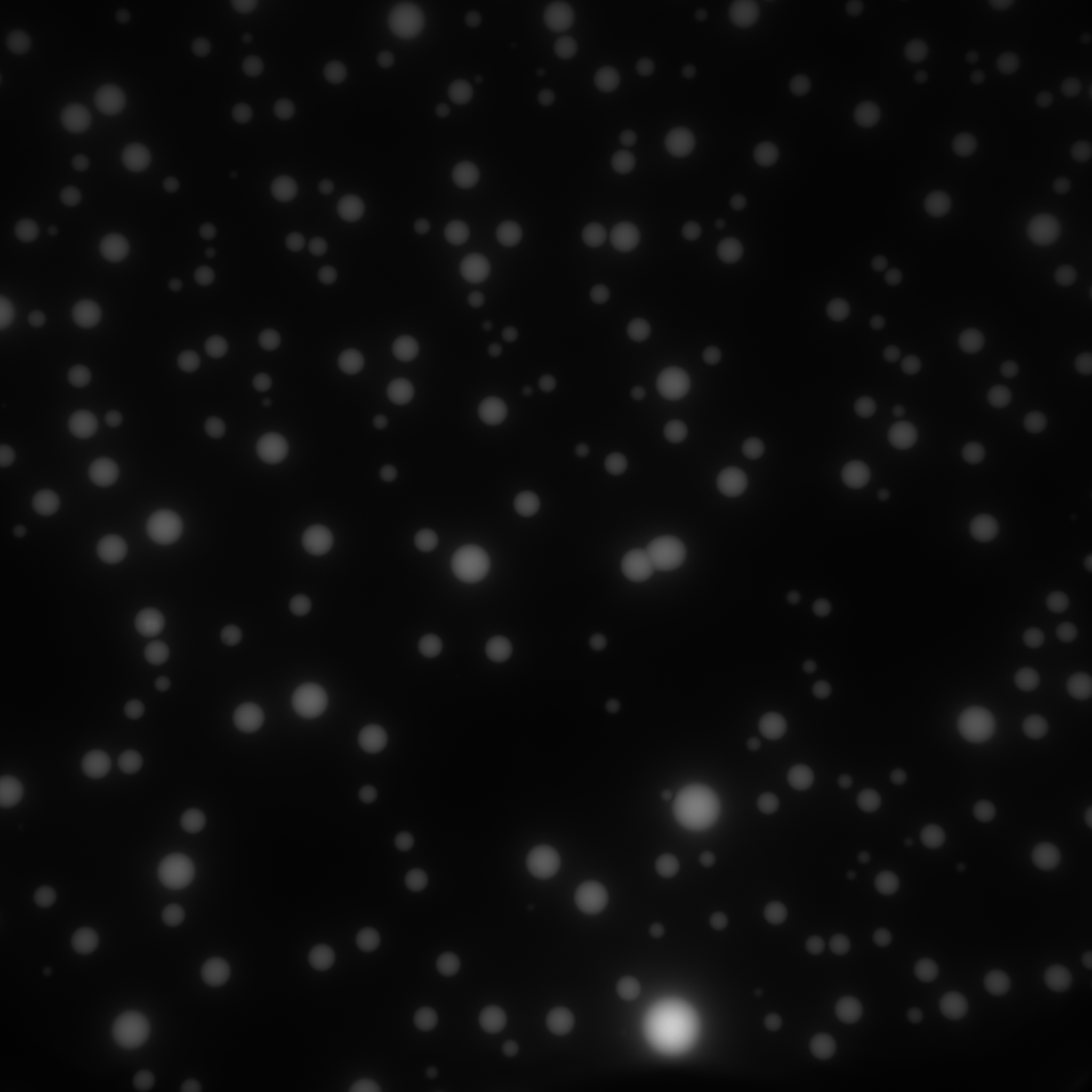

Supplement: Figure 2—figure supplement 2—source data 1. — Extracted numerical parameters are listed in the accompanying spreadsheet. [file elife-83543-fig2-figsupp2-data1.zip › Figure 2 - supplement 2-4 - source data 1/Figure 2 - supplement 2 - source data 1 - inactive ribozyme - Lys5-24 - 2 h.tif]

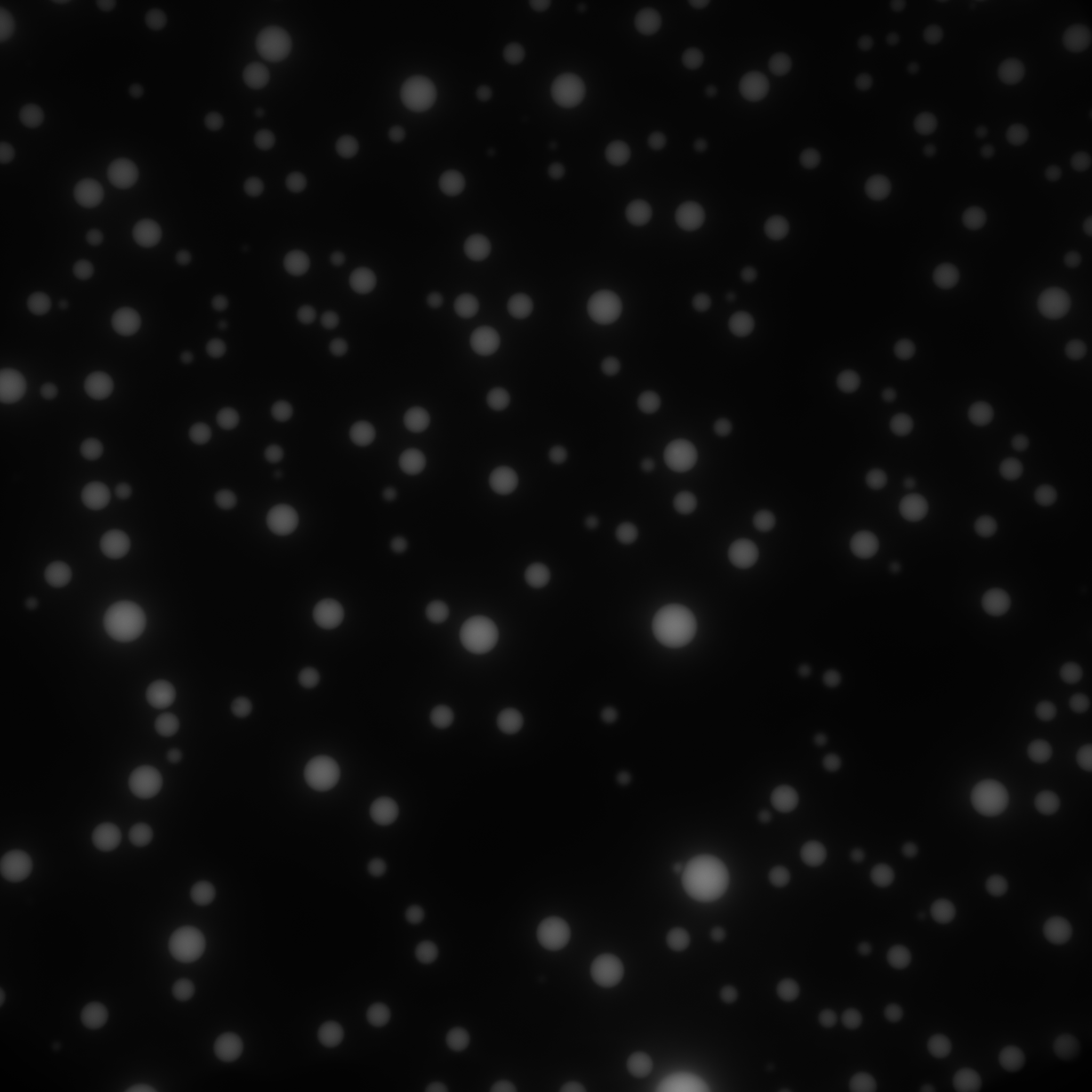

Supplement: Figure 2—figure supplement 2—source data 1. — Extracted numerical parameters are listed in the accompanying spreadsheet. [file elife-83543-fig2-figsupp2-data1.zip › Figure 2 - supplement 2-4 - source data 1/Figure 2 - supplement 2 - source data 1 - inactive ribozyme - Lys5-24 - 24 h.tif]

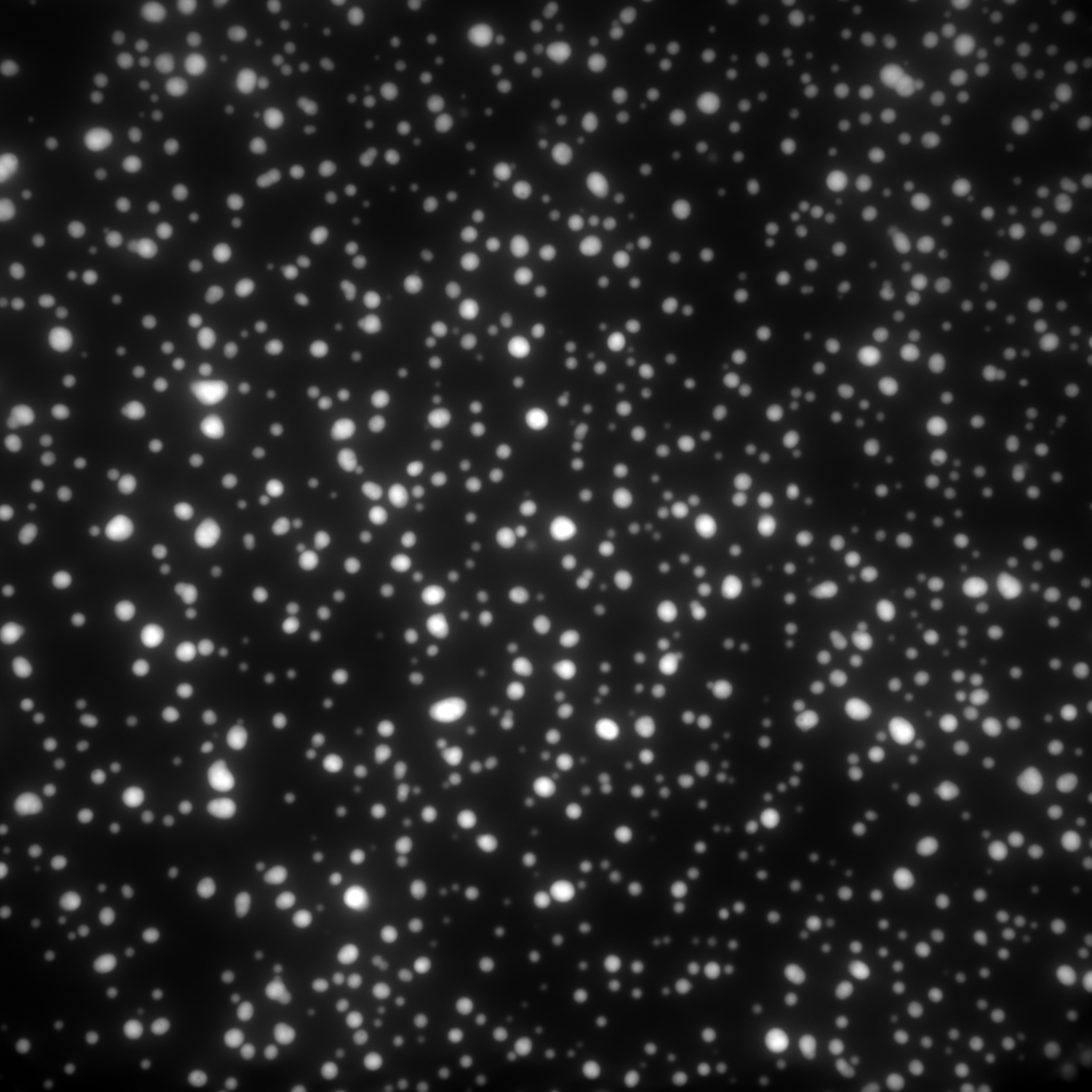

Supplement: Figure 2—figure supplement 2—source data 2. — Extracted numerical parameters are listed in the accompanying spreadsheet. [file elife-83543-fig2-figsupp2-data2.zip › Figure 2 - supplement 2 - source data 2/Figure 2 - supplement 2 - source data 2 - active ribozyme - Lys5-24 - surface - 0.5 h.tif]

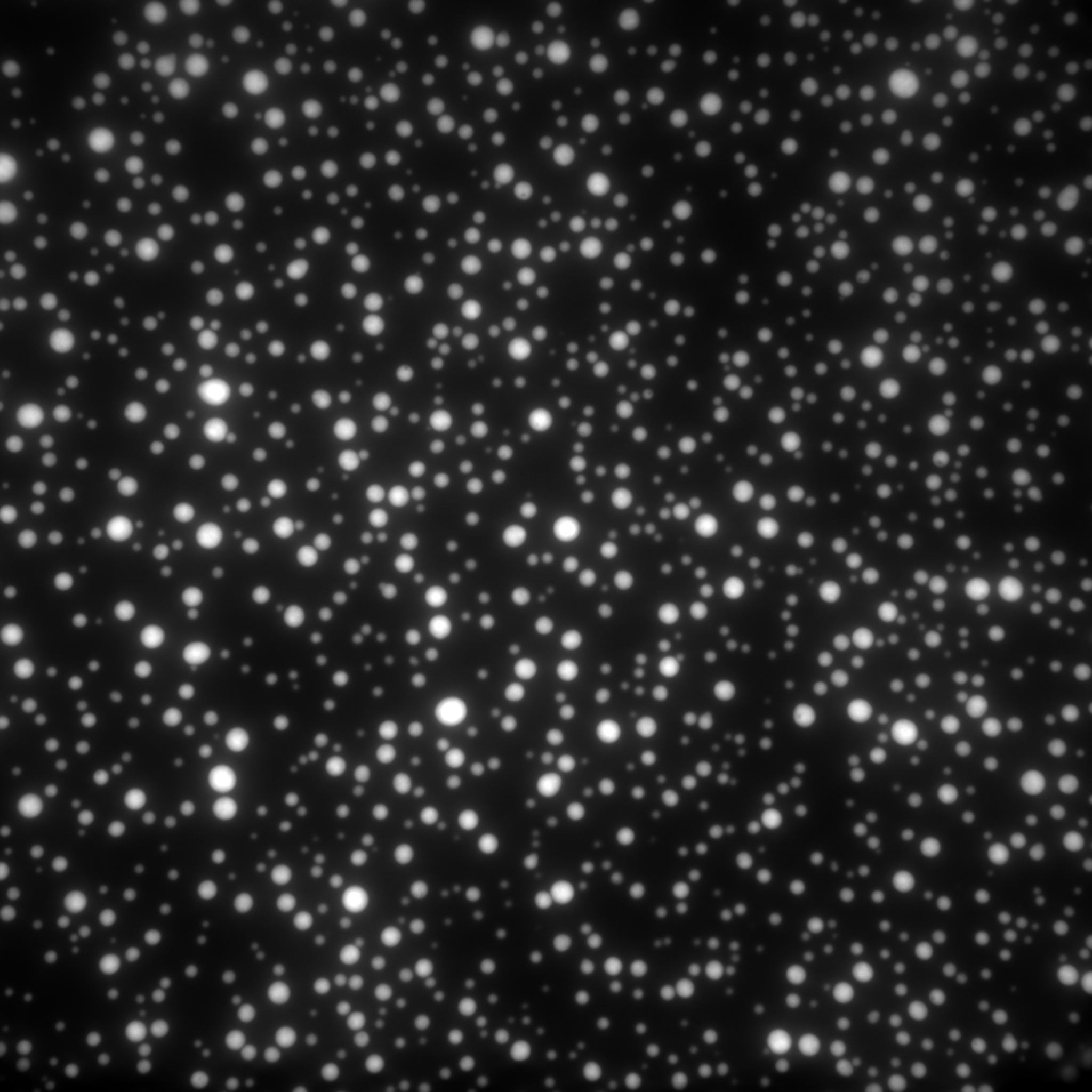

Supplement: Figure 2—figure supplement 2—source data 2. — Extracted numerical parameters are listed in the accompanying spreadsheet. [file elife-83543-fig2-figsupp2-data2.zip › Figure 2 - supplement 2 - source data 2/Figure 2 - supplement 2 - source data 2 - active ribozyme - Lys5-24 - surface - 1 h.tif]

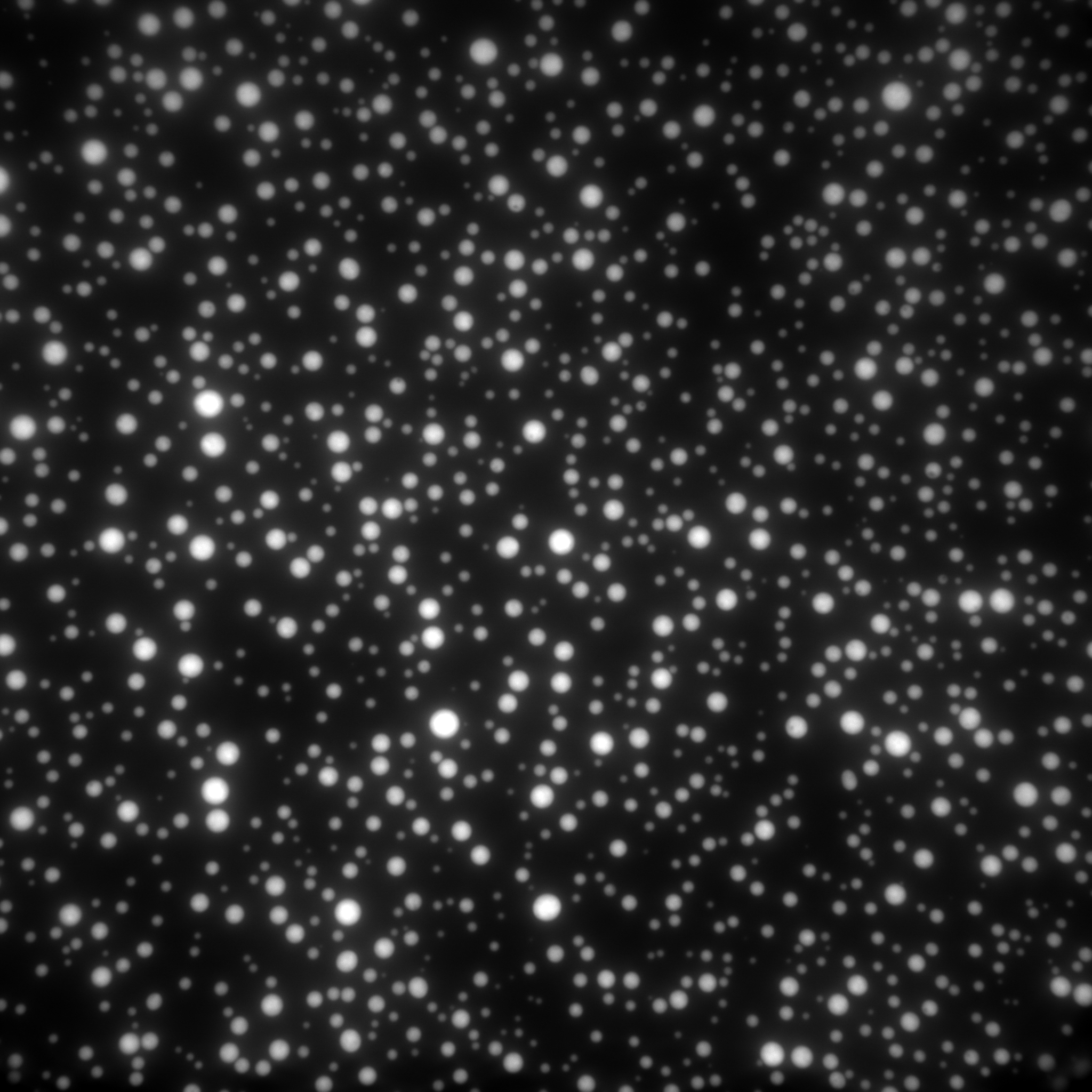

Supplement: Figure 2—figure supplement 2—source data 2. — Extracted numerical parameters are listed in the accompanying spreadsheet. [file elife-83543-fig2-figsupp2-data2.zip › Figure 2 - supplement 2 - source data 2/Figure 2 - supplement 2 - source data 2 - active ribozyme - Lys5-24 - surface - 2 h.tif]

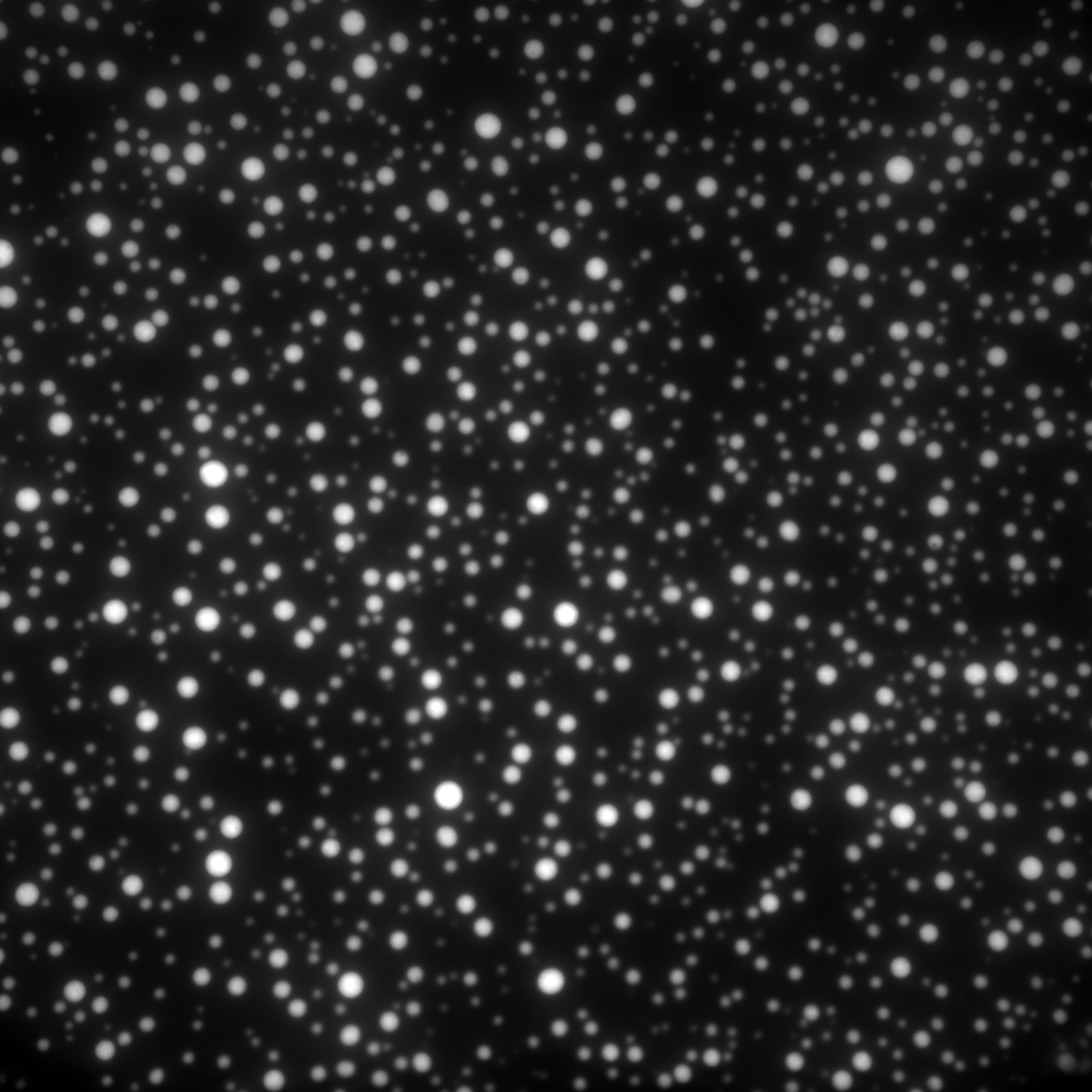

Supplement: Figure 2—figure supplement 2—source data 2. — Extracted numerical parameters are listed in the accompanying spreadsheet. [file elife-83543-fig2-figsupp2-data2.zip › Figure 2 - supplement 2 - source data 2/Figure 2 - supplement 2 - source data 2 - active ribozyme - Lys5-24 - surface - 24 h.tif]

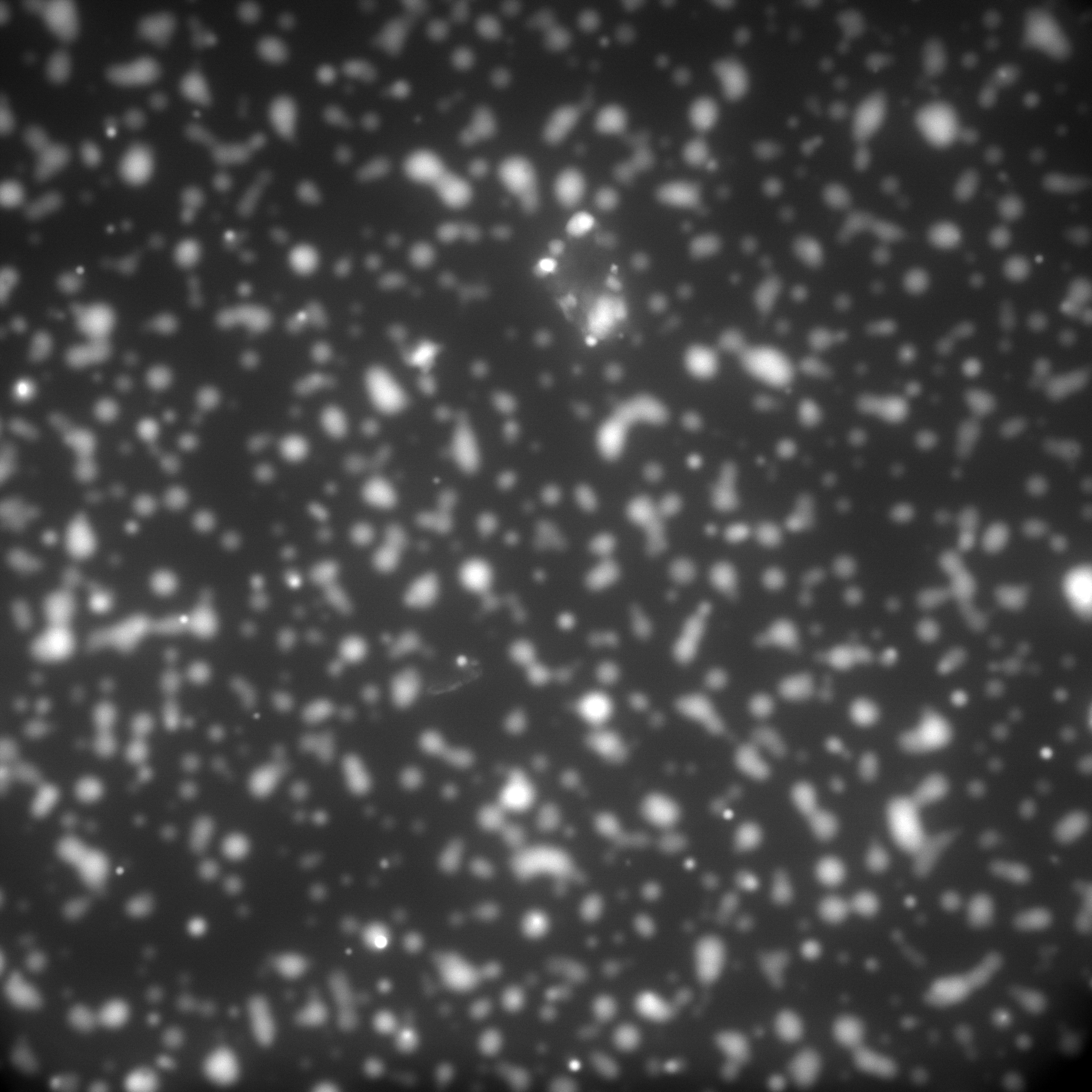

Supplement: Figure 2—figure supplement 2—source data 2. — Extracted numerical parameters are listed in the accompanying spreadsheet. [file elife-83543-fig2-figsupp2-data2.zip › Figure 2 - supplement 2 - source data 2/Figure 2 - supplement 2 - source data 2 - inactive ribozyme - Lys5-24 - surface - 0.5 h.tif]

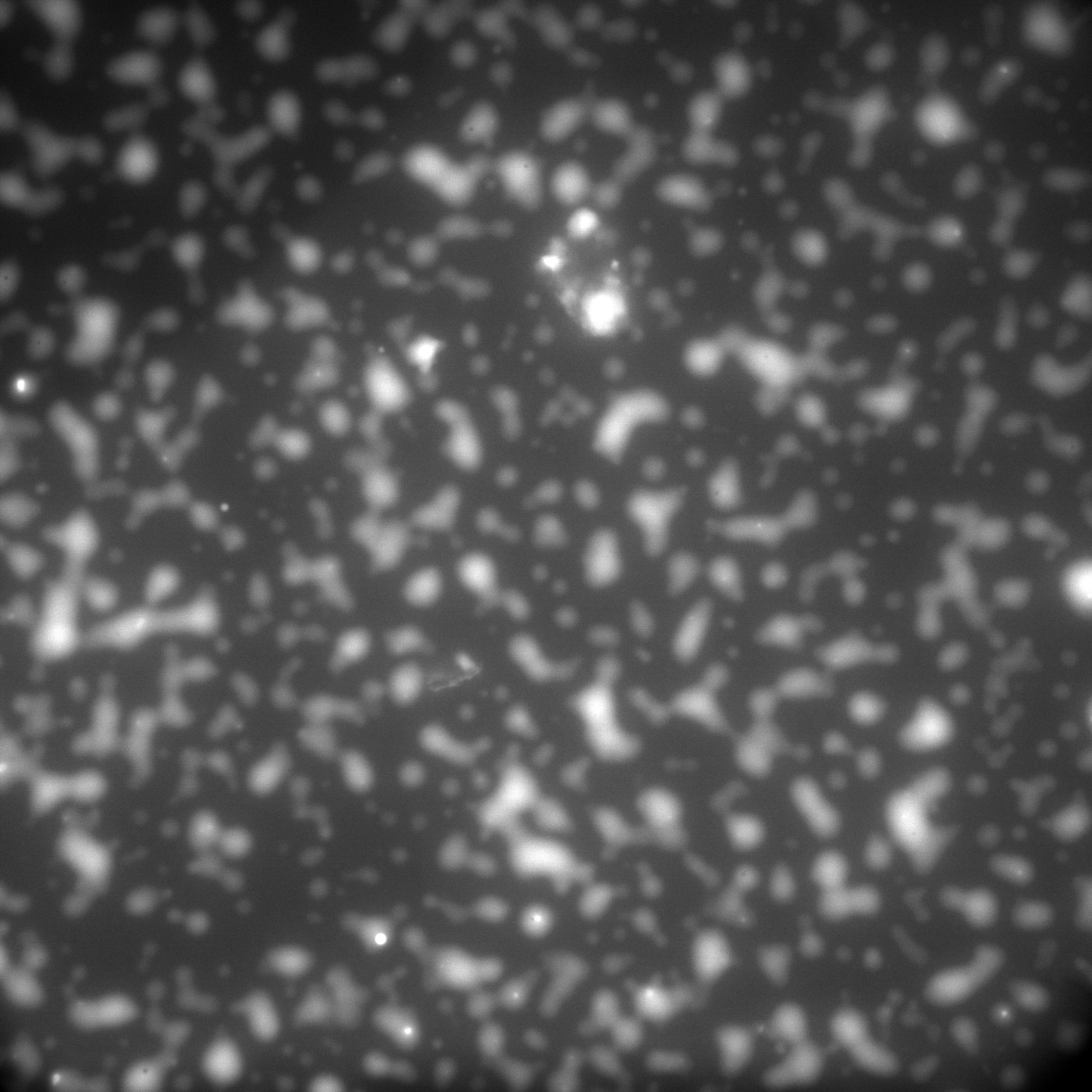

Supplement: Figure 2—figure supplement 2—source data 2. — Extracted numerical parameters are listed in the accompanying spreadsheet. [file elife-83543-fig2-figsupp2-data2.zip › Figure 2 - supplement 2 - source data 2/Figure 2 - supplement 2 - source data 2 - inactive ribozyme - Lys5-24 - surface - 1 h.tif]

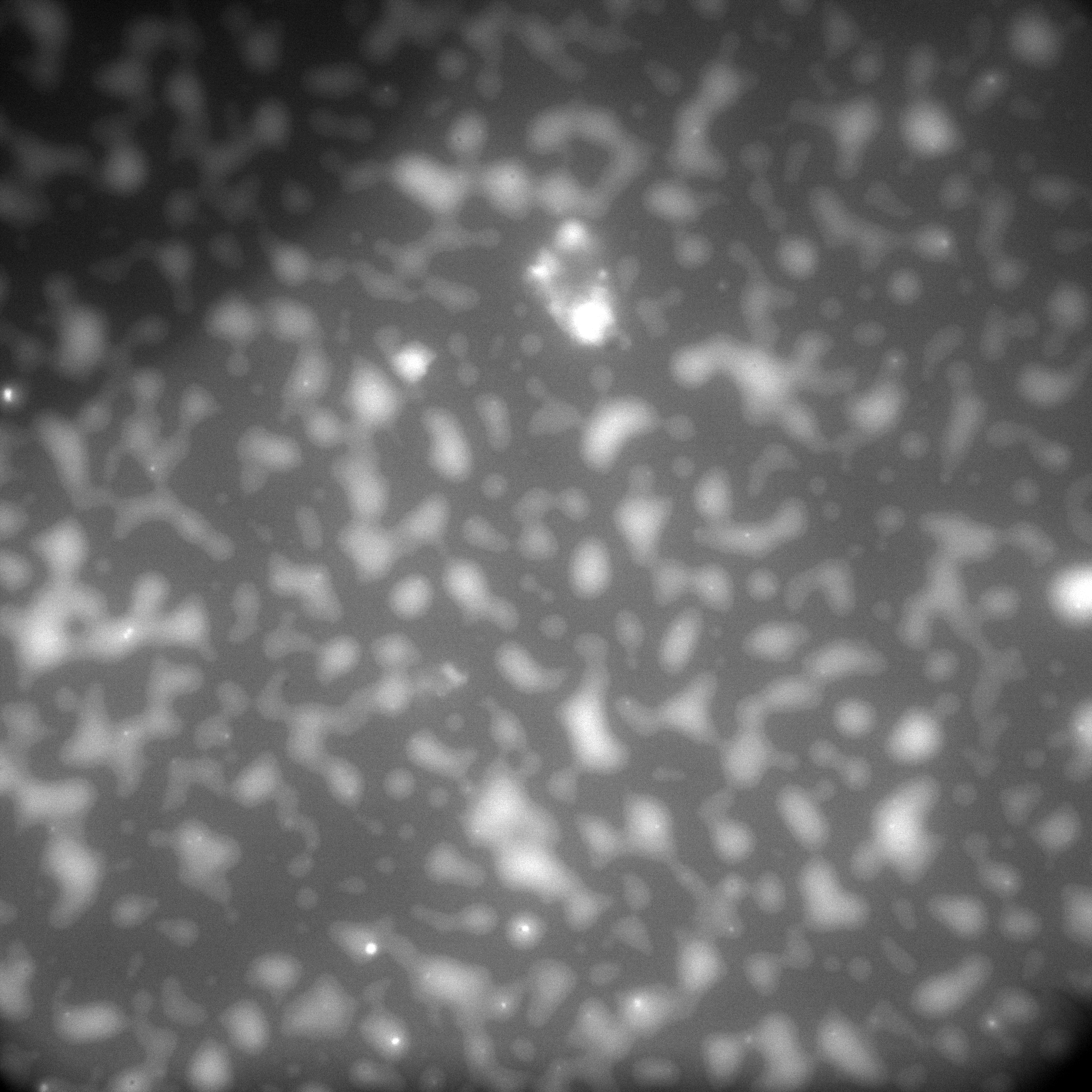

Supplement: Figure 2—figure supplement 2—source data 2. — Extracted numerical parameters are listed in the accompanying spreadsheet. [file elife-83543-fig2-figsupp2-data2.zip › Figure 2 - supplement 2 - source data 2/Figure 2 - supplement 2 - source data 2 - inactive ribozyme - Lys5-24 - surface - 2 h.tif]

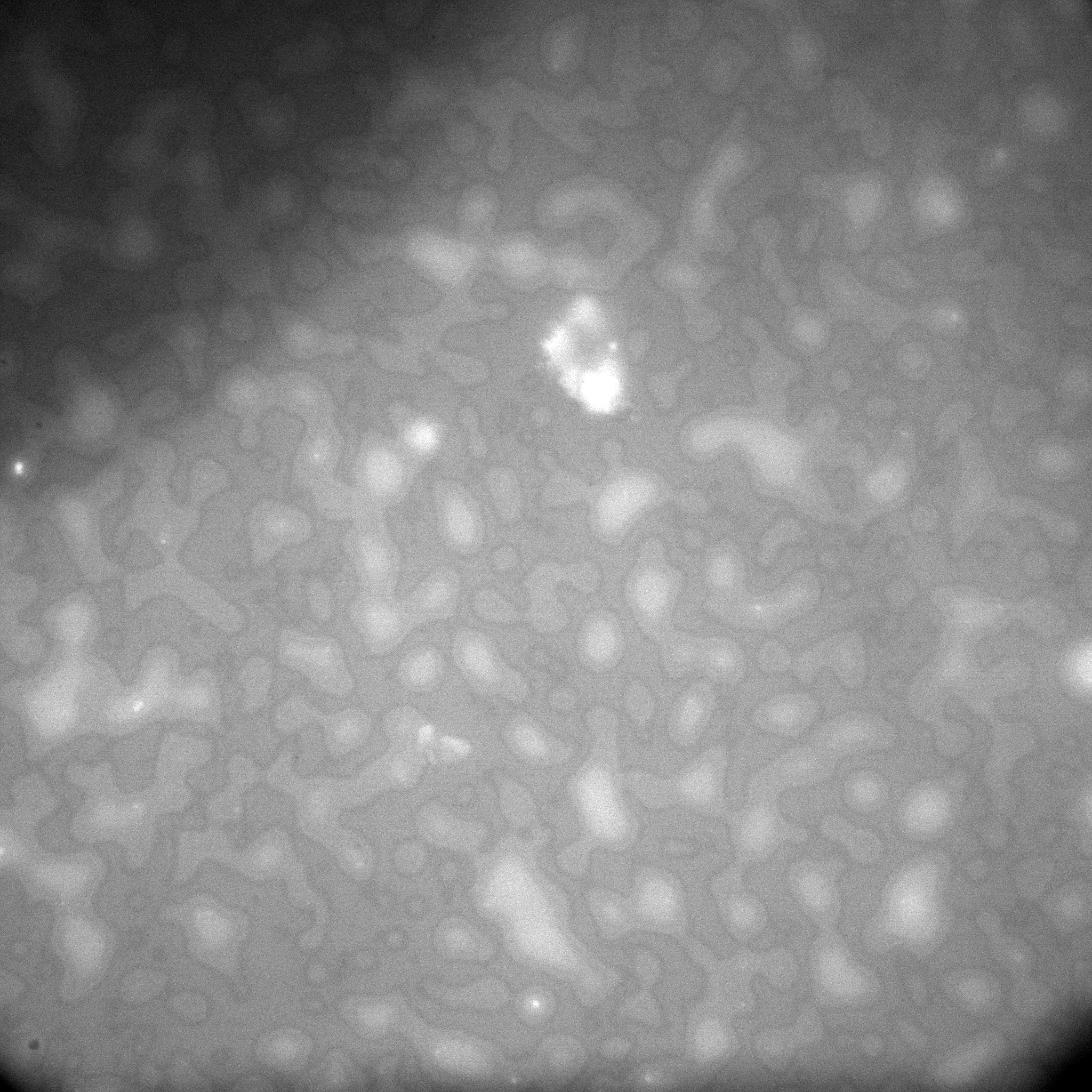

Supplement: Figure 2—figure supplement 2—source data 2. — Extracted numerical parameters are listed in the accompanying spreadsheet. [file elife-83543-fig2-figsupp2-data2.zip › Figure 2 - supplement 2 - source data 2/Figure 2 - supplement 2 - source data 2 - inactive ribozyme - Lys5-24 - surface - 24 h.tif]

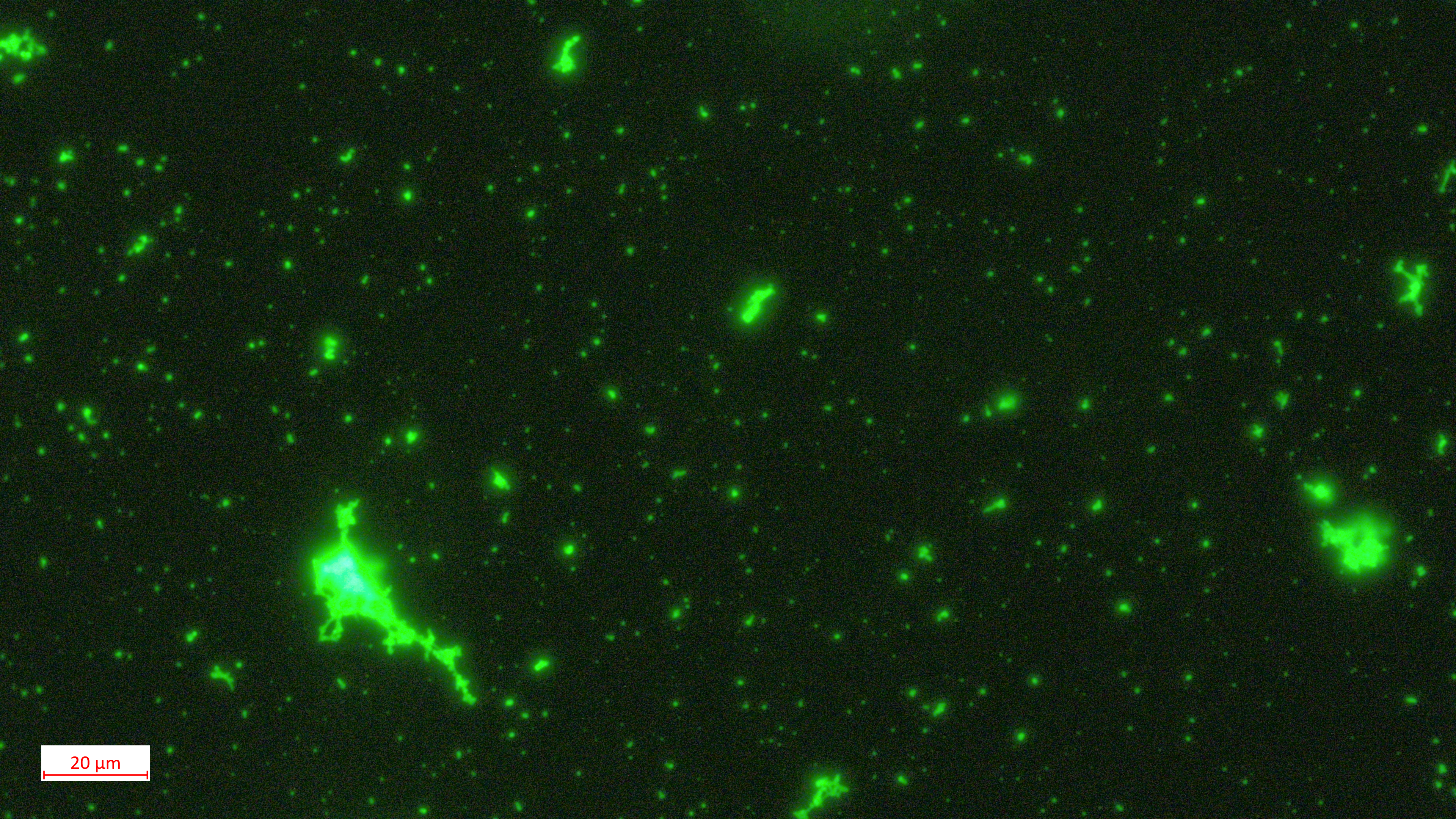

Supplement: Figure 2—figure supplement 5—source data 1. [file elife-83543-fig2-figsupp5-data1.zip › Figure 2 - supplement 5 - source data 1/Figure 2 - supplement 5 - source data 1 - active - Lys19-72 - 1 h.tif]

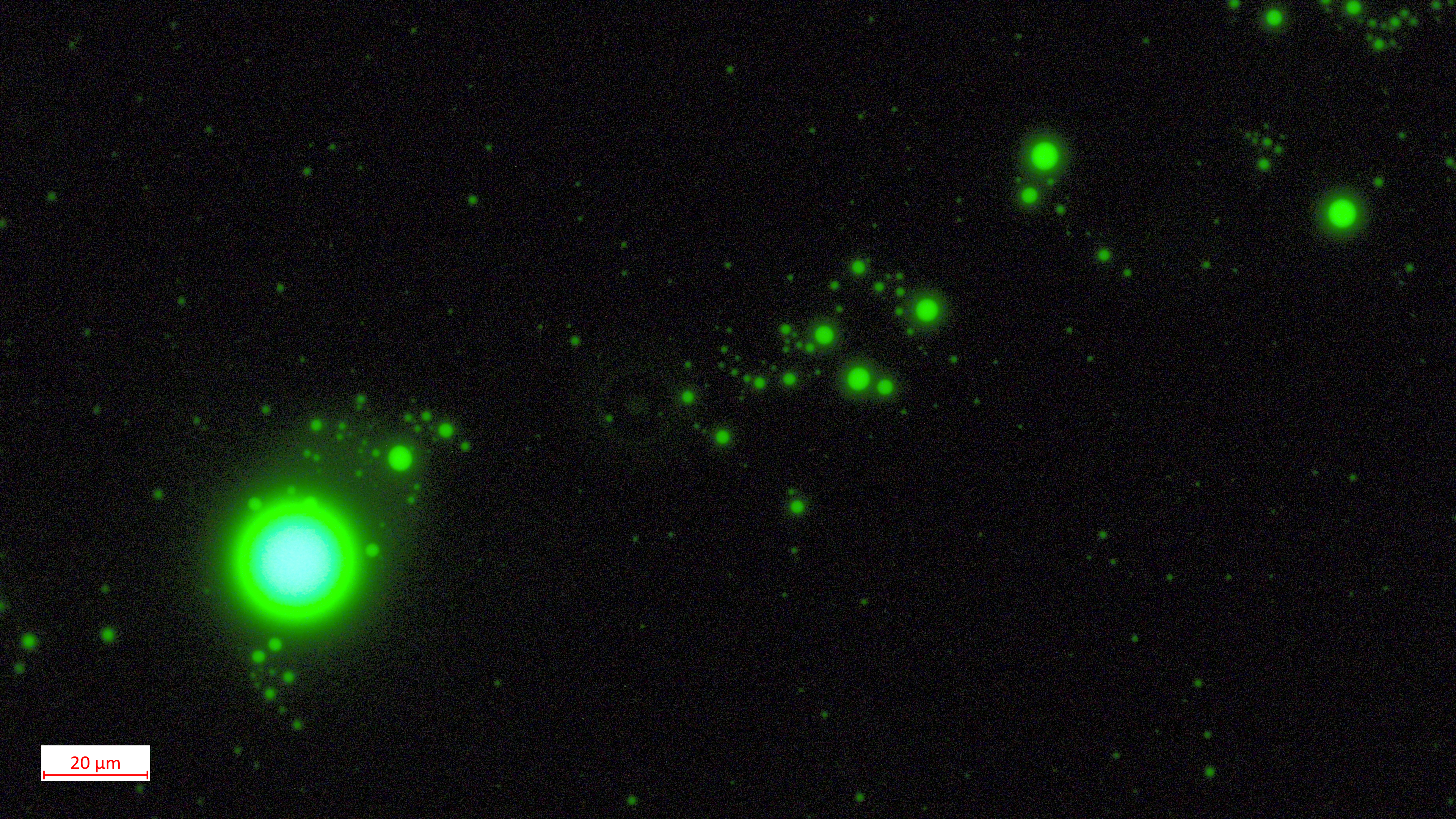

Supplement: Figure 2—figure supplement 5—source data 1. [file elife-83543-fig2-figsupp5-data1.zip › Figure 2 - supplement 5 - source data 1/Figure 2 - supplement 5 - source data 1 - active - Lys19-72 - 24 h.tif]

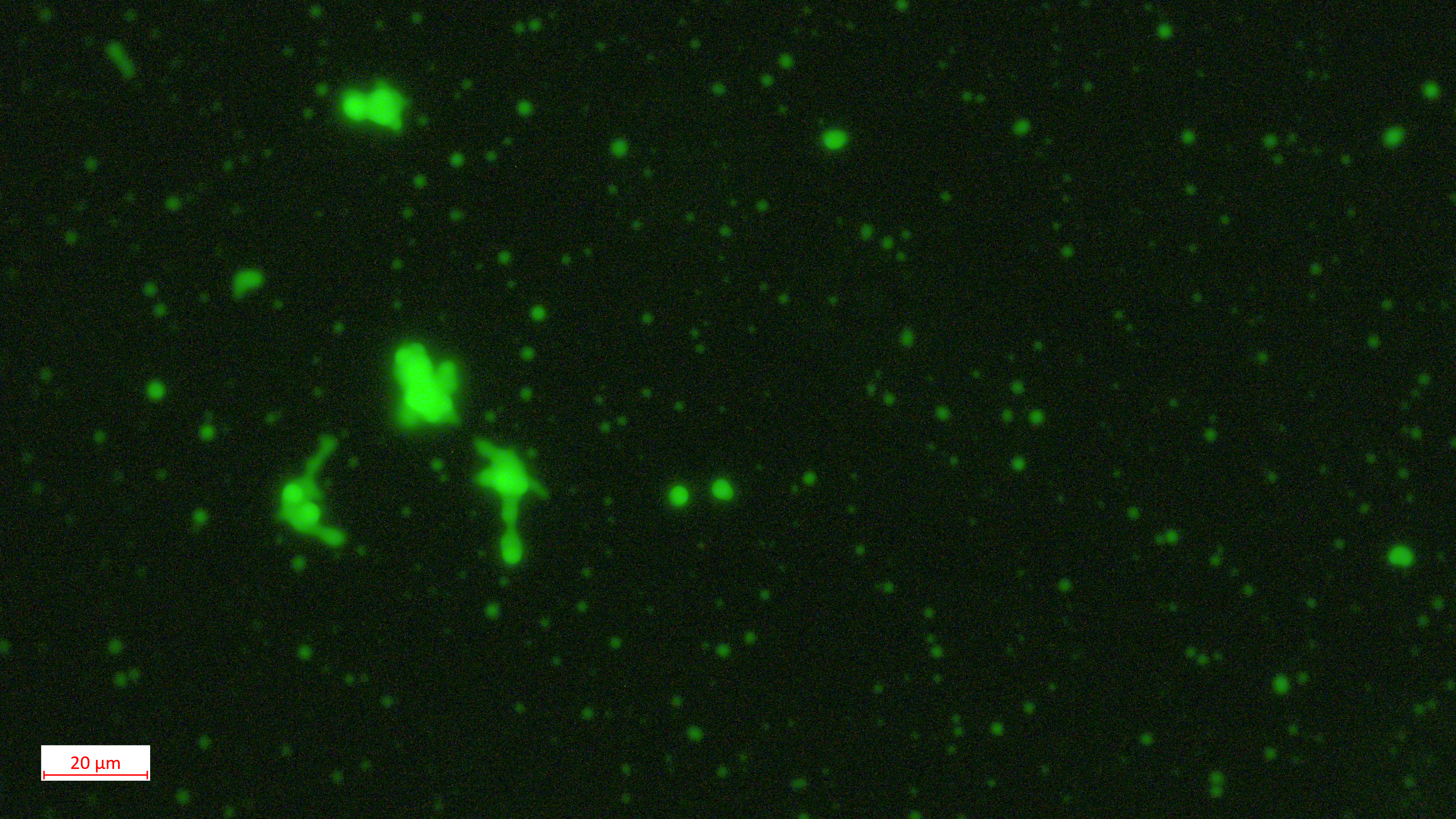

Supplement: Figure 2—figure supplement 5—source data 1. [file elife-83543-fig2-figsupp5-data1.zip › Figure 2 - supplement 5 - source data 1/Figure 2 - supplement 5 - source data 1 - active - Lys5-24 - 1 h.tif]

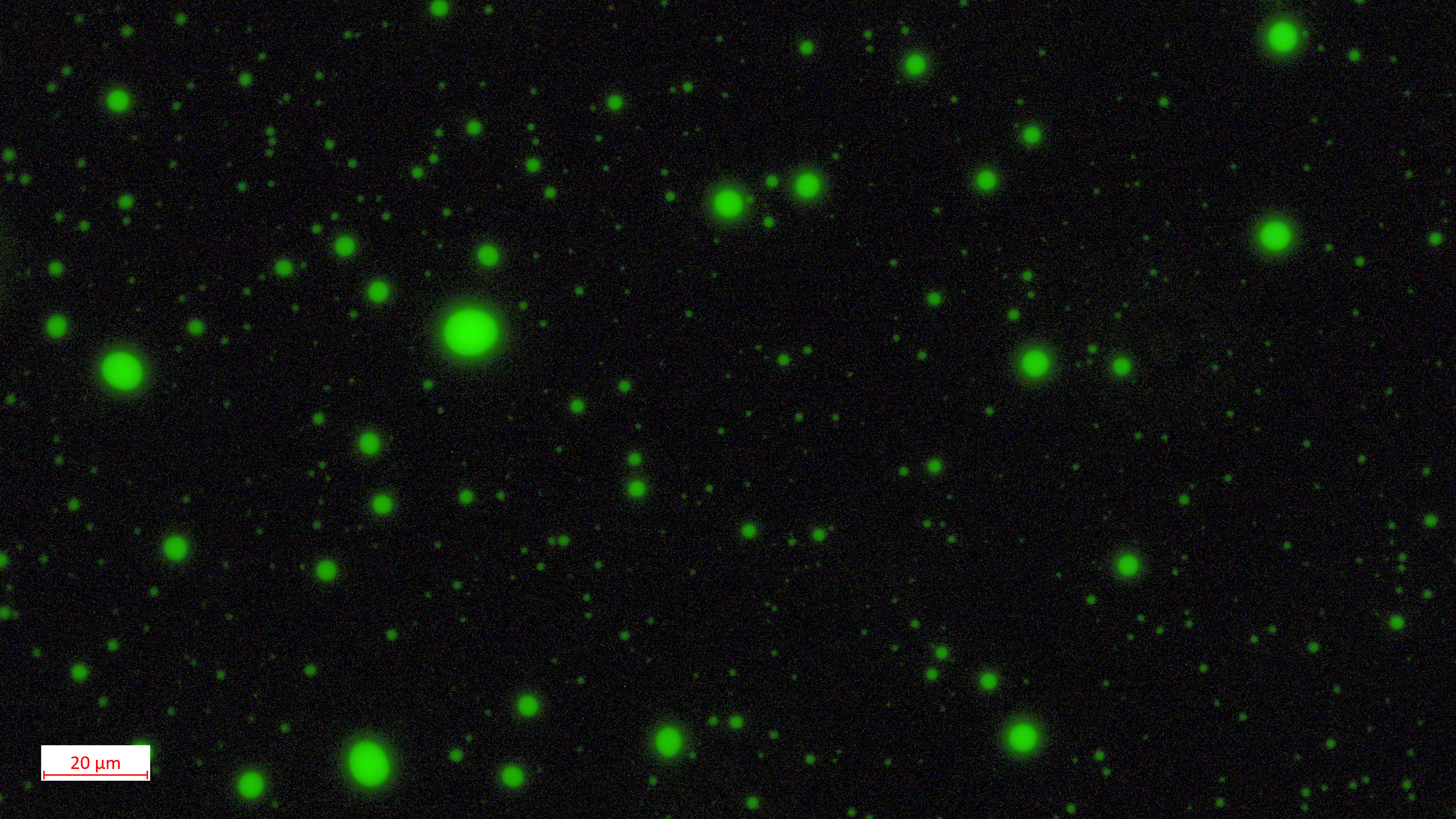

Supplement: Figure 2—figure supplement 5—source data 1. [file elife-83543-fig2-figsupp5-data1.zip › Figure 2 - supplement 5 - source data 1/Figure 2 - supplement 5 - source data 1 - active - Lys5-24 - 24 h.tif]

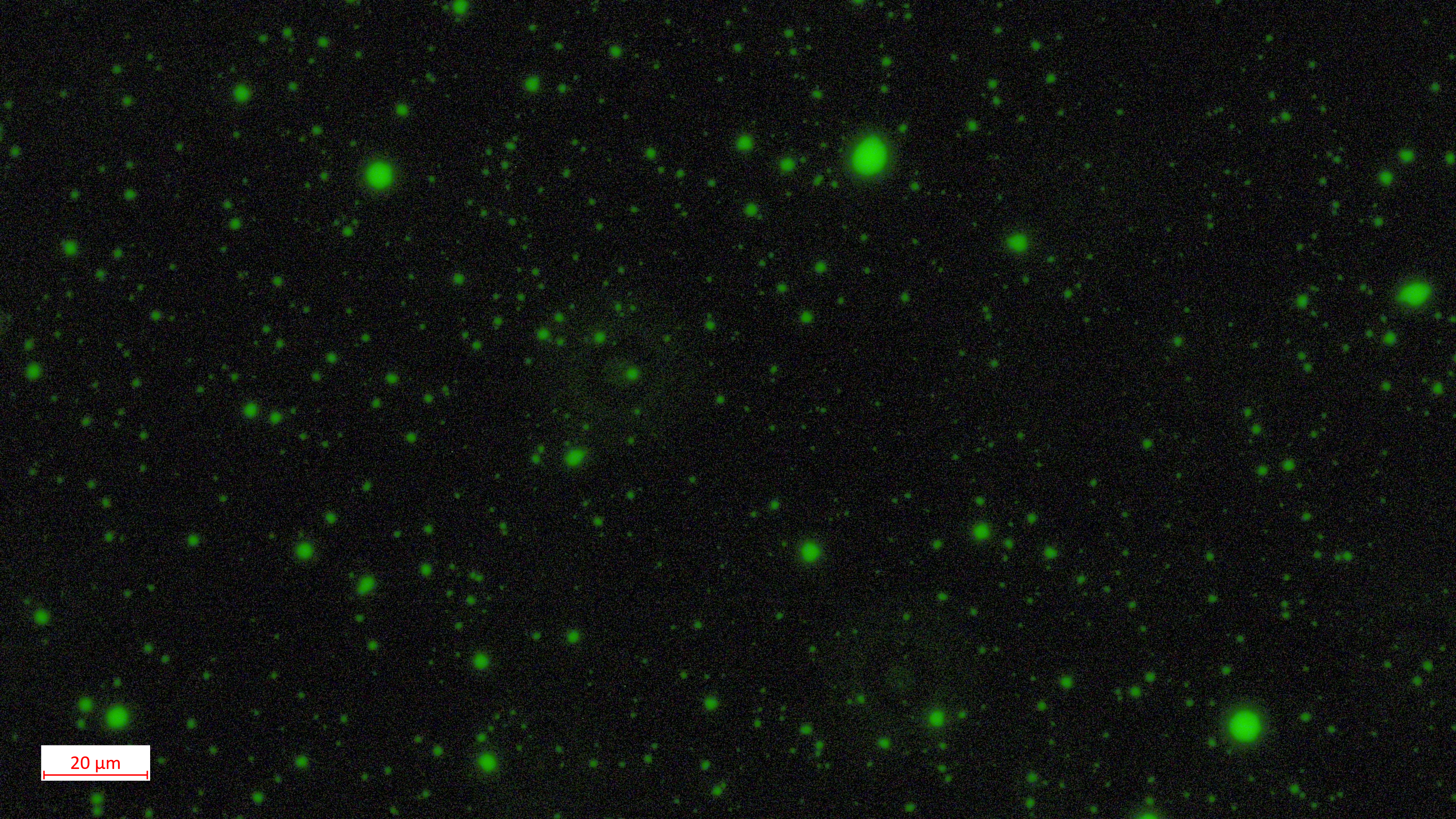

Supplement: Figure 2—figure supplement 5—source data 2. [file elife-83543-fig2-figsupp5-data2.zip › Figure 2 - supplement 5 - source data 2/Figure 2 - supplement 5 - source data 2 - inactive - Lys19-72 - 1 h.tif]

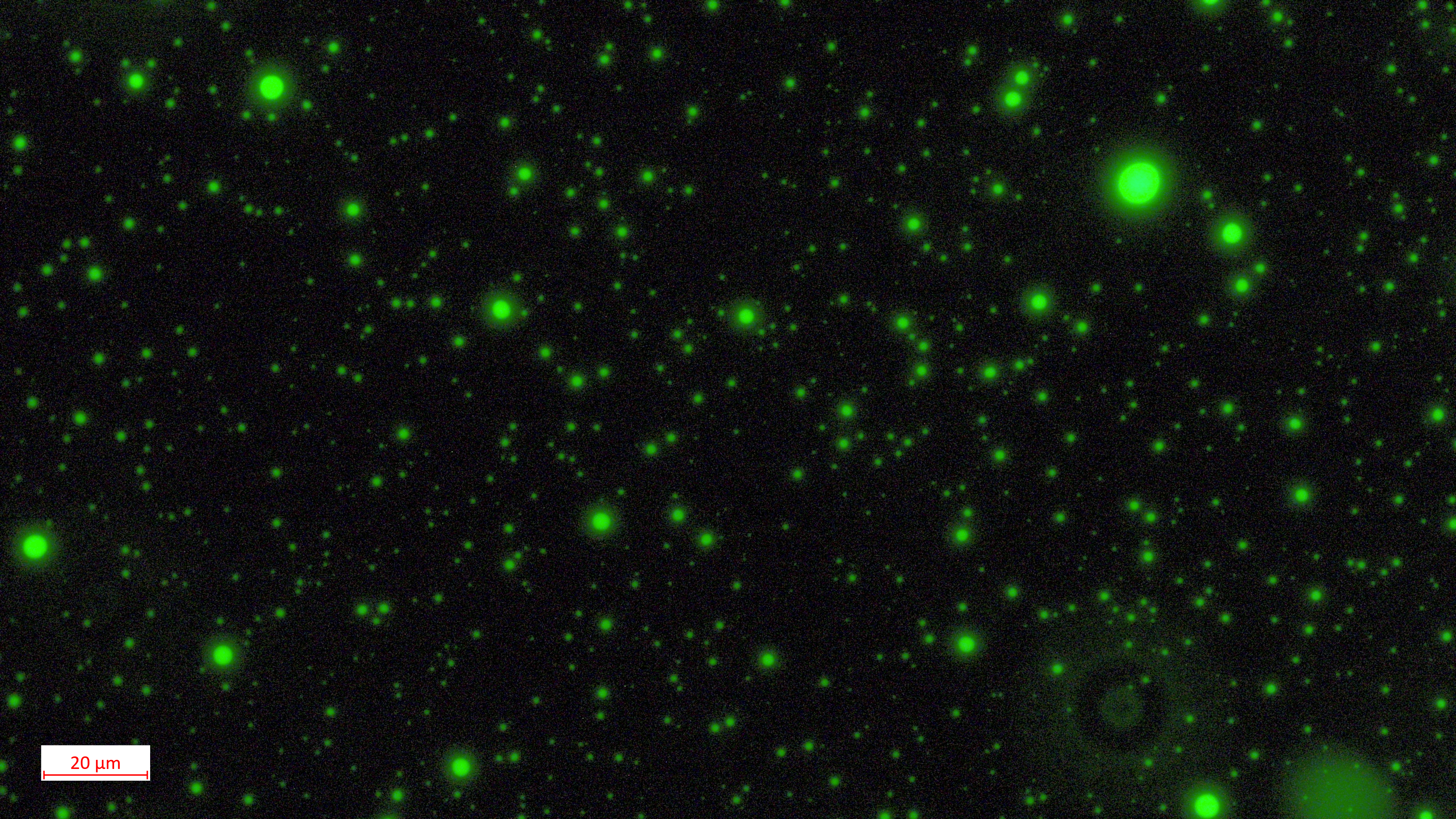

Supplement: Figure 2—figure supplement 5—source data 2. [file elife-83543-fig2-figsupp5-data2.zip › Figure 2 - supplement 5 - source data 2/Figure 2 - supplement 5 - source data 2 - inactive - Lys19-72 - 24 h.tif]

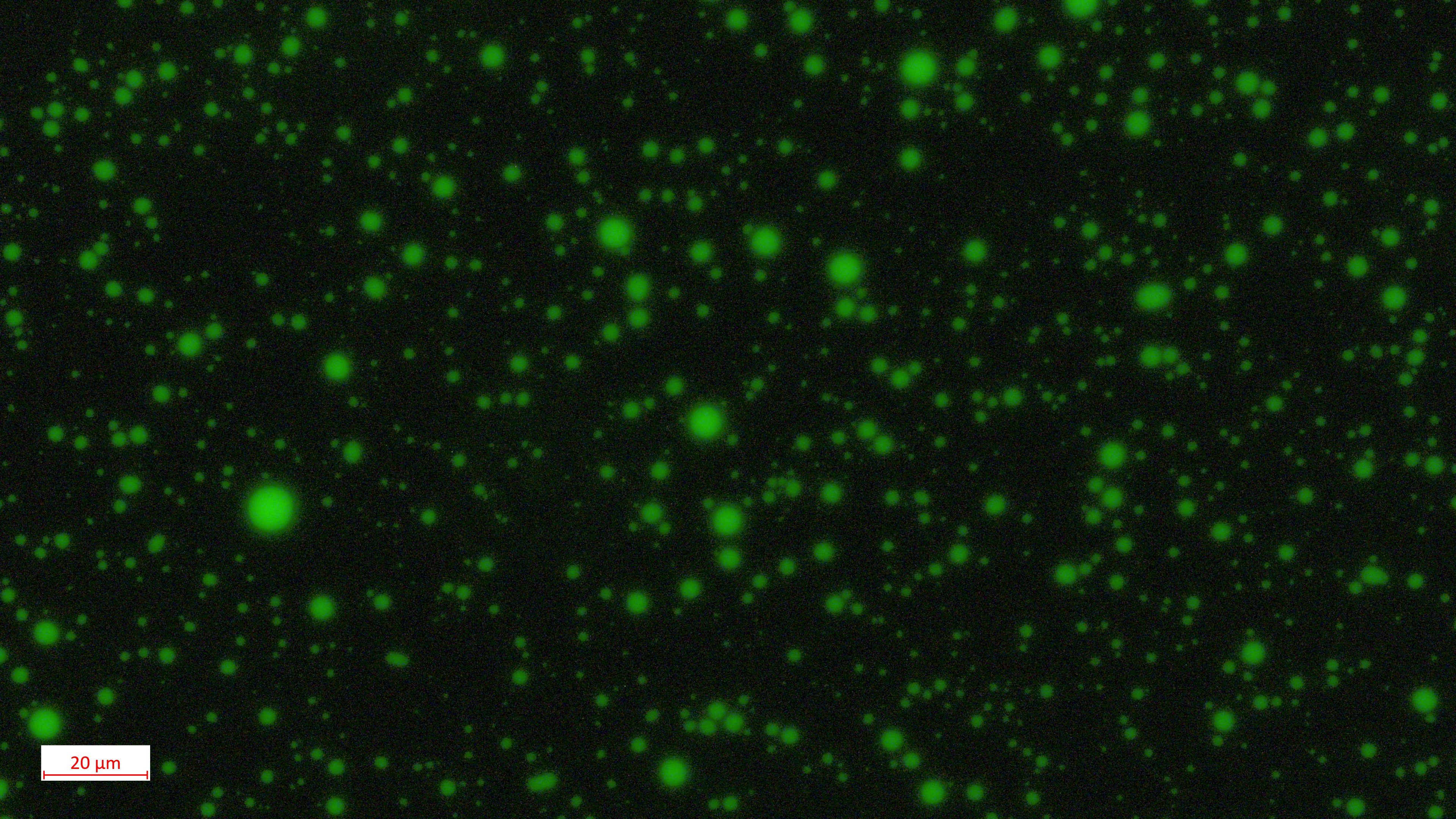

Supplement: Figure 2—figure supplement 5—source data 2. [file elife-83543-fig2-figsupp5-data2.zip › Figure 2 - supplement 5 - source data 2/Figure 2 - supplement 5 - source data 2 - inactive - Lys5-24 - 1 h.tif]

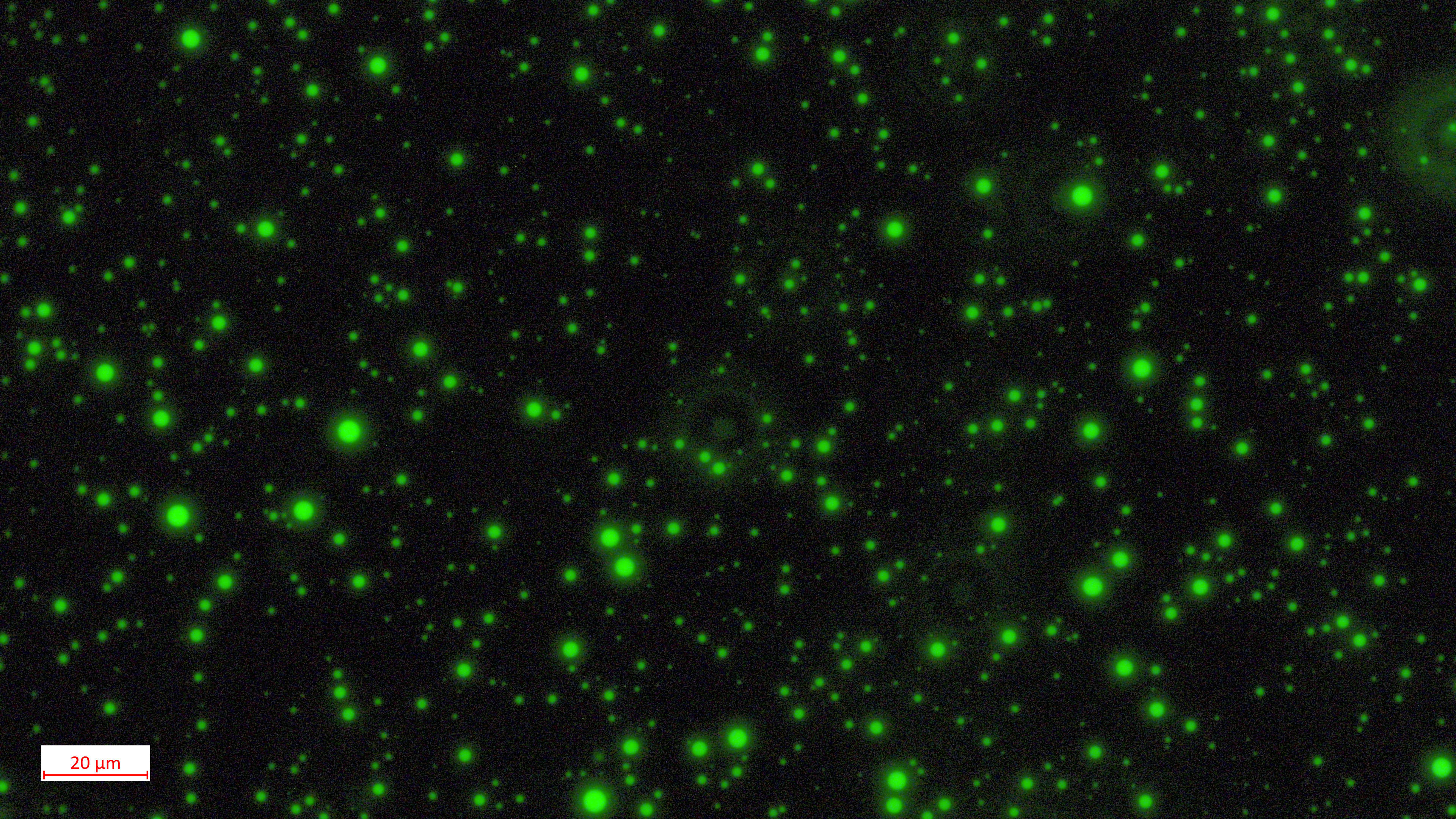

Supplement: Figure 2—figure supplement 5—source data 2. [file elife-83543-fig2-figsupp5-data2.zip › Figure 2 - supplement 5 - source data 2/Figure 2 - supplement 5 - source data 2 - inactive - Lys5-24 - 24 h.tif]

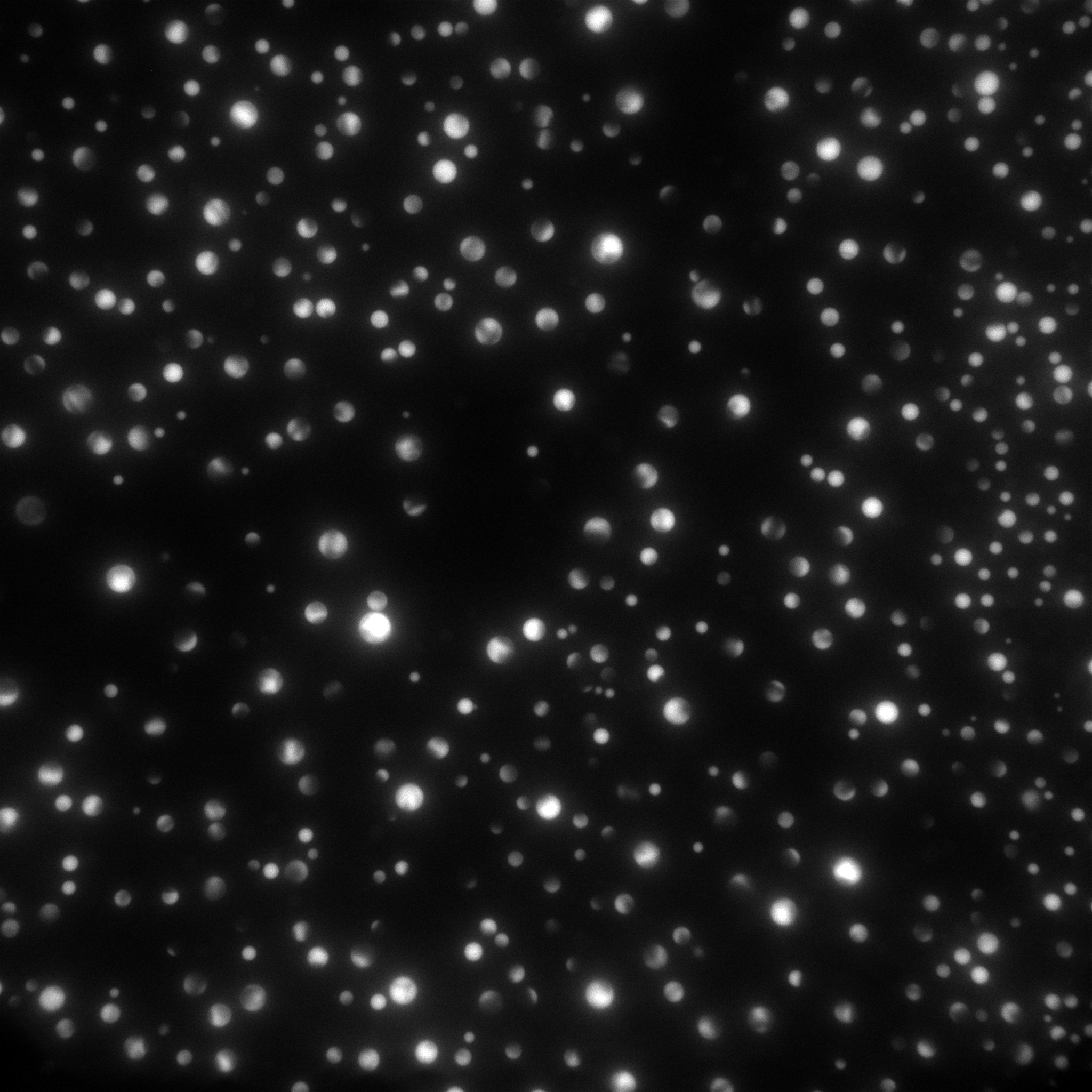

Supplement: Figure 3—source data 1. — Extracted numerical parameters are listed in the accompanying spreadsheet. [file elife-83543-fig3-data1.zip › Figure 3 - source data 1/Figure 3 - source data 1 - active - Lys19-72 - 0.5 h.tif]

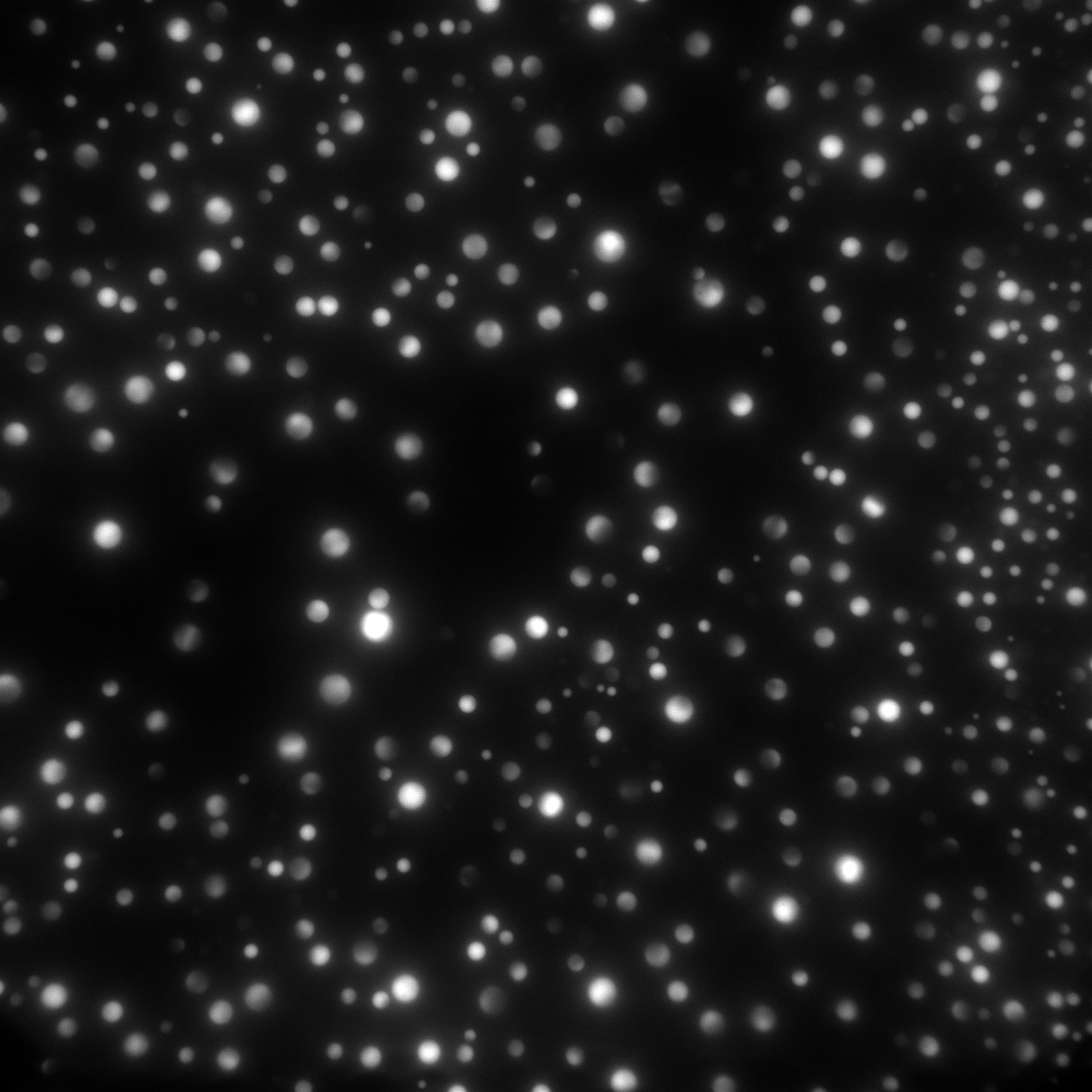

Supplement: Figure 3—source data 1. — Extracted numerical parameters are listed in the accompanying spreadsheet. [file elife-83543-fig3-data1.zip › Figure 3 - source data 1/Figure 3 - source data 1 - active - Lys19-72 - 1 h.tif]

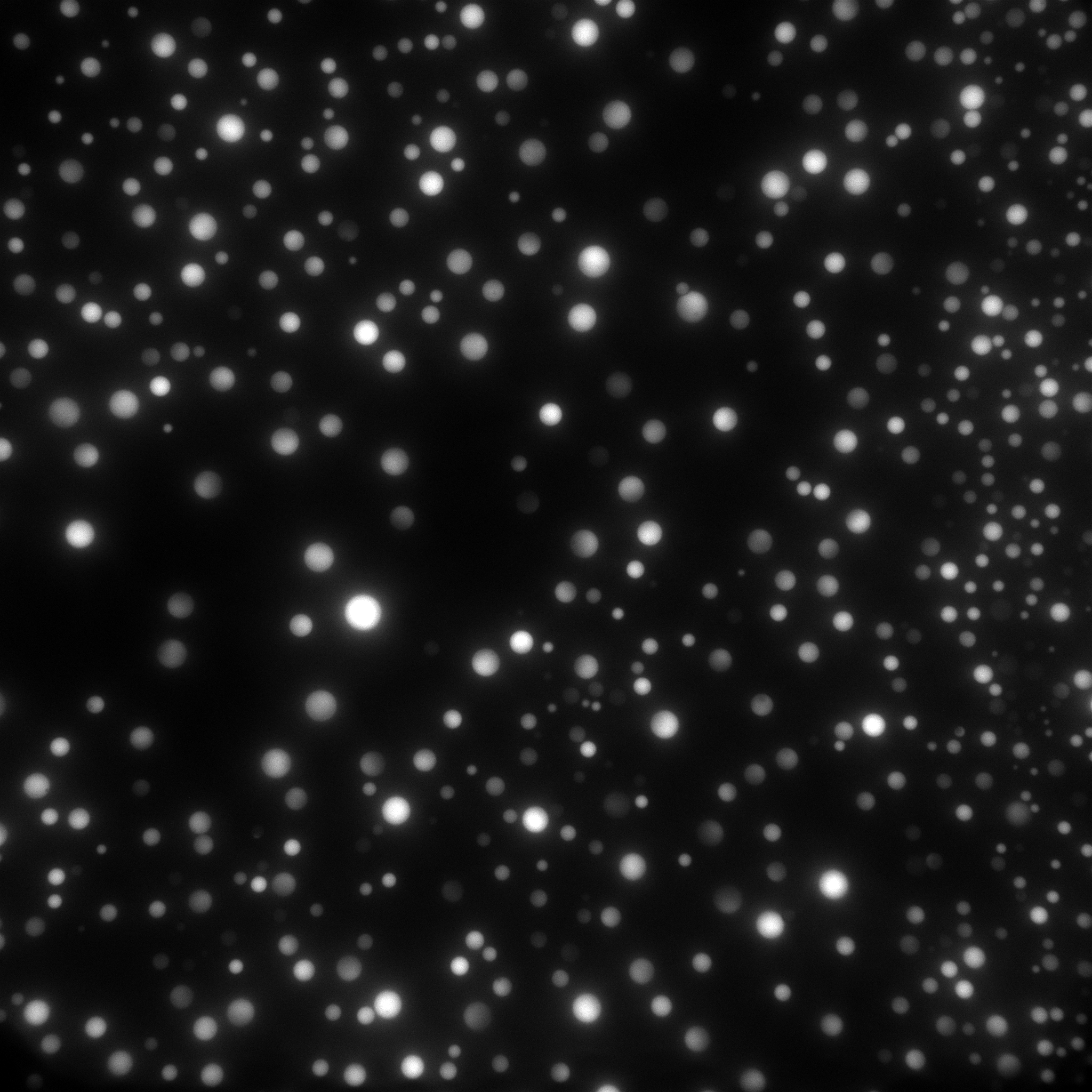

Supplement: Figure 3—source data 1. — Extracted numerical parameters are listed in the accompanying spreadsheet. [file elife-83543-fig3-data1.zip › Figure 3 - source data 1/Figure 3 - source data 1 - active - Lys19-72 - 2 h.tif]

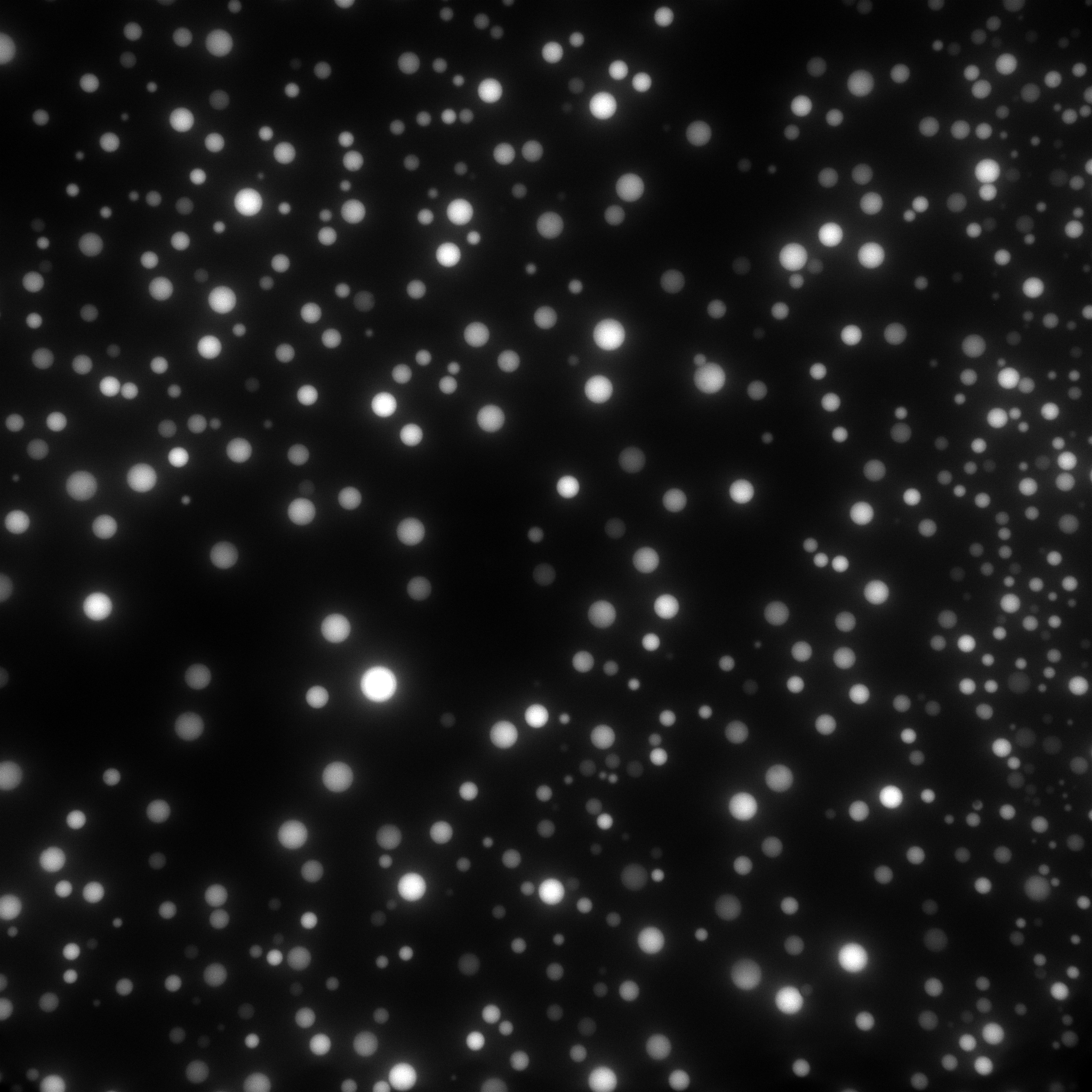

Supplement: Figure 3—source data 1. — Extracted numerical parameters are listed in the accompanying spreadsheet. [file elife-83543-fig3-data1.zip › Figure 3 - source data 1/Figure 3 - source data 1 - active - Lys19-72 - 24 h.tif]

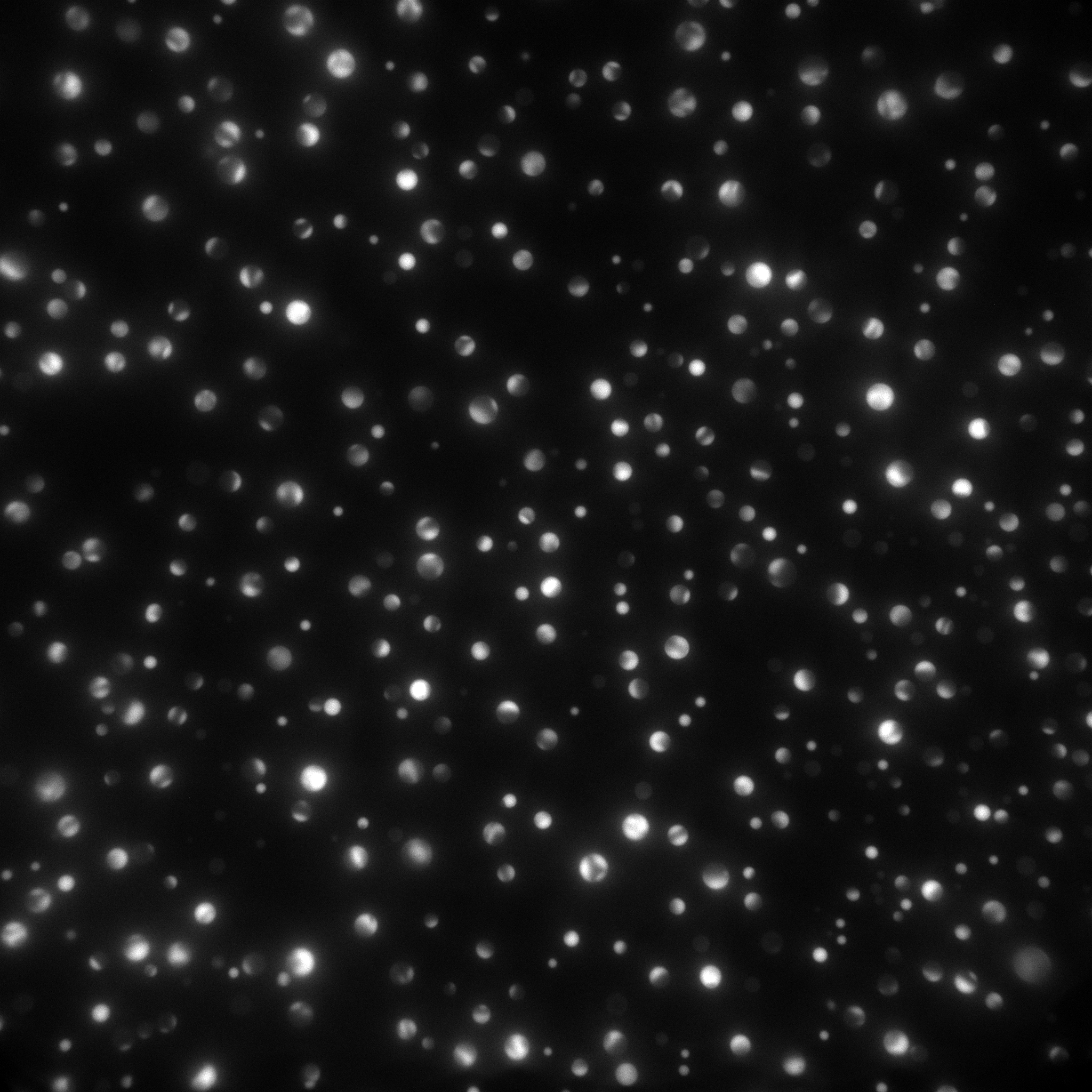

Supplement: Figure 3—source data 2. — Extracted numerical parameters are listed in the accompanying spreadsheet. [file elife-83543-fig3-data2.zip › Figure 3 - source data 2/Figure 3 - source data 2 - inactive - Lys19-72 - 0.5 h.tif]

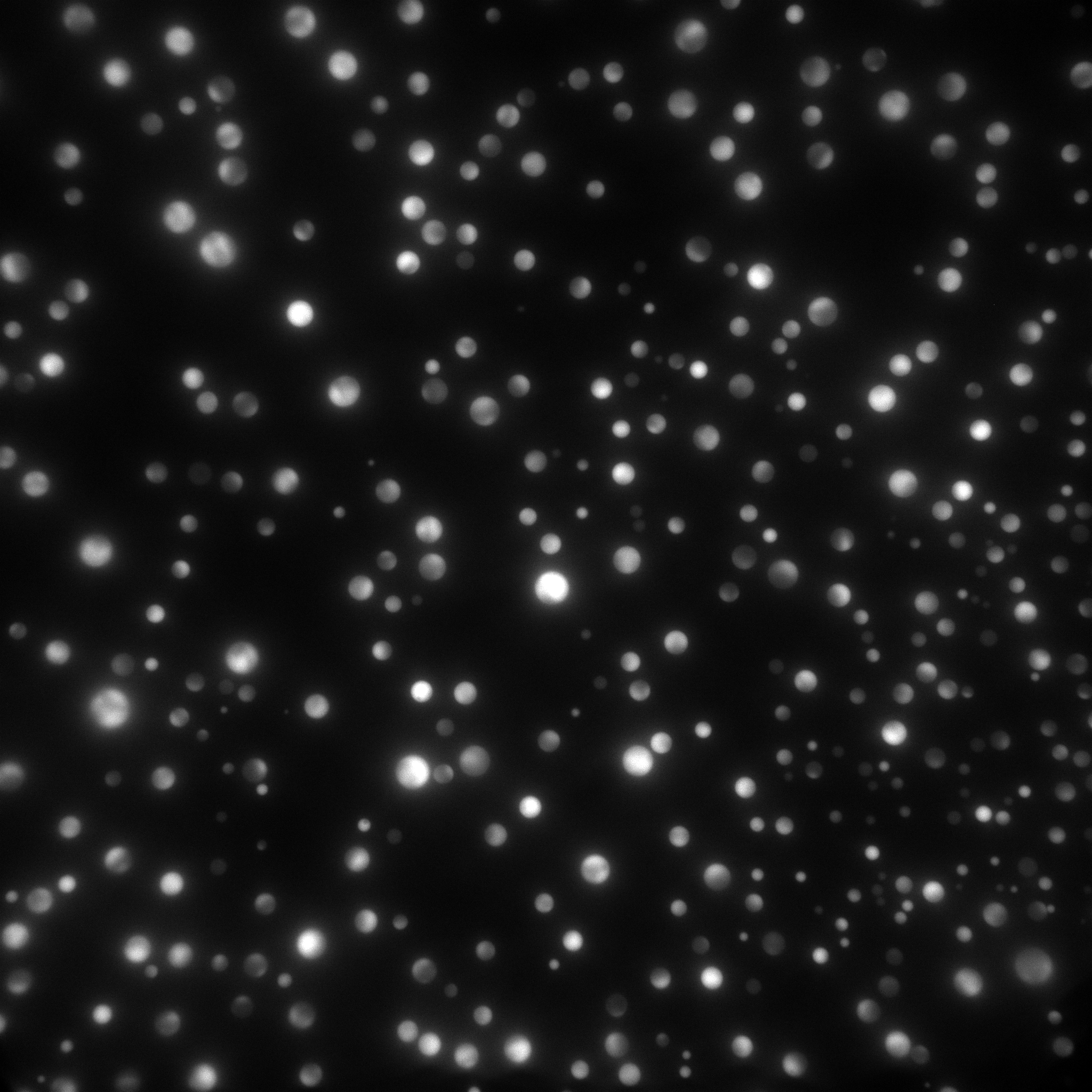

Supplement: Figure 3—source data 2. — Extracted numerical parameters are listed in the accompanying spreadsheet. [file elife-83543-fig3-data2.zip › Figure 3 - source data 2/Figure 3 - source data 2 - inactive - Lys19-72 - 1 h.tif]

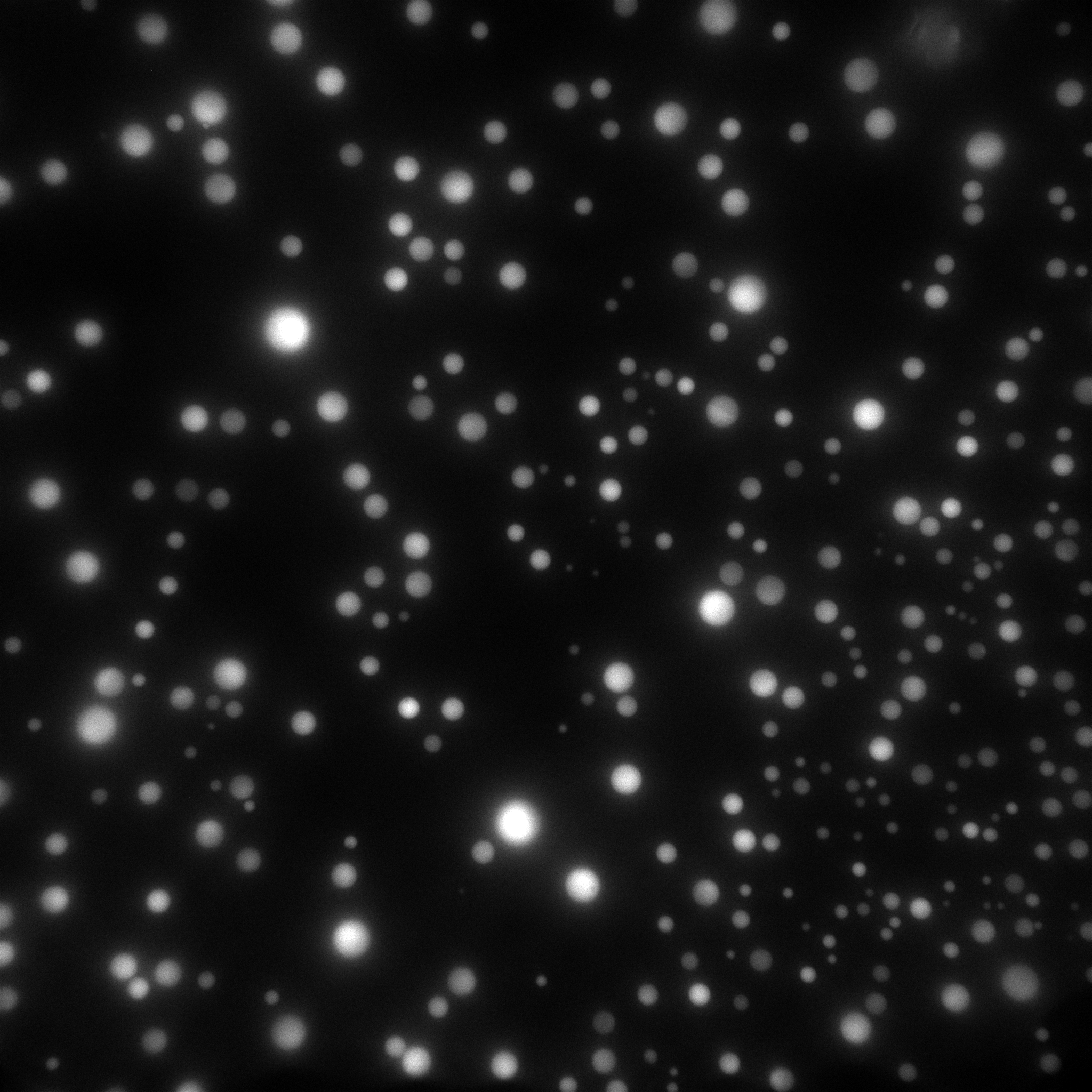

Supplement: Figure 3—source data 2. — Extracted numerical parameters are listed in the accompanying spreadsheet. [file elife-83543-fig3-data2.zip › Figure 3 - source data 2/Figure 3 - source data 2 - inactive - Lys19-72 - 2 h.tif]

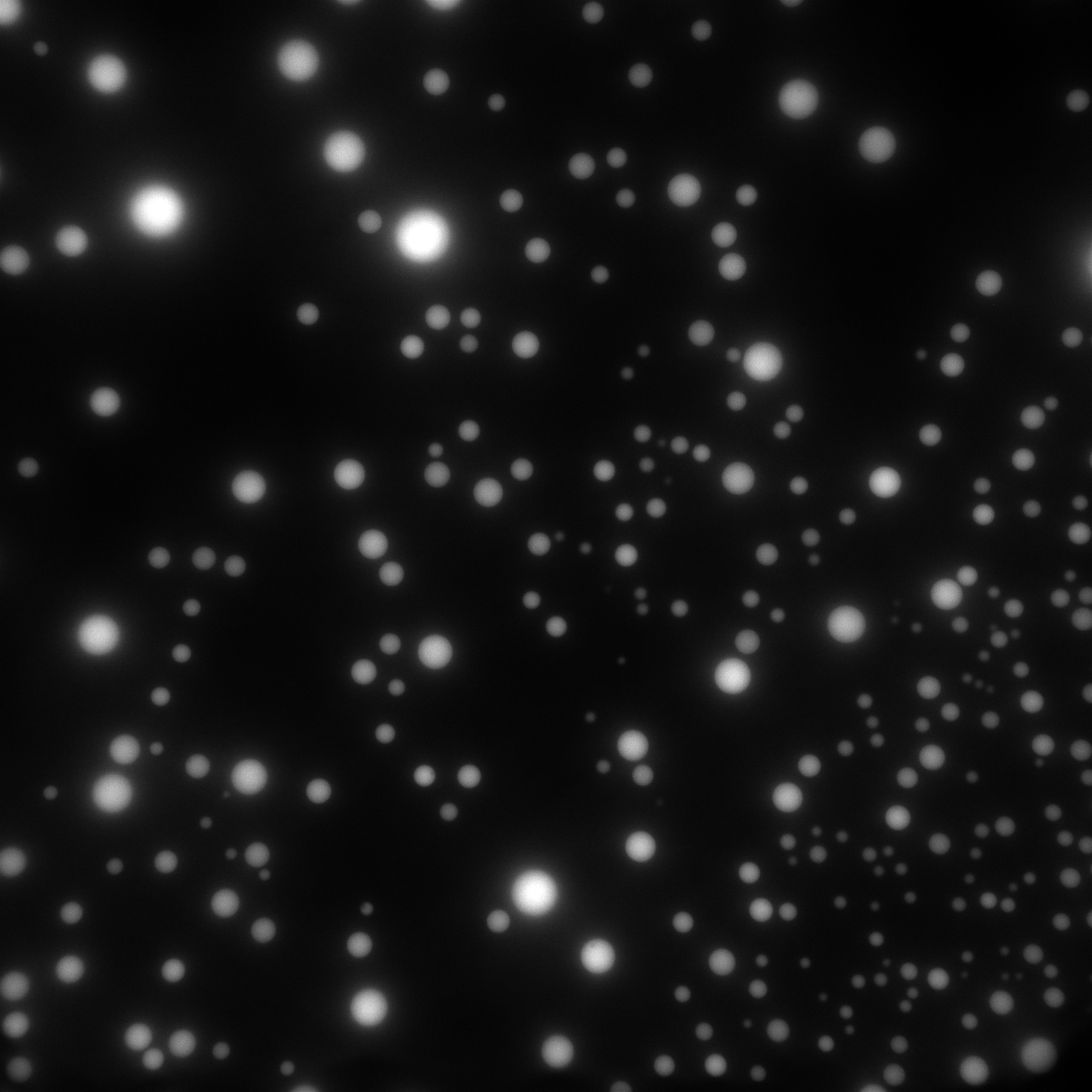

Supplement: Figure 3—source data 2. — Extracted numerical parameters are listed in the accompanying spreadsheet. [file elife-83543-fig3-data2.zip › Figure 3 - source data 2/Figure 3 - source data 2 - inactive - Lys19-72 - 24 h.tif]

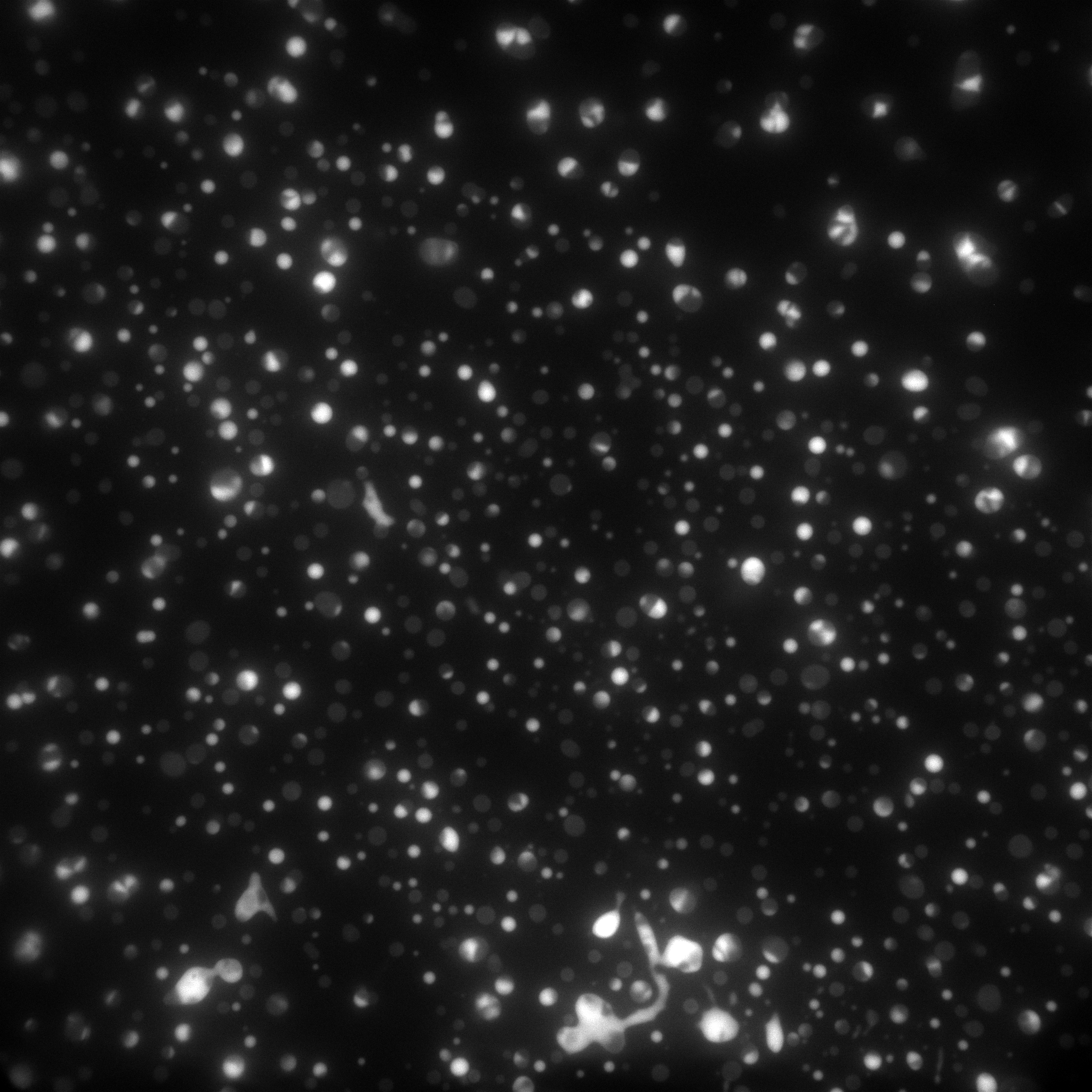

Supplement: Figure 3—figure supplement 1—source data 1. — Extracted numerical parameters are listed in the accompanying spreadsheet. [file elife-83543-fig3-figsupp1-data1.zip › Figure 3 - supplement 1 - source data 1/Figure 3 - supplement 1 - source data 1 - active - Lys5-24 - 0.5 h.tif]

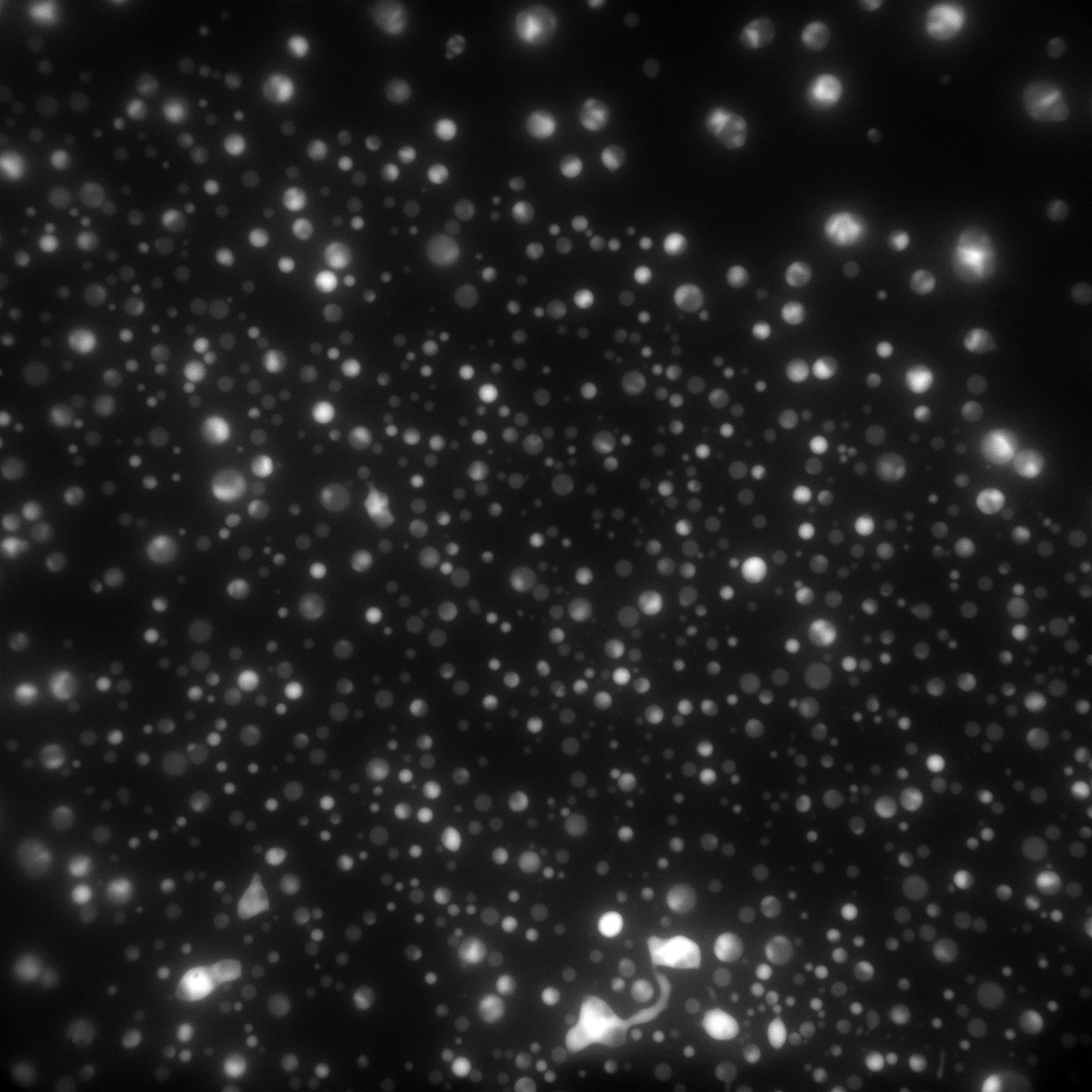

Supplement: Figure 3—figure supplement 1—source data 1. — Extracted numerical parameters are listed in the accompanying spreadsheet. [file elife-83543-fig3-figsupp1-data1.zip › Figure 3 - supplement 1 - source data 1/Figure 3 - supplement 1 - source data 1 - active - Lys5-24 - 1 h.tif]

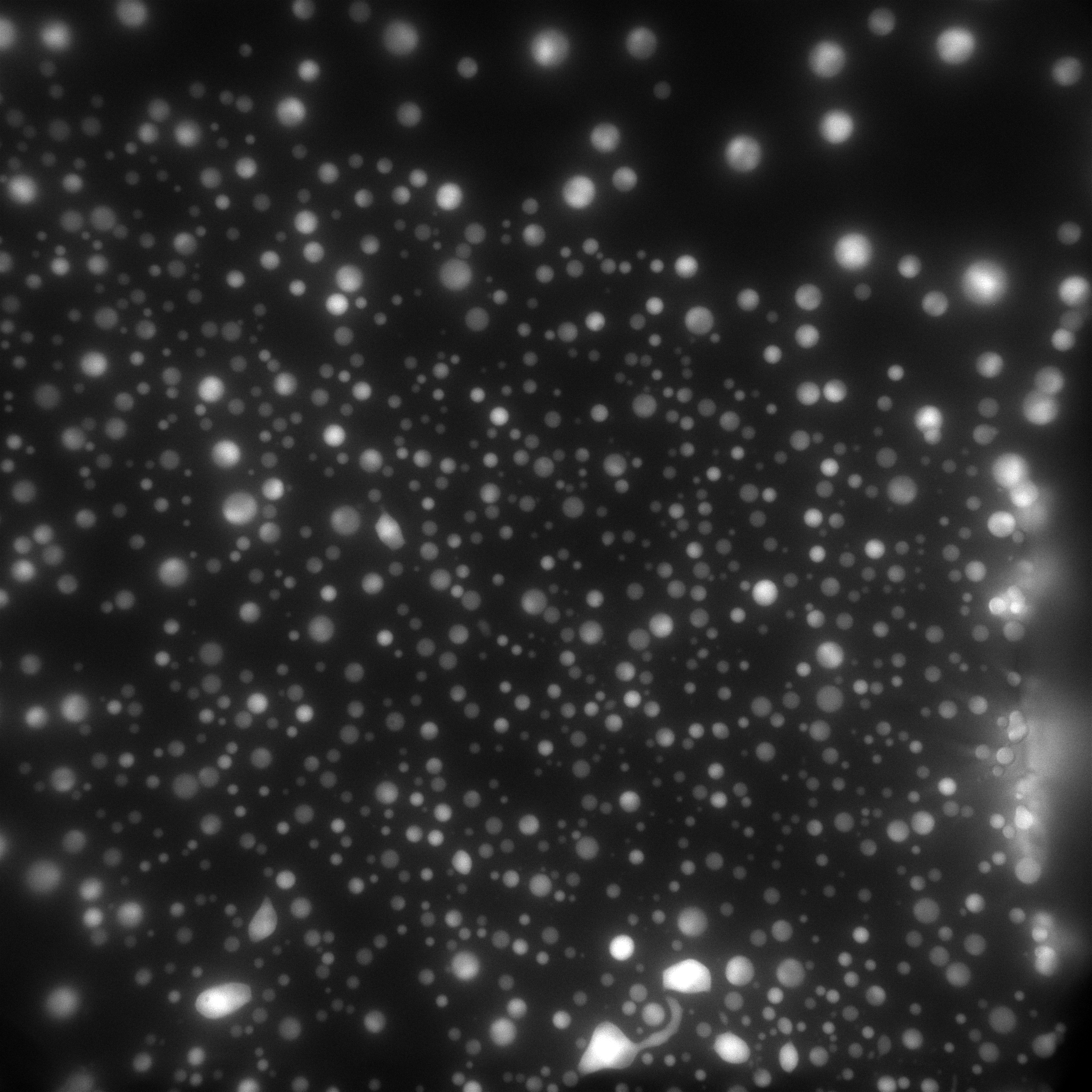

Supplement: Figure 3—figure supplement 1—source data 1. — Extracted numerical parameters are listed in the accompanying spreadsheet. [file elife-83543-fig3-figsupp1-data1.zip › Figure 3 - supplement 1 - source data 1/Figure 3 - supplement 1 - source data 1 - active - Lys5-24 - 2 h.tif]

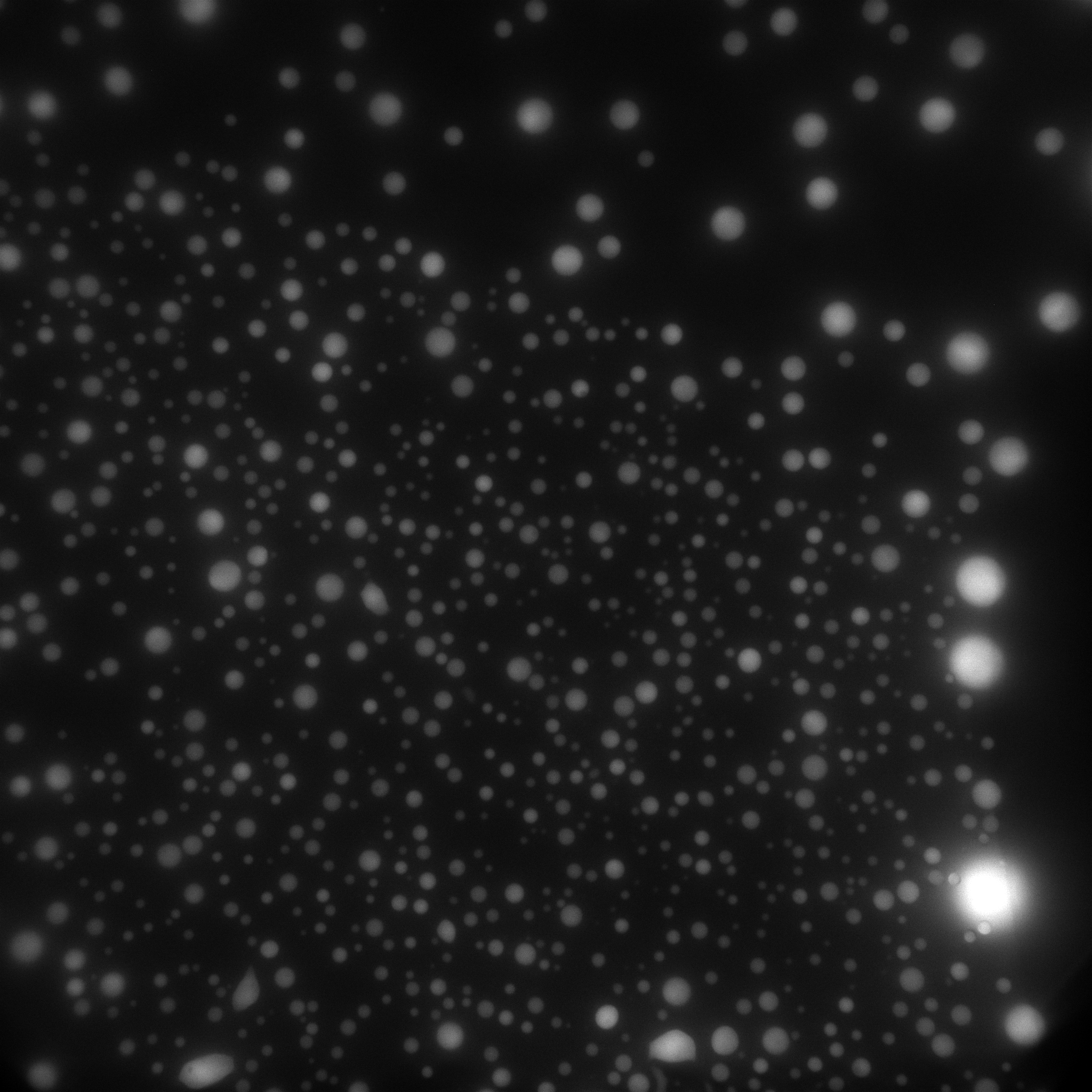

Supplement: Figure 3—figure supplement 1—source data 1. — Extracted numerical parameters are listed in the accompanying spreadsheet. [file elife-83543-fig3-figsupp1-data1.zip › Figure 3 - supplement 1 - source data 1/Figure 3 - supplement 1 - source data 1 - active - Lys5-24 - 24 h.tif]

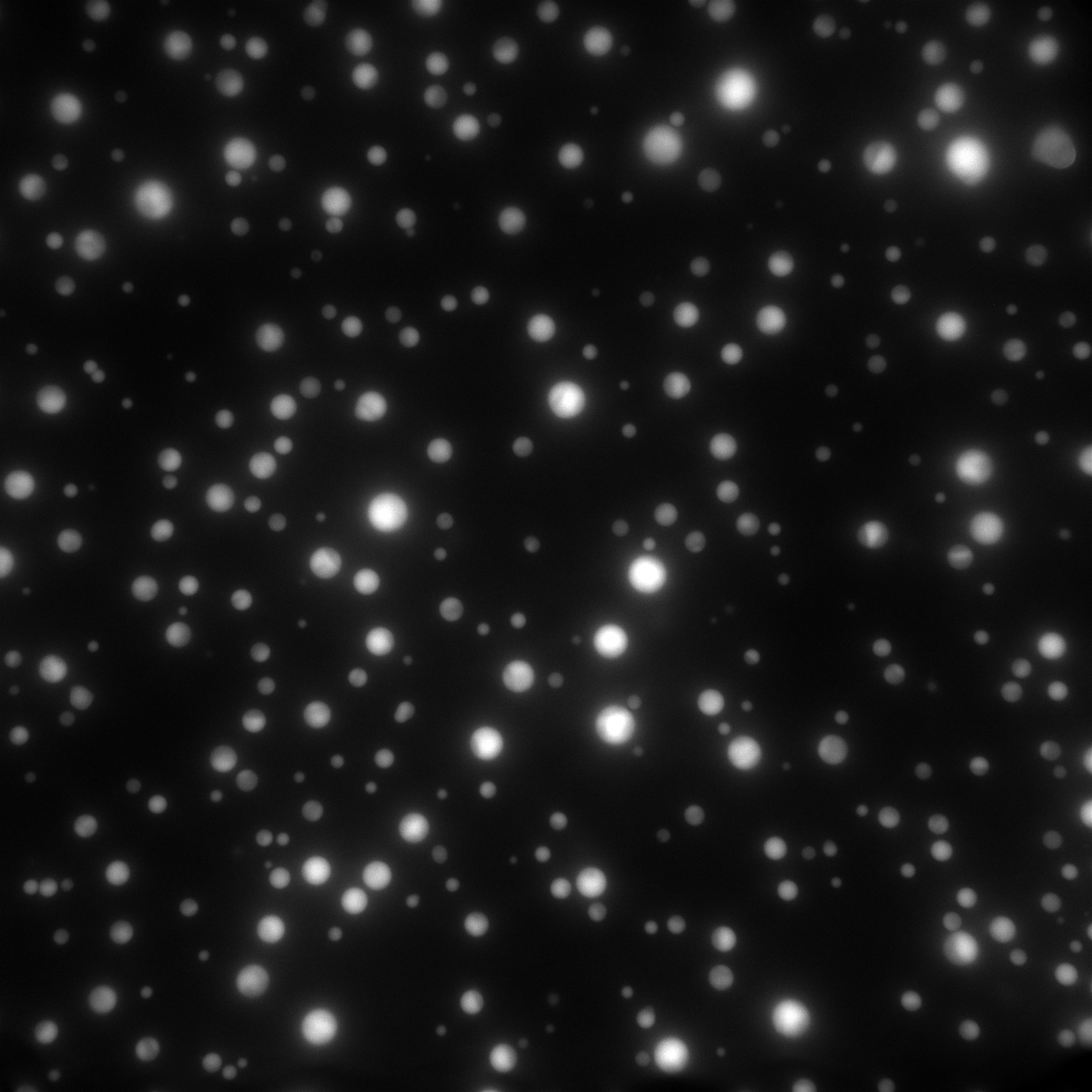

Supplement: Figure 3—figure supplement 1—source data 2. — Extracted numerical parameters are listed in the accompanying spreadsheet. [file elife-83543-fig3-figsupp1-data2.zip › Figure 3 - supplement 1 - source data 2/Figure 3 - supplement 1 - source data 2 - inactive - Lys5-24 - 0.5 h.tif]

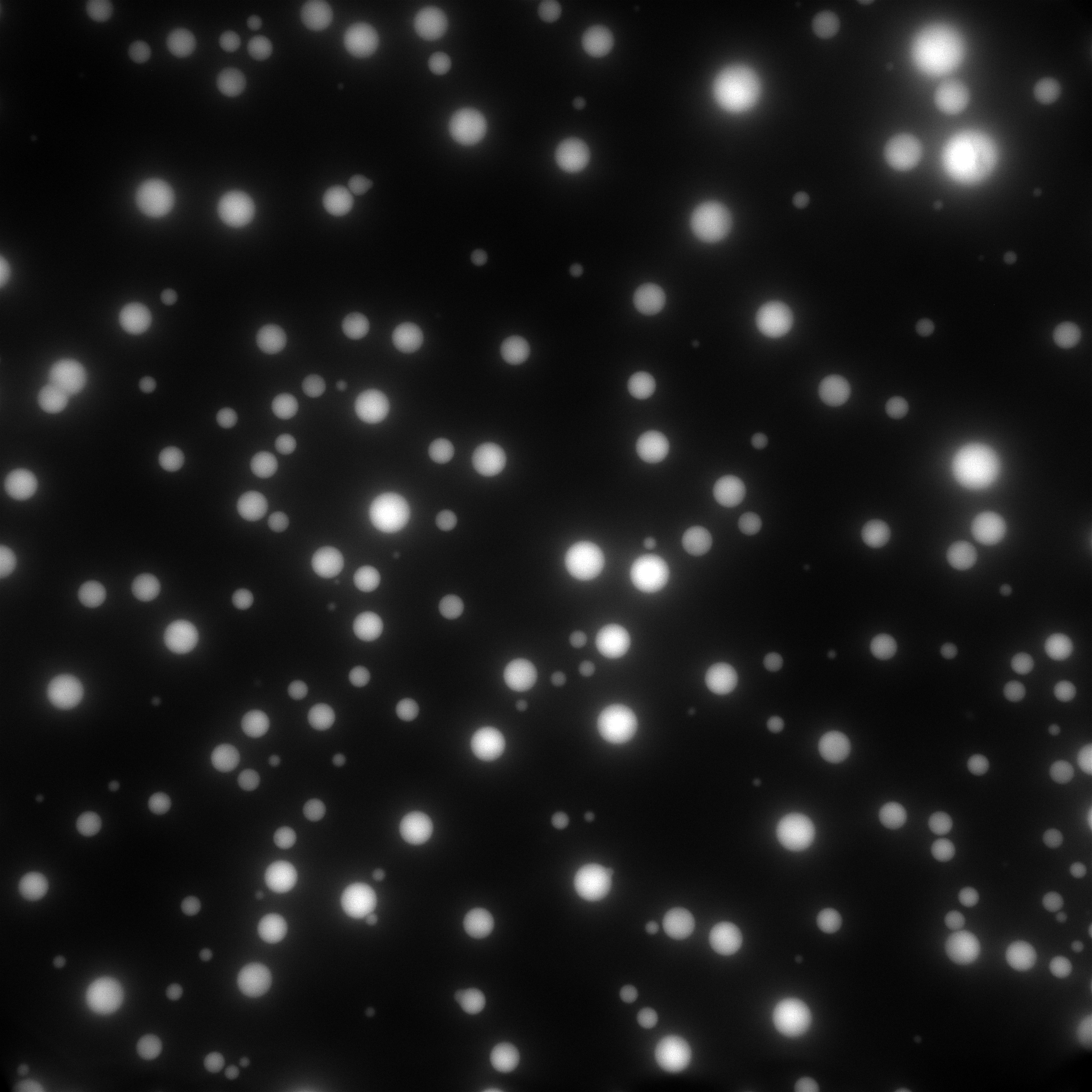

Supplement: Figure 3—figure supplement 1—source data 2. — Extracted numerical parameters are listed in the accompanying spreadsheet. [file elife-83543-fig3-figsupp1-data2.zip › Figure 3 - supplement 1 - source data 2/Figure 3 - supplement 1 - source data 2 - inactive - Lys5-24 - 1 h.tif]

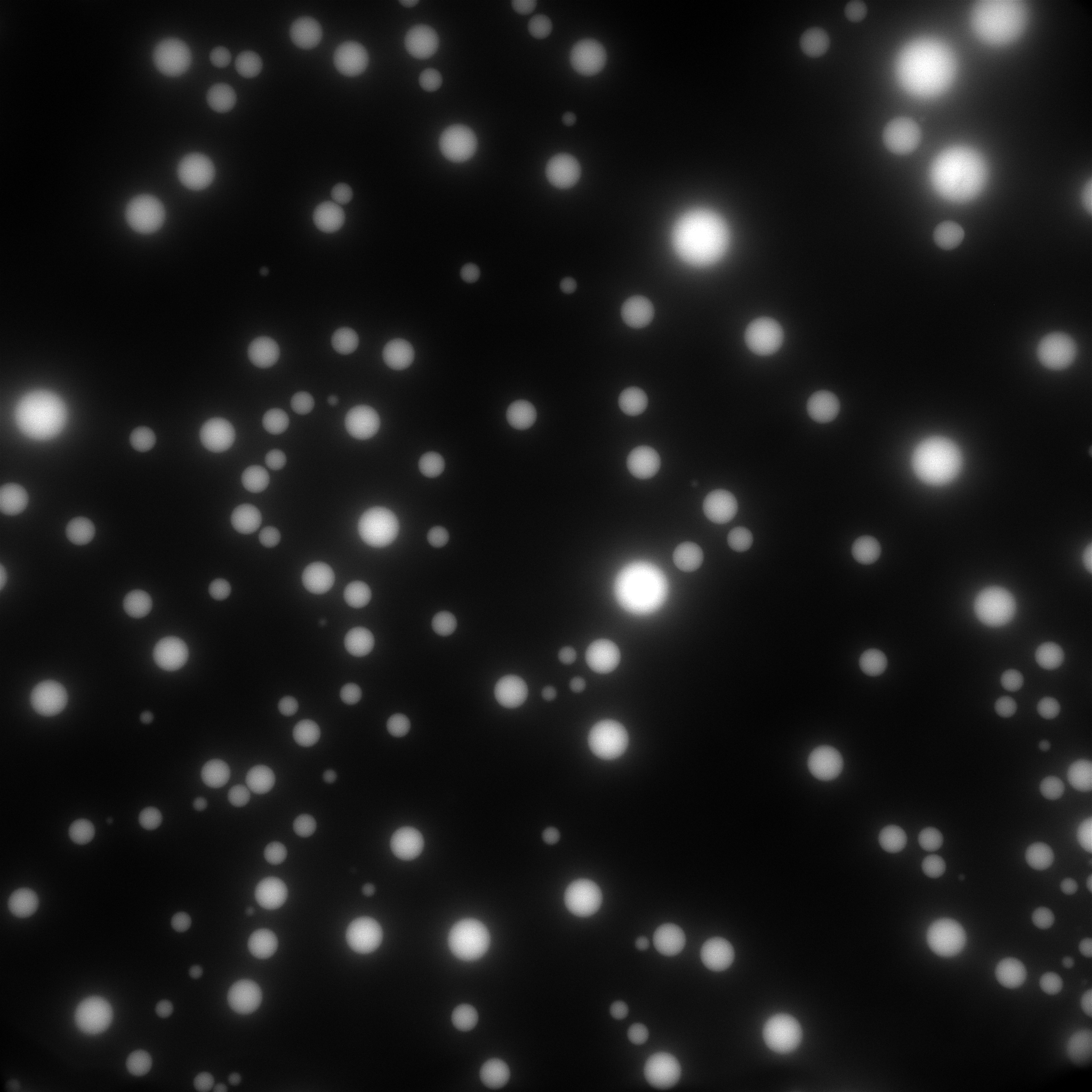

Supplement: Figure 3—figure supplement 1—source data 2. — Extracted numerical parameters are listed in the accompanying spreadsheet. [file elife-83543-fig3-figsupp1-data2.zip › Figure 3 - supplement 1 - source data 2/Figure 3 - supplement 1 - source data 2 - inactive - Lys5-24 - 2 h.tif]

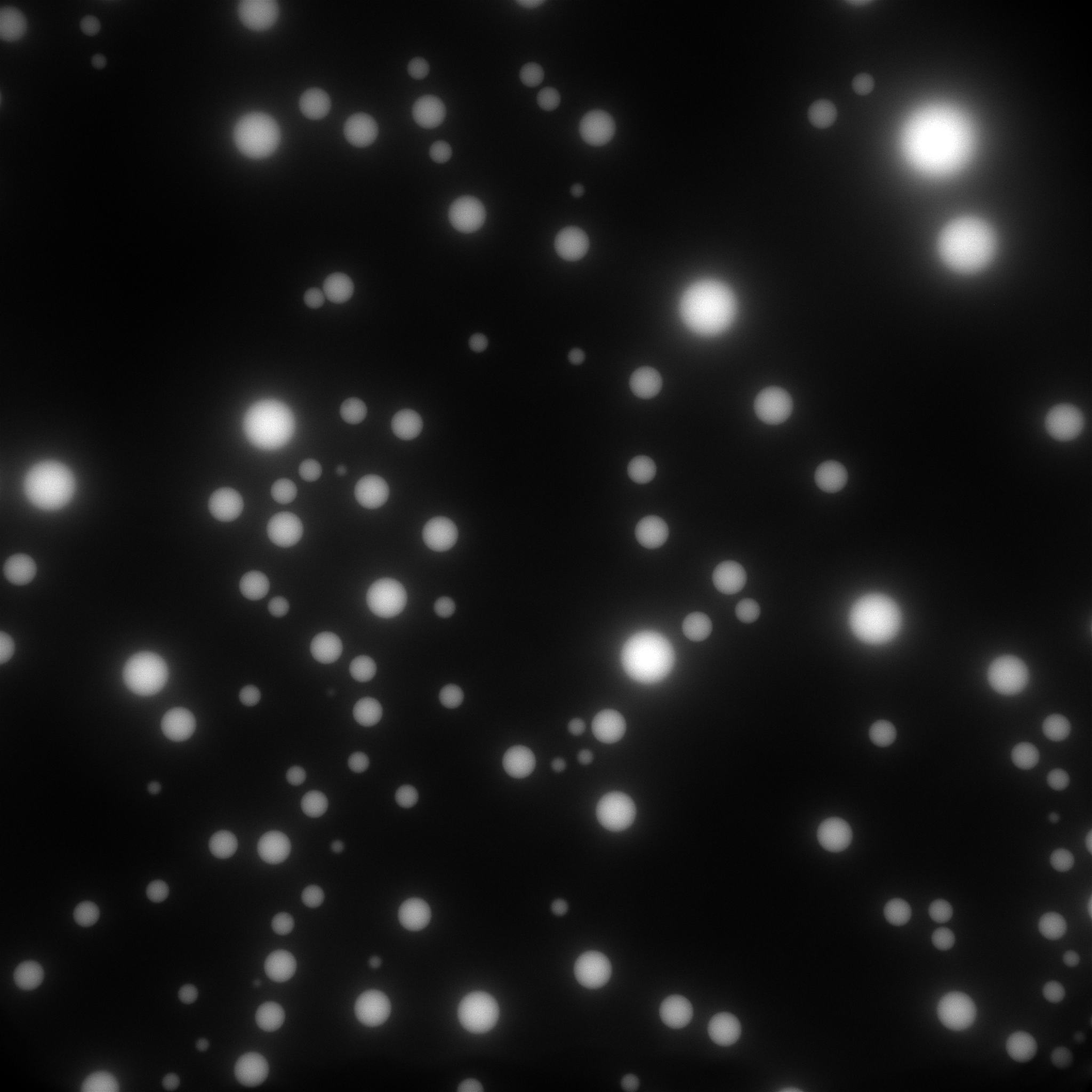

Supplement: Figure 3—figure supplement 1—source data 2. — Extracted numerical parameters are listed in the accompanying spreadsheet. [file elife-83543-fig3-figsupp1-data2.zip › Figure 3 - supplement 1 - source data 2/Figure 3 - supplement 1 - source data 2 - inactive - Lys5-24 - 24 h.tif]
